# Photo‐Triggered, Fast, and Fluorogenic Thiophene‐Based Cycloalkynes for the Bioorthogonal Fluorescent Labeling of 1,3‐Dipole‐Tagged Molecules in No‐Wash Conditions

**DOI:** 10.1002/anie.1383575

**Published:** 2026-06-23

**Authors:** Jurgen Schulz, Zoeisha S. Chinoy, Tarek Khalaf, Yukang Li, Pengchen Ma, Dennis Svatunek, Kendall N. Houk, Frédéric Friscourt

**Affiliations:** ^1^ Institut Européen de Chimie et Biologie Université De Bordeaux Pessac France; ^2^ Institut Des Sciences Moléculaires Bordeaux France; ^3^ School of Chemistry Xi'an Jiaotong University Xi'an China; ^4^ Department of Chemistry and Biochemistry University of California Los Angeles USA; ^5^ Institute of Applied Synthetic Chemistry Vienna Austria

**Keywords:** 1,3‐dipolar cycloaddition, bioorthogonal reactions, click chemistry, fluorescence imaging, fluorogenic probes

## Abstract

Bioorthogonal reactions have revolutionized our way of performing chemistry in a highly complex biological environment. In particular, strain‐promoted 1,3‐dipolar cycloadditions, employing cyclooctyne probes in conjunction with azido‐reporters (strain‐promoted alkyne‐azide cycloaddition, SPAAC), have permitted the labeling and visualization of bio‐macromolecules in vitro, in living cells, as well as in animals. However, SPAAC's slow kinetics (< 1 M^−1^s^−1^), in combination with the necessity to eliminate the excess of the used fluorescent probes, have hampered its widespread application, especially for the real‐time imaging of low concentration targets. Here we describe two novel thiophene‐based cycloalkynes that not only exhibit very high kinetics toward a variety of 1,3‐dipoles (up to 1528 M^−1^s^−1^), but are also efficiently turned‐on (up to 150‐fold increase in brightness) upon reaction with their target. We demonstrated their fast and fluorogenic capabilities by monitoring the labeling overtime of a glycoprotein in physiological and no‐wash conditions, using as little as 5 µM of the probes and reaching full labeling in less than 15 min. These fluorogenic cycloalkynes significantly expand our chemical biology toolbox, and we anticipate them to open new avenues for the fast and real‐time imaging of biomolecules in complex environments.

## Graphical abstract

Thiophene is king! The fusion of thiophene rings to cycloalkynes super‐charged their copper‐free click chemistry applications, by (1) enhancing the alkyne reactivity (fastest cyclooctyne to date) and (2) rendering the alkyne fluorogenic (with fluorescence enhancement up to 150‐fold upon reaction), making these bioorthogonal reagents ideal for tracking copper‐free click reactions in no‐time.

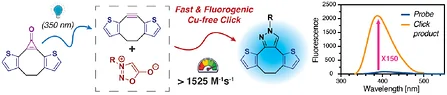

## 1 Introduction

**FIGURE 1:**
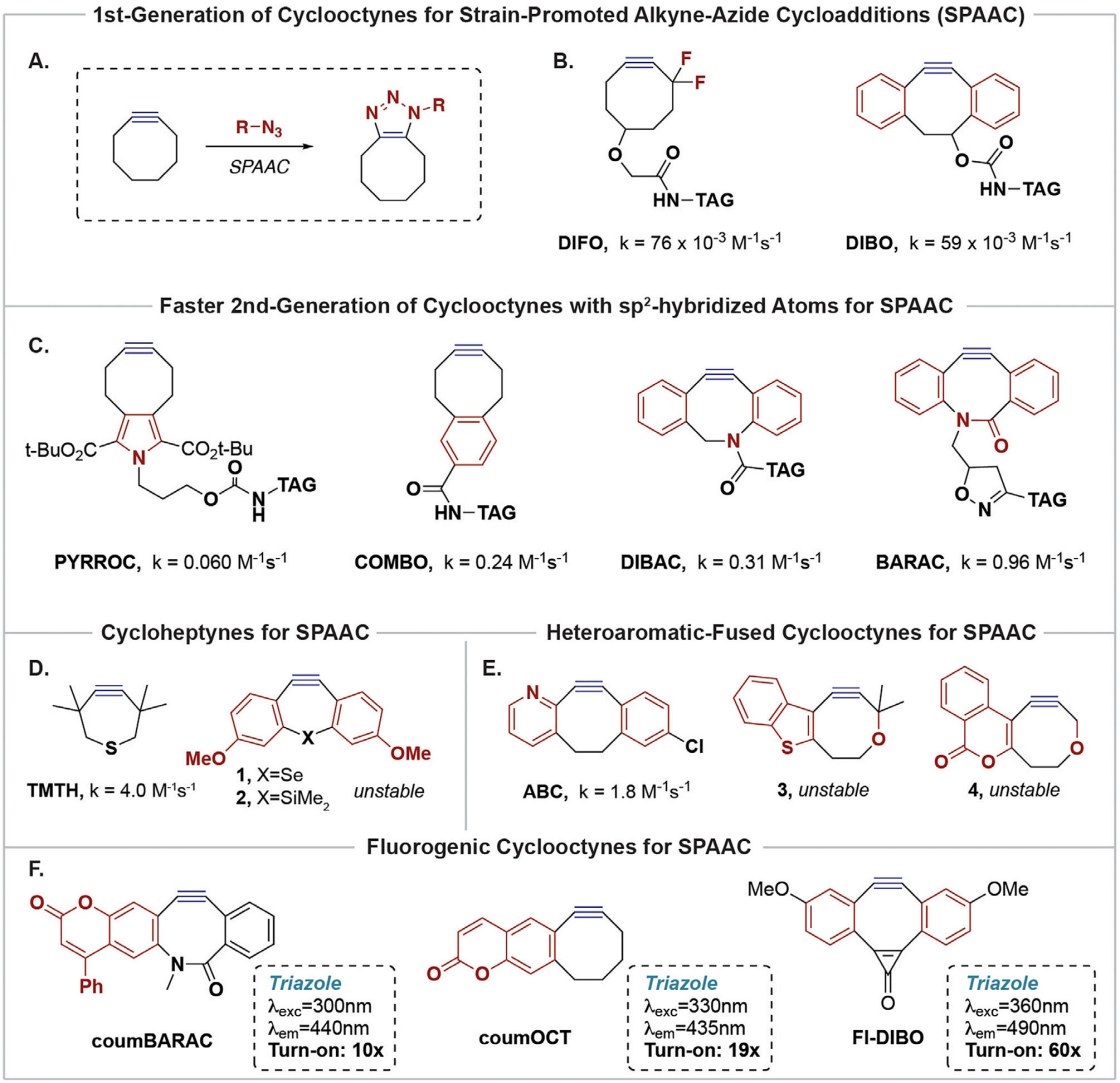
Developed cycloalkynes for fast or fluorogenic strain‐promoted alkyne‐azide cycloadditions (SPAAC). (A, B) First‐generation of cyclooctynes for SPAAC. (C) Second‐generation of cyclooctynes exhibiting faster SPAAC kinetics. (D) Cycloheptynes for enhanced reactivity. (E) Heteroaromatic‐fused cyclooctynes for faster SPAAC reactions. (F) Fluorogenic cyclooctynes and their turn‐on efficiency upon SPAAC. *k* represents the SPAAC second‐order rate constant for each cycloalkyne. TAG represents an unspecified biophysical probe, including, for instance, biotin or fluorescent dyes.

Over the past decade, bioorthogonal chemical reactions have revolutionized the fields of chemistry, chemical biology, and material sciences as they have allowed the manipulation and investigation of small and macromolecules alike, in highly complex systems [1, 2]. Metal‐free strain‐promoted alkyne‐azide cycloadditions (SPAAC, Figure 1A) are mild, efficient, and inert to the myriad of chemical functionalities found in vivo, making them the cornerstone of bioorthogonal reactions [3]. Accordingly, SPAAC was employed for the labeling and visualization of complex biomolecules, including nucleic acids, proteins, glycans, and lipids, directly in living cells [4, 5]. However, the poor kinetics of SPAAC reactions have hindered their applications, notably for the labeling of highly dilute biological targets in vivo.

To enhance the SPAAC reactivity, tailored cyclooctynes have been designed. The Bertozzi group has, for example, introduced a gem‐difluoro functionality at the propargylic position of the alkyne moiety (DIFO, *k* = 0.076 M^−1^s^−1^, Figure 1B), accelerating the cycloaddition by 60‐fold as compared to the unfluorinated cyclooctyne OCT [6]. Boons and co‐workers opted for increasing the strain of the alkyne by fusing two benzene rings to the cyclooctyne scaffold (DIBO, *k* = 0.059 M^−1^s^−1^, Figure 1B) [7]. Introducing sp^2^‐hybridized atoms within the cyclooctyne framework turned out to be a reliable strategy to enhance SPAAC kinetics, leading to many new faster cyclooctynes, including PYRROC (*k* = 0.060 M^−1^s^−1^) [8], COMBO (*k* = 0.24 M^−1^s^−1^) [9], DIBAC (*k* = 0.31 M^−1^s^−1^) [10], and BARAC (*k* = 0.96 M^−1^s^−1^) [11] (Figure 1C). Due to its fast kinetics, BARAC could be utilized, at very low concentrations, for the labeling of cell‐surface azido‐glycans in living cells. Further increase in the alkyne strain could be achieved by decreasing the size of the cycloalkyne, and a series of cycloheptynes was reported, namely tetramethylthiacycloheptyne (TMTH) [12], dibenzoselenacycloheptyne **1**, and dibenzosilacycloheptyne **2** (Figure 1D) [13, 14]. While these cycloheptynes clearly exhibited faster reaction rates toward azides, they were also found to be highly unstable, especially in water, limiting their potential applications.

Recent studies have elegantly exploited the use of fusing aromatic heterocycles to the cyclooctyne framework to combine increased ring strain and electronic tuning for enhanced SPAAC kinetics. Raines and co‐workers substituted one of the benzene rings of DIBO by a pyridine, making the cyclooctyne ABC highly reactive toward azides (*k* = 1.8 M^−1^s^−1^, 30‐fold faster than DIBO), while remaining stable (Figure 1E) [15]. In addition, Balova and co‐workers developed benzothiophene‐ and isocoumarin‐fused cyclooctynes **3** and **4** (Figure 1E); however, in contrast to ABC, these compounds were found unstable and could not be isolated [16]. Despite these tremendous synthetic efforts, SPAAC kinetics remain generally poor for most cyclooctynes. Consequently, high concentrations of probes are often needed for reliable fluorescent labeling of biomolecules in living cells, which in turn requires extensive washing steps before imaging to avoid unwanted fluorescence background.

To address this major drawback, fluorogenic SPAAC reagents that increase in fluorescence upon cycloaddition have recently been developed for maximizing signal over background, expanding the bioorthogonal chemistry toolbox [17]. Although numerous fluorogenic azido‐probes have been prepared by exploiting the azide's ability to quench known fluorophores, only three fluorogenic cyclooctynes have been reported to date. Inspired by the known capability of terminal alkyne to fluorescently quench coumarin [18], the groups of Bertozzi and Wang have elegantly prepared the fluorogenic cyclooctynes coumBARAC [19] and coumOCT [20], respectively (Figure 1F), by fusing a coumarin dye to the cyclooctyne ring. While both cyclooctynes exhibited weak fluorescence, formation of the triazole cycloadducts only led to a moderate increase in blue fluorescence (10‐fold for coumBARAC and 20‐fold for coumOCT). In contrast, we developed a fluorogenic dibenzocyclooctyne, Fl‐DIBO (Figure 1F), which, upon reaction with 1,3‐dipoles, led to the formation of cycloadducts exhibiting up to 240‐fold fluorescence enhancement (with quantum yields up to 0.47) [21, 22, 23]. However, like most cyclooctynes, Fl‐DIBO reacted rather slowly with azides (*k* = 0.019 M^−1^s^−1^). Consequently, we envisaged that appropriate selection of the type of rings fused to the cyclooctyne framework might enhance the reactivity of the cyclooctyne while maintaining its fluorogenic capabilities.

Herein, we report on the synthesis, reactivity, and biological evaluation of dithiophene‐cyclooctyne (DTPO) and ‐silacycloheptyne (DTPH), as a novel class of rapid fluorogenic bioorthogonal reagents. DTPO not only displayed exceptional kinetics toward 1,3‐dipoles (up to 1528 M^−1^s^−1^) but also showed exciting fluorogenic behavior, with cycloadducts exhibiting up to 150‐fold fluorescence enhancement as compared to the starting cyclooctyne. DFT calculations revealed that these cycloalkynes’ unusual high reactivity was due to ring strain and orbital interaction. As a result, DTPO and DTPH could fluorescently label glycoproteins in less than 15 min at low concentration without the need of washing steps, highlighting the promising applications of these novel cycloalkynes.

## 2 Results and Discussion

### 2.1 Design of the Cycloalkyne Probes

We reasoned that fusing 5‐membered thiophene rings to the cyclooctyne framework would be beneficial for both its reactivity and photophysical properties. On one hand, since the length of C = C bonds of thiophene (1.37 Å) are shorter than those of benzene (1.40 Å), the presence of the thiophene should contract the size of the cyclooctyne, with a concomitant increase in the alkyne strain. On the other hand, the presence of an ortho‐flagpole hydrogen on most dibenzocyclooctynes has been implicated in their reduced reactivity with 1,3‐dipoles due to steric hindrance [24]. Thus, placing the S‐atom of the thiophene ring at the homopropargylic position should alleviate potential steric penalty for cycloaddition reactions. Finally, thiophenes are key structural elements of optoelectronic materials due to their unique electronic and large molar absorptivity properties [25], making them ideal units to be introduced to convey appealing photophysical characteristics to cyclooctyne reagents.

**FIGURE 2:**
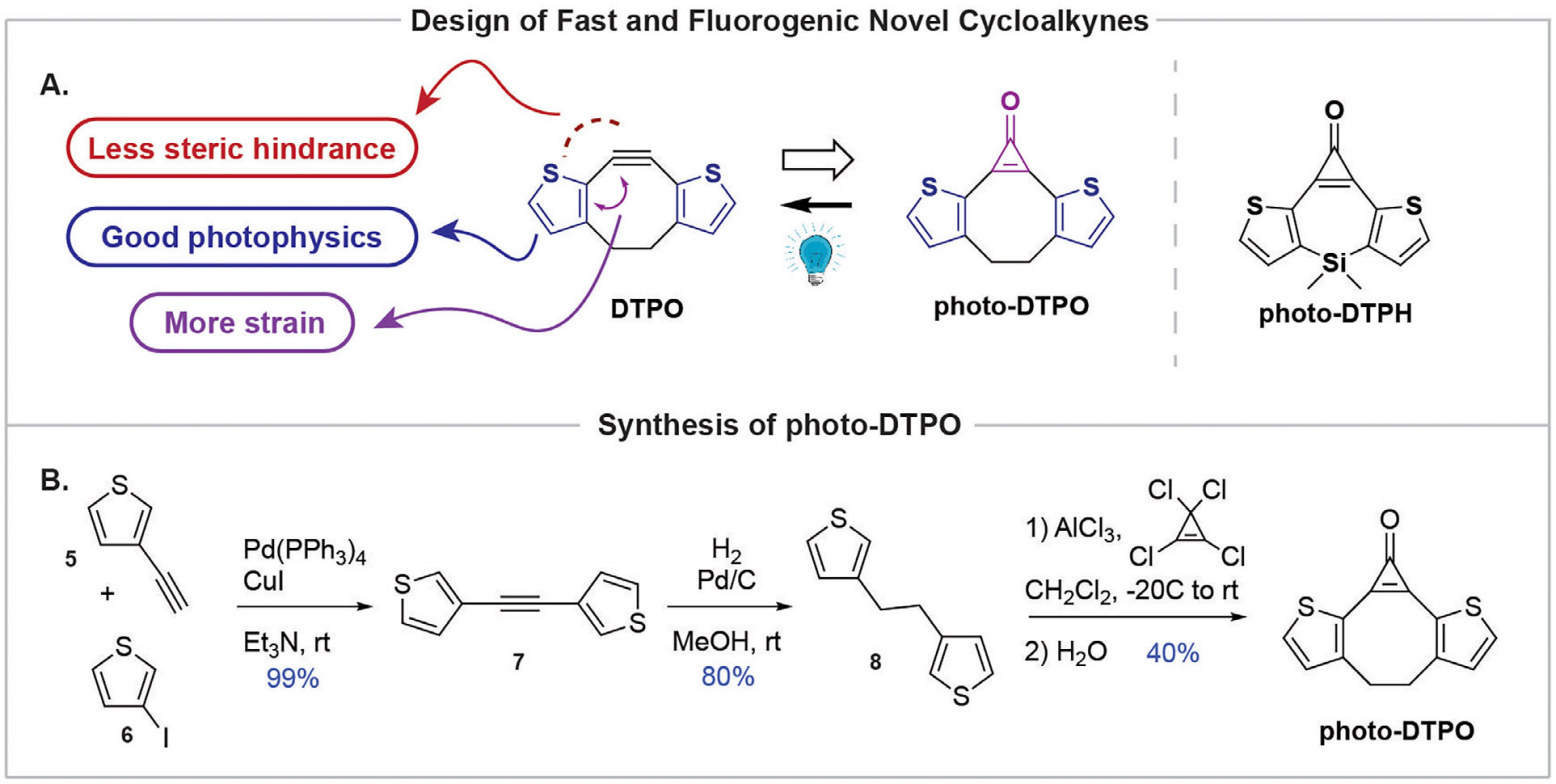
(A) Design of the cyclooctyne DTPO for exquisite reactivity and fluorogenic properties under SPAAC conditions and its synthetic precursor photo‐DTPO. (B) Chemical synthesis of photo‐DTPO.

Past studies have highlighted a clear correlation between the alkyne strain and the stability of the molecule. Therefore, we and others have exploited the use of a cyclopropenone moiety as a temporary protecting group of the alkyne functionality to prepare and isolate highly reactive cycloalkynes [13, 26, 27, 28]. An attractive feature of the cyclopropenone moiety is its deprotection under mild photo‐irradiation, classically in the UV region (350 nm) or under two‐photon absorption (800 nm) [29], fully compatible with live cell experiment (no cytotoxic effect reported) [26], allowing the generation of the cycloalkyne in a spatial and temporal manner. Consequently, we envisaged to synthesize DTPO as its cyclopropenone‐caged precursor, photo‐DTPO. Following the same principles, we decided to also prepare the cyclopropenone‐caged cycloheptyne analogue, photo‐DTPH, for increased reactivity (Figure 2A).

### 2.2 Synthesis and Stability Study of the Probes

Photo‐DTPO was prepared in an efficient three‐step synthesis (Figure 2B). Briefly, Sonogashira cross‐coupling reaction between 3‐ethynylthiophene (**5**) and 3‐iodothiophene (**6**) led to the quantitative formation of the symmetrical dithiophene‐alkyne **7**. Hydrogenation of **7** over Pd/C in methanol generated the dithiophene‐alkane **8** in 80% yield. Finally, Friedel–Crafts alkylation of **8** with tetrachlorocyclopropene in the presence of aluminum trichloride, followed by hydrolysis, produced the desired photo‐DTPO in a reasonable 40% yield. Similarly, photo‐DTPH was synthesized in only two steps in 17% overall yield, entailing the preparation of the dithiophene‐silane **S1** by reacting 3‐bromothiophene (**S2**) and dimethylchlorosilane (**S3**) in the presence of n‐butyl lithium, followed by the Friedel–Crafts alkylation with tetrachlorocyclopropene (Scheme S1). Both photo‐caged reagents were found to be highly stable in organic solution and in phosphate buffer saline (PBS, pH 7.4) as no decomposition was observed for over 48 h. To further challenge their stability and bioorthogonality, photo‐DTPO (50 µM) and photo‐DTPH (50 µM) were incubated in a 1 mM solution of the pseudo‐tripeptide glutathione in PBS pH 7.4, with 20% and 30% DMF (for solubility purposes), respectively, and monitored overtime. While photo‐DTPH exhibited a slight degradation overtime (less than 10% in 6 h) photo‐DTPO showed no sign of decomposition over 6 h (Figures S1–S4).

To assess the stability and reactivity of the cycloalkyne reagents, we first attempted to decarbonylate photo‐DTPO. A 50 µM solution of photo‐DTPO in a mixture of dichloromethane and methanol (2:1) was irradiated at 350 nm and monitored overtime by absorption spectroscopy. After only 50 s, the cyclopropenone characteristic peaks at 345 and 360 nm of photo‐DTPO fully disappeared, with the concomitant appearance of a new absorption peak at 325 nm, representative of the formation of the corresponding cyclooctyne (Figure S6). However, efforts to isolate DTPO on a preparative scale failed, and careful monitoring of a freshly prepared DTPO solution showed a half‐life of 55 min (Figure S5). Unsurprisingly, attempts to isolate but also detect DTPH, upon photo‐uncaging at 350 nm, were unsuccessful albeit with clear removal of the cyclopropenone moiety. All together, these results suggest that both DTPO and DTPH are highly reactive, as anticipated by their design, leading to their lack of long‐term stability in solution.

### 2.3 Cycloadduct Formations and Fluorogenic Properties

**FIGURE 3:**
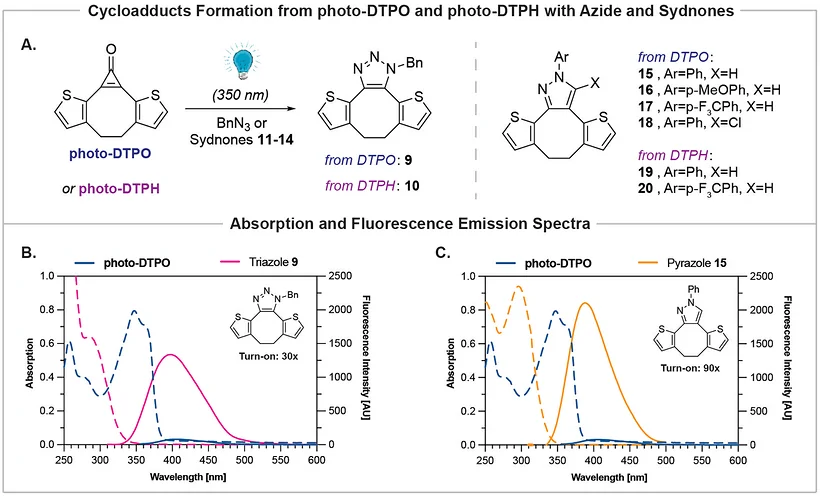
(A) Cycloadducts formation upon trapping DTPO and DTPH, following their photo‐uncaging, with benzyl azide and various sydnones. (B, C) Absorption (dashed line) and fluorescence emission (solid line) of a 50 µM solution of photo‐DTPO, triazole **9,** and pyrazole **15** in CH_2_Cl_2_:CH_3_OH (2:1).

Due to the sensitivity of DTPO and DTPH, we chose to trap the generated cycloalkynes with various 1,3‐dipoles. Accordingly, a solution of photo‐DTPO in a mixture of dichloromethane and methanol (2:1) was irradiated at 350 nm in the presence of benzyl azide. To our delight, not only the corresponding triazole **9** could be obtained efficiently in 83% yield, it also exhibited fluorescence properties when excited at 285 nm with a maximum emission at 398 nm and a quantum yield of 0.057 (Figure 3A,B; Table 1), making photo‐DTPO and DTPO exciting metal‐free strain‐promoted fluorogenic probes due to their virtual non‐fluorescence (Table 1; Figures 3B, S6, and S7). Similarly, the reaction of photo‐DTPH with benzyl azide under photo‐irradiation led to the formation of the corresponding fluorescent triazole **10** in 82% yield, exhibiting a slight bathochromic shift of both excitation and emission as well as an improved fluorescence quantum yield of 0.082 as compared to triazole **9** (Table 1, Figures S8 and S9).

**TABLE 1:** Photophysical properties of photo‐DTPO and photo‐DTPH and their associated cycloadducts: Triazoles 9–10 and pyrazoles 15–20 in DCM:MeOH (2:1).

Entry	Compound	*λ*_abs_ [nm]	*ε*_abs_ [M^−1^.cm^−1^]^a^	*λ*_em_ [nm]	*Φ*_F_ ^b^	Stokes shift [nm]	Brightness^c^	FE^d^
1	Photo‐DTPO	347	5900 ± 500	405	0.003	58	18	1
2	Triazole **9**	285	10 300 ± 650	398	0.057	113	588	30
3	Photo‐DTPH	355	7600 ± 200	408	0.005	53	38	1
4	Triazole **10**	310	11 900 ± 200	405	0.082	95	976	25
5	Pyrazole **15**	295	21 300 ± 600	390	0.079	95	1683	90
6	Pyrazole **16**	295	24 700 ± 1300	390	0.084	95	2075	120
7	Pyrazole **17**	305	25 200 ± 700	395	0.105	85	2646	150
8	Pyrazole **18**	280	28 200 ± 2000	400	0.005	120	141	7
9	Pyrazole **19**	315	18 100 ± 300	395	0.128	80	2317	60
10	Pyrazole **20**	320	24 000 ± 600	395	0.139	73	3336	90

^a^ Determined at the corresponding *λ*_abs._

^b^ Fluorescence quantum yield, 2‐aminopyridine in 0.1N H_2_SO_4_ as standard.

^c^ Product *ε*_abs_.*Φ*_F._

^d^ Fluorescence enhancement of the fluorophore brightness relative to photo‐DTPO or photo‐DTPH.

Next, with the goal to impact the fluorescence properties of the cycloadducts, we decided to employ sydnones as 1,3‐dipoles [30]. Sydnones have the distinct advantage, over azides, to be structurally modulable at the N3‐ and C4‐position of the mesoionic ring. In addition, we and others have shown that sydnones can react in a metal‐free fashion with cyclooctynes to generate pyrazoles for the labeling and visualization of proteins [23, 31, 32, 33, 34], oligonucleotides [35], and complex glycans [36, 37]. Consequently, four structurally different sydnones **11–14** (Figure 3A) were selected on the basis of their varying electron density, and were reacted with DTPO and DTPH. Most generated pyrazoles displayed compelling photophysical properties with high coefficients of absorption (over 21 000 M^−1^cm^−1^), reasonable fluorescence quantum yields, and large Stokes shifts (over 90 nm) (Table 1, Figures S10–S15). While the presence of an electron‐donating substituent (Entry 6) at the N‐position of the pyrazole **16** had very little effect on the fluorescence properties, pyrazoles **17** and **20**, displaying an electron‐withdrawing group (Entry 7 and 10), interestingly exhibited a red‐shift of their excitation wavelengths (*λ*_exc_ = 305 and 320 nm, respectively) and an increase in fluorescence quantum yields (*Φ*_F_ = 0.105 and 0.139, respectively) as compared to the unsubstituted pyrazole **15** (Entry 5, *λ*_exc_ = 295 nm, *Φ*_F_ = 0.079, Figure 3C). Unfortunately, introduction of a chlorine atom on the pyrazole moiety (Entry 8) led to complete fluorescence quenching, probably through the heavy atom effect via intersystem crossing. Importantly, triazoles **9** and **10** as well as pyrazoles **15** and **19** were found completely stable in PBS pH 7.4 and in a 1 mM solution of glutathione in PBS pH 7.4 for over 6 h (Figures S16–S19). Collectively, these results reveal that DTPO and DTPH are novel bioorthogonal strain‐promoted fluorogenic probes that can generate strongly fluorescent products with high fluorescence enhancement (up to 150‐fold), highlighting their strong potential for the labeling of biomolecules with high signal‐to‐background ratio.

### 2.4 Kinetics Study of the Probes

High reactivity being a critical factor of bioorthogonal reagents, we set out to determine the second‐order rate constants of the cycloaddition reactions between DTPO and 1,3‐dipoles (Table 2, Figures S20–S25). Briefly, a solution of photo‐DTPO in a mixture of dichloromethane and methanol (2:1) was first uncaged by irradiation at 350 nm to generate the corresponding cyclooctyne. DTPO was then immediately introduced to a thermally equilibrated solution (21°C) of 1,3‐dipoles, and the formation of the corresponding cycloadducts was monitored by following the increase of fluorescence overtime. Both benzyl azide and 3‐phenyl sydnone (**11**) reacted extremely fast with DTPO (*k* ≃ 25 M^−1^s^−1^) at a rate 415‐fold faster than the previously reported reaction between the cyclooctyne DIBO [7] and benzyl azide and 14‐fold faster than the recently described ABC cyclooctyne [15]. Interestingly, adding an electron‐withdrawing substituent on the sydnone further enhanced the reaction kinetics, with *p*‐CF_3_‐phenyl sydnone (**13**) displaying a very high second‐order rate constant of 57 M^−1^s^−1^. In contrast, electron‐rich *p*‐MeO‐phenyl sydnone (**12**) reacted slower with DTPO (*k* = 12 M^−1^s^−1^). In addition, since the ultimate goal of DTPO is to be utilized for biological applications, and polar solvents have been shown to significantly impact SPAAC reaction rates [15, 38], kinetic experiments involving DTPO were also performed in PBS pH 7.4 with 20% DMF (Figures S24 and S25). Interestingly, cycloaddition of DTPO with sydnone **11** showed enhanced reactivity in PBS with a kinetic constant of 420 M^−1^s^−1^. The reaction rate was further improved by reacting DTPO with sydnone **13**, to a remarkable 1530 M^−1^s^−1^, values never seen before for strain‐promoted 1,3‐dipolar cycloadditions. Although one can expect DTPH to be even more reactive than DTPO, we were unable to determine its cycloaddition rate constants due to its lack of stability in solution.

**TABLE 2:** Second‐order rate constants for the cycloadditions of DTPO with benzyl azide and various sydnones 11–13 at 21°C in DCM:MeOH (2:1) or in PBS pH 7.4 (with 20% DMF).

Entry	1,3‐Dipole	*k* [M^−1^ s^−1^] in DCM:MeOH	*k* [M^−1^ s^−1^] in PBS:DMF
1	Benzyl azide	26.2 ± 0.7	N.D.
2	Sydnone **11**	24.6 ± 0.7	418.8 ± 8.2
3	Sydnone **12**	12.1 ± 0.4	N.D.
4	Sydnone **13**	56.8 ± 1.7	1528 ± 65

Abbreviation: N.D., not determined.

Lastly, to evaluate the efficiency of DTPO to label sydnones in complex aqueous dilute conditions, knowing its short half‐life but also its very high cycloaddition kinetics, we investigated the formation of pyrazole **15** and **17** in PBS pH 7.4 in the presence of 1 mM of glutathione. Accordingly, we compared the fluorescence intensity of a 20 µM solution of preformed pyrazole with the fluorescence intensity of the same pyrazole generated by incubating 20 µM of photo‐DTPO with 50 µM of the corresponding sydnone under irradiation, in 1 mM glutathione in PBS pH 7.4 (with 20% DMF for solubility) (Figure S26). Gratifyingly, over 75% conversion was observed for the formation of pyrazole **15** and over 80% in the case of pyrazole **17**, which is in good agreement with the observed isolated yields of these two pyrazoles in an organic solvent. Collectively, DTPO showed exceptional reactivity with a range of 1,3‐dipoles in both organic solvent and aqueous environment, as well as high conversion rates in complex aqueous dilute conditions, ideal features for the labeling of low abundance targets.

### 2.5 DFT Calculations

**FIGURE 4:**
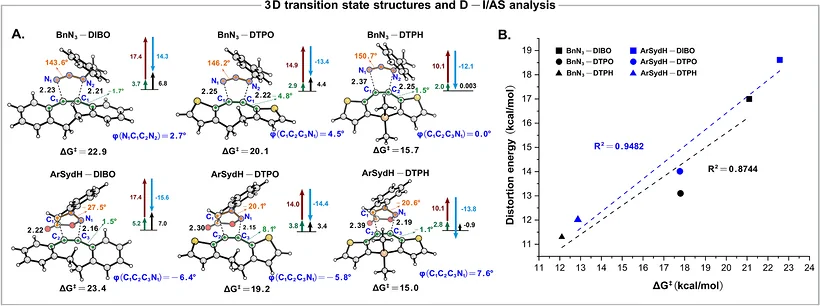
(A) 3D transition state structures and D‐I/AS analysis of cycloaddition reaction of benzyl azide (BnN_3_) and sydnone **11** (ArSydH) with cycloalkynes DIBO, DTPO, and DTPH. Red: distortion energy of 1,3‐dipoles; green: distortion energy of cycloalkynes; blue: interaction energy; black: activation energy; key angle and dihedral angles are labeled, transition state bond distances in Å. (B) Plot of distortion energy versus Gibbs free energies barriers.

Intrigued by the high reactivity of those cycloalkynes with two types of 1,3‐dipoles, we investigated the reaction mechanism by DFT calculations. As shown in the upper row of Figure 4A, starting from a reactants complex, the Gibbs free energy barriers for the cycloaddition of 1,3‐dipole benzyl azide with DIBO, DTPO, and DTPH, are 17.0, 13.1, and 11.3 kcal/mol, respectively. The bottom row presents the Gibbs free energy barriers for the cycloaddition reactions of 1,3‐dipole ArSydH **11** with DIBO, DTPO, and DTPH, which are 18.6, 14.0, and 12.0 kcal/mol, respectively. In agreement with the experiments, these results show that benzyl azide is slightly more reactive than sydnone **11** in all corresponding reactions, but more importantly that DTPO and DTPH are clearly more reactive than DIBO for both 1,3‐dipoles.

To gain further insights, we performed a distortion–interaction (D – I)/activation strain (AS) analysis [39]. Figure 4B shows the correlation between distortion energies and Gibbs free energies of activation. For both the benzyl azide and sydnone **11**, a good correlation was found between distortion energies and activation energies with correlation coefficients (*R* ^2^) of 0.9482 and 0.8744, respectively. More reactive DTPO and DTPH show significantly reduced distortion energies compared to DIBO, indicating increased strain and less steric repulsion, ultimately leading to lower barriers.

### 2.6 Biological Evaluation of the Probes

**FIGURE 5:**
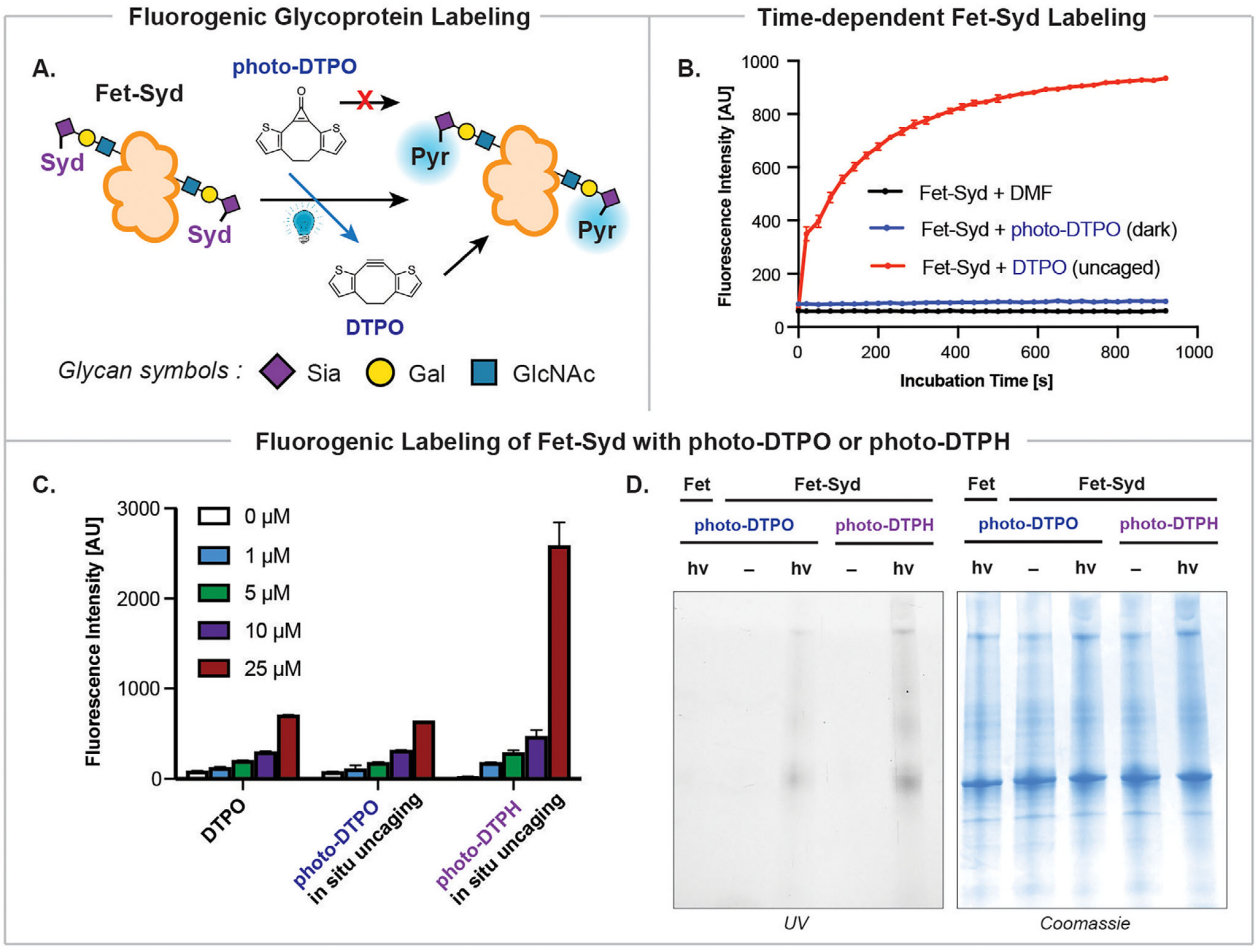
(A) Fluorogenic labeling of the glycoprotein sydnone‐modified fetuin (Fet‐Syd); Pyr: pyrazole, Sia: sialic acid, Gal: galactose, GlcNAc: *N*‐acetyl‐glucosamine. (B) Overtime fluorescence monitoring of Fet‐Syd labeling with photo‐DTPO (25 µM, dark), freshly uncaged DTPO (25 µM) or no reagent (DMF). (C) Fluorescence labeling of Fet‐Syd with freshly uncaged DTPO (0–25 µM), in situ uncaged photo‐DTPO (0–25 µM) or in situ uncaged photo‐DTPH (0–25 µM) for 15 min. (D) In‐gel visualization of Fet‐Syd (30%) or Fet (30%) in U2OS cell lysate incubated for 15 min at 37°C with photo‐DTPO (25 µM) or photo‐DTPH (25 µM), in situ uncaged at 350 nm (*hv*) or not (–).

To demonstrate the capability of both DTPO and DTPH to fluorescently label biomolecules in physiological conditions, we first prepared the sydnone‐modified glycoprotein fetuin (Fet‐Syd). Briefly, asialofetuin was treated with a CMP‐sialic acid donor, containing a sydnone at its 9‐position, in the presence of the recombinant human sialyltransferase ST6GAL1 in sodium cacodylate buffer in order to introduce the sydnone reporter to the sialoglycan of fetuin, as we previously described (Scheme S2) [36]. Next, Fet‐Syd was incubated in PBS (pH 7.4) at room temperature with a solution of freshly uncaged DTPO (25 µM final concentration in PBS containing 20% of DMF) and labeling of the glycoprotein was monitored overtime by fluorescence spectroscopy (Figure 5B). The labeling reaction showed a clear time‐dependent increase of fluorescence intensity, with an apparent plateau after 15 min of incubation time, confirming the fast kinetics of DTPO with the sydnone reporter in physiological conditions. Importantly, reaction of Fet‐Syd with photo‐DTPO in the dark showed no detectable fluorescence even upon prolonged incubation time, highlighting that the uncaging of the cyclooctyne is required for the fast fluorogenic labeling to occur, a key feature for potential spatial‐ and temporal‐controlled labeling experiments.

The concentration‐dependency of the fluorescence labeling of Fet‐Syd was also examined by incubating for 15 min various concentrations of either freshly prepared DTPO or in situ generated DTPO upon short photo‐irradiation (1 min) of the solution containing both the protein and photo‐DTPO (Figure 5C). Detectable levels of fluorescence labeling were consistently observed when DTPO was employed at as low as 5 µM, but optimal results were obtained at concentration ranging from 10 to 25 µM. Interestingly, light‐activation of photo‐DTPO either before injection or in situ resulted in equally efficient protein fluorescent labeling. Consequently, we also investigated the use of photo‐DTPH for the labeling of sydnone‐modified fetuin following the above in situ protocol. Gratifyingly, labeling of Fet‐Syd with 25 µM of photo‐DTPH led to a 4‐fold increase in fluorescence signal as compared to photo‐DTPO, probably due to its suspected higher kinetics and the higher fluorescence quantum yield of the corresponding cycloadduct.

To confirm the specificity of the observed fluorescence signal in a more stringent biological environment, human bone osteosarcoma (U2OS) cell lysate was spiked with 30% of sydnone‐modified fetuin (Fet‐Syd), or regular fetuin (Fet) for control, and incubated at 37°C for 15 min with 25 µM of either photo‐DTPO or photo‐DTPH, in situ uncaged with UV‐light (350 nm) or not (–) and was analyzed by in‐gel fluorescence imaging without any washing steps (Figure 5D). Fluorescent labeling was clearly observed when sydnone‐modified fetuin was employed in the presence of either photo‐DTPO or photo‐DTPH that were in situ uncaged (lane 3 and 5), with a stronger labeling for photo‐DTPH, as seen in Figure 5C. Importantly, no fluorescence was detected in the control experiments (lane 1, 2, and 4), demonstrating the photo‐control as well as the exquisite specificity of protein labeling by photo‐DTPO and photo‐DTPH in no‐wash conditions.

## 3 Conclusions

We have developed two novel fluorogenic cycloalkynes for the rapid bioorthogonal fluorescent labeling of biomolecules in no‐wash conditions. The cyclopropenone‐caged cyclooalkynes photo‐DTPO and photo‐DTPH have the advantage to be easily prepared in three steps or less from commercially available compounds and can be stored for months without observable degradation. Once uncaged, both cycloalkynes DTPO and DTPH react extremely fast with a variety of bioorthogonal 1,3‐dipoles such as azides and sydnones, displaying kinetics unseen before in strain‐promoted 1,3‐dipolar cycloadditions (up to 1528 M^−1^s^−1^). A computational investigation showed that the increased reactivity is a result of a reduced distortion energy in the transition state, introduced by substituting the common benzo‐group by thiophenes.

In addition, the cycloalkyne probes DTPO and DTPH, as well as their cyclopropenone‐caged precursors, are virtually non‐fluorescent (*Φ*_F_ < 0.005) but give rise to fluorescence when reacted with 1,3‐dipoles. The resulting cycloadducts exhibit compelling photophysical properties, including large Stokes shifts (up to 110 nm), high coefficients of absorption and good fluorescence quantum yields, leading to an increase of a 150‐fold in brightness as compared to the unreacted starting probes. However, their required excitation in the UV region (up to 320 nm) currently precludes their utilization in living systems. Nevertheless, the fast and fluorogenic nature of these cycloalkynes allowed us to fluorescently label the bioorthogonally tagged glycoprotein fetuin in cell lysate and no‐wash conditions with exquisite specificity using as little as 25 µM of the probes and in less than 15 min, making the fluorescent monitoring of biomolecules simple and fast, ideal for future high‐throughput utilization. Finally, the use of cyclopropenone photo‐cage can further expand the range of applications of these novel probes by adding a spatial and temporal control over their activation, a most needed feature for high precision labeling.

## Author Contributions

**Jurgen Schulz**: investigation, formal analysis. **Zoeisha S. Chinoy**: investigation, formal analysis, writing – original draft. **Tarek Khalaf**: investigation. **Yukang Li**: investigation, formal analysis. **Pengchen Ma**: writing – review and editing, investigation, formal analysis, supervision. **Dennis Svatunek**: investigation, formal analysis, writing – review and editing. **Kendall N. Houk**: supervision, funding acquisition, writing – review and editing. **Frédéric Friscourt**: conceptualization, data curation, investigation, formal analysis, supervision, funding acquisition, writing – original draft, writing – review and editing.

## Conflicts of Interest

The authors declare no conflicts of interest.

## Supporting information




**Supporting File**: anie73313‐sup‐0001‐SuppMat.pdf.

## Data Availability

The data that support the findings of this study are available in the Supporting Information of this article.
