# Supplementary material for: Photo‐Triggered, Fast, and Fluorogenic Thiophene‐Based Cycloalkynes for the Bioorthogonal Fluorescent Labeling of 1,3‐Dipole‐Tagged Molecules in No‐Wash Conditions
Source: Angew Chem Int Ed Engl. 2026 Jun 23;65(35):e1383575. doi: 10.1002/anie.1383575 (PMC13502660; doi:10.1002/anie.1383575)
Supplement: Supplementary file 1 [file ANIE-65-e1383575-s001.pdf]

Supporting Information

Photo-triggered, Fast and Fluorogenic Thiophene-Based Cycloalkynes for the Bioorthogonal Fluorescent Labeling of 1,3-Dipole-Tagged Molecules in No-Wash Conditions

Jurgen Schulz[1,2], Zoeisha S. Chinoy[1,2], Tarek Khalaf[1,2], Yukang Li[3], Pengchen Ma[3,4], Dennis Svatunek[4,5], Kendall N. Houk[4], and Frédéric Friscourt*[1,2]

[1]Institut Européen de Chimie et Biologie, Université de Bordeaux, 2 rue Robert Escarpit, 33607 Pessac, France.

[2] Institut des Sciences Moléculaires, CNRS UMR5255, Bordeaux, France

[3] School of Chemistry, Xi'an Jiaotong University, Xi'an 710049, China

[4] Department of Chemistry and Biochemistry, University of California, Los Angeles, California 90095, United States

[5] Institute of Applied Synthetic Chemistry, TU Wien, 1060 Vienna, Austria

* Email: f.friscourt@iecb.u-bordeaux.fr

Table of Contents

Materials and Methods S2

Synthetic Procedures (Scheme S1) S3

Stability Studies of photo-DTPO and photo-DTPH (Figures S1-S4) S9

Absorption and Fluorescence Spectra (Figures S6-S15) S11

Stability Studies of triazoles and pyrazoles (Figures S16-S19) S15

Kinetic Measurements (Figures S20-S26) S17

DFT Calculations (Figures S27-S29) S21

Protein Modification (Scheme S2) S40

^{1}H and ^{13}C NMR Spectra S41

References S60

Materials and Methods

All reactions were carried out under a dry argon environment. All solvents were of reagent grade and used as received. Dichloromethane and tetrahydrofuran were dried over alumina columns under nitrogen through a solvent purification system. Chemicals and reagents were used as commercially supplied without any further purification unless otherwise stated. Room temperature refers to ambient temperature (20−22 °C). Column chromatography was carried out using silica gel G60 (Fluka analytical, 230−400 mesh, 40−63 μm particle size, 60 Å) as the stationary phase.

NMR Spectroscopy

^{1}H, ^{13}C, ^{19}F, and ^{29}Si NMR spectra were recorded on a Brüker 300 MHz or 400 MHz spectrometer. Chemical shifts are reported in δ units, parts per million (ppm) downfield from TMS. Coupling constants (*J*) are reported in hertz (Hz) without adjustments. Splitting patterns are designed as follows: s, singlet; d, doublet; t, triplet; dd, doublet of doublets; dt, doublet of triplets; td, triplet of doublets; ddd, doublet of doublet of doublets; tt, triplet of triplets; m, multiplet; br, broad. Various 2D techniques experiments were used to establish the structures and to assign the signals.

Mass Analysis

High-resolution mass spectra were obtained with an electrospray ionization Thermo Exactive orbitrap mass spectrometer.

Photoreactions

Photoreactions were performed on a Rayonet RMR 600 photoreactor using 4×350 nm lamps (4W).

Absorption and Fluorescence Measurements

UV−Vis and Fluorescence spectra were recorded at 25 ± 0.1 °C using a SpectraMax M2E Multi-Mode Cuvette/Microplate Reader from molecular devices using a cuvette with 1 cm path length.

Quantum Yield Determination

Quantum yields were determined from the slope of the integrated fluorescence emission between 320 and 600 nm (at λ_{max} excitation of each compound) versus absorbance using 2-amino pyridine in 0.1 N H2SO4 (Φ_F = 0.6) as fluorescence standard. For each compound, four data points were acquired with absorbances ranging between 0.1 and 0.7 (l = 1 cm).

Synthetic Procedures

3-(3'-Thienylethynyl)thiophene 7.

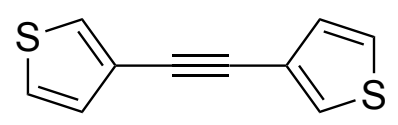

Tetrakis(triphenylphosphine)palladium(0) (120 mg, 0.10 mmol) and copper(I) iodide (38 mg, 0.20 mmol) were added to a solution of 3-iodothiophene (407 μL, 4.00 mmol) and 3-ethynylthiophene (394 μL, 4.00 mmol) in triethylamine (24 mL) at 0 °C. The reaction mixture was stirred at room temperature overnight and then concentrated under reduced pressure. The residue was then purified by flash column chromatography on silica gel (26 g) using petroleum ether to afford pure thiophene **7** (765 mg, quant.) as a white solid: ^{1}H NMR (300 MHz, $CDCl_3$): δ 7.19 (dd, *J* = 5.0, 1.2 Hz, 2H), 7.30 (dd, *J* = 5.0, 3.0 Hz, 2H), 7.50 (dd, *J* = 3.0, 1.2 Hz, 2H); ^{13}C NMR (75 MHz, $CDCl_3$): δ 84.1 (2×C), 122.3 (2×C), 125.5 (2×CH), 128.6 (2×CH), 130.0 (2×CH); HRMS FD (m/z): [$M^{+\bullet}$] calcd for $C_{10}H_6S_2$, 189.9911; found 189.9910.

3-[2-(3'-Thienyl)ethyl]thiophene 8.

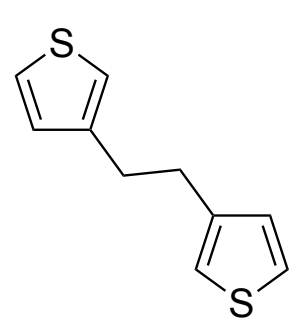

A solution of thiophene **7** (571 mg, 3.0 mmol) and palladium on carbon (10%, 319 mg, 0.3 mmol) in methanol (15 mL) was stirred at room temperature under H_2 (1 atm) for 15 h. The reaction mixture was then filtered through Celite and washed with dichloromethane (20 mL). The filtrate was then concentrated under reduced pressure and the residue was purified by flash column chromatography on silica gel (18 g) using petroleum ether to afford the pure thiophene **8** (467 mg, 80%) as a white solid: ^{1}H NMR (300 MHz, $CDCl_3$): δ 2.99 (s, 4H), 6.96 (d, *J* = 4.3 Hz, 4H), 7.27–7.29 (m, 2H); ^{13}C NMR (75 MHz, $CDCl_3$): δ 31.4 (2×CH_2), 120.5 (2×CH), 125.4 (2×CH), 128.3 (2×CH), 142.2 (2×C); HRMS FD (m/z): [$M^{+\bullet}$] calcd for $C_{10}H_{10}S_2$, 194.0224; found 194.0227.

3,4-Dihydro-cyclopropa[2'',3'':7,8]thieno[3',2':5,6]cycloocta[1,2-b]thiophen-1''-one photo-DTPO.

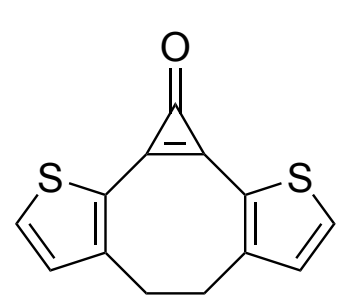

A solution of aluminum chloride (400 mg, 3.0 mmol) and tetrachlorocyclopropene (147 μL, 1.2 mmol) in dichloromethane (15 mL) was stirred at room temperature for 30 min. The reaction mixture was then cooled to –20 °C and a solution of thiophene **8** (194 mg, 1.0 mmol) in dichloromethane (5 mL) was added. The reaction mixture was stirred at –20 °C for 1 h, was then allowed to warm to room temperature over a period of 1 h and was stirred for an extra 1 h at room temperature. An aqueous solution of HCl (1N, 20 mL) was

then added and the reaction mixture was stirred for an extra 30 min. The organic layer was then extracted with dichloromethane (3×10 mL), dried over $MgSO_4$ and concentrated under reduced pressure. The residue was then purified by flash column chromatography on silica gel (9 g) using a mixture of dichloromethane and methyl tert-butyl ether (97:3) to afford pure **photo-DTPO** (98 mg, 40%) as a yellow/orange solid: ^{1}H NMR (300 MHz, $CDCl_3$) δ 2.66 (brs, 2H), 3.66 (brs, 2H), 7.07 (d, *J* = 5.0 Hz, 2H), 7.63 (d, *J* = 5.0 Hz, 2H); ^{13}C NMR (75 MHz, $CDCl_3$) δ 32.3 (2×CH_2), 122.2 (2×C), 131.0 (2×CH), 132.9 (2×CH), 134.0 (2×C), 147.4 (2×C), 152.0 (C=O); HRMS ESI (m/z): [$M+H^+$] calcd for $C_{13}H_9OS_2$, 245.0095; found 245.0092.

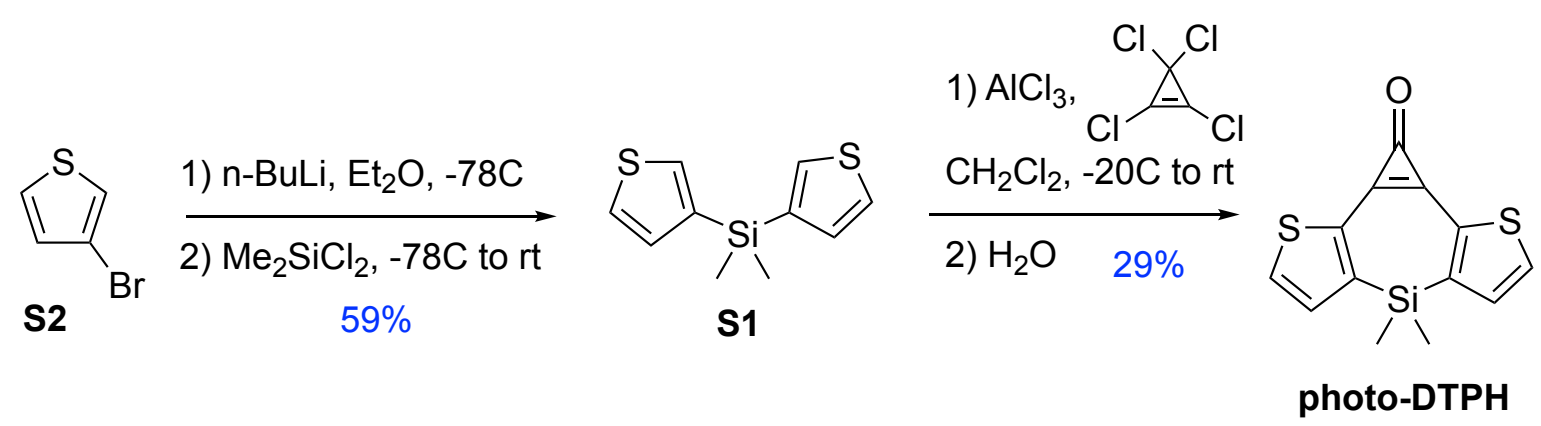

Scheme S1. Synthesis of photo-DTPH

Dimethyl[di(3-thienyl)]silane S1.

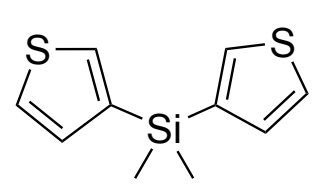

A solution of *n*-BuLi in hexanes (1.5 M, 10 mL, 15.0 mmol) was slowly added to a solution of 3-bromothiophene (937 μL, 10.0 mmol) in diethyl ether (40 mL) at –70 °C. The solution was stirred at –70 °C for 1 h then dimethyldichlorosilane (603 μL, 5.0 mmol) was slowly added and the reaction mixture was allowed to warm to room temperature. After 1 h at room temperature, the mixture was quenched with a saturated aqueous solution of ammonium chloride (30 mL). The organic layer was then extracted with diethyl ether (3×20 mL), washed with brine (20 mL), dried over $MgSO_4$ and concentrated under reduced pressure. The residue was purified by flash column chromatography on silica gel (60 g) using petroleum ether to afford pure dimethyl[di(3-thienyl)]silane **S1** (660 mg, 59%) as a colorless oil which solidified at –20 °C: ^{1}H NMR (300 MHz, $CDCl_3$) δ 0.55 (s, 6H), 7.20 (dd, *J* = 4.7, 1.1 Hz, 2H), 7.41 (dd, *J* = 4.7, 2.6 Hz, 2H), 7.47 (dd, *J* = 2.6, 1.1 Hz, 2H); ^{13}C NMR (75 MHz, $CDCl_3$) δ -1.0 (2×CH_3), 126.0 (2×CH), 131.9 (2×CH), 132.9 (2×CH), 139.2 (2×C); ^{29}Si NMR (60 MHz, $CDCl_3$) δ -16.82 (s); HRMS (m/z): [$M^{+\bullet}$] calcd for $C_{10}H_{12}S_2Si$, 224.0150; found 224.0153.

4,4-Dimethyl-cyclopropa[2'',3'':1,7]thieno[3',2':5,6]-4*H*-silepino[2,3-*b*]thiophen-1''-one photo-DTPH.

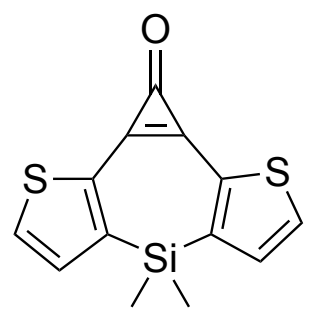

A solution of aluminum chloride (267 mg, 2.0 mmol) and tetrachlorocyclopropene (123 µL, 1.2 mmol) in dichloromethane (15 mL) was stirred at room temperature for 30 min. The reaction mixture was then cooled to –25 °C and a solution of dimethyl[di(3-thienyl)]silane **S1** (224 mg, 1.0 mmol) in dichloromethane (5 mL) was added. The reaction mixture was stirred at –25°C for 1 h, was then allowed to warm to room temperature over a period of 1 h and was stirred for an extra 1 h at room temperature. An aqueous solution of HCl (1N, 20 mL) was then added and the reaction mixture was stirred for an extra 30 min. The organic layer was then extracted with dichloromethane (3×10 mL), dried over $MgSO_4$ and concentrated under reduced pressure. The residue was then purified by flash column chromatography on silica gel (9 g) using a mixture of dichloromethane and methyl *tert*-butyl ether (98:2) to afford pure cyclopropenone **photo-DTPH** (80 mg, 29%) as an orange solid: ^{1}H NMR (300 MHz, $CDCl_3$) δ 0.55 (s, 6H), 7.35 (d, *J* = 4.9 Hz, 2H), 7.83 (d, *J* = 4.9 Hz, 2H);^{13}C NMR (75 MHz, $CDCl_3$) δ 1.6 (2×CH_3), 131.1 (2×C), 133.6 (2×CH), 135.1 (2×CH), 137.6 (2×C), 143.7 (2×C), 147.5 (C=O); ^{29}Si NMR (119 MHz, $CDCl_3$) δ –12.38 (s); HRMS (m/z): [M+H$^+$] calcd for $C_{13}H_{11}OS_2Si$, 275.0021; found 275.0018.

General Procedure for the Cycloaddition between DTPO and various 1,3-Dipoles. A solution of **photo-DTPO** (24.4 mg, 0.1 mmol) or **photo-DTPH** (27.4 mg, 0.1 mmol) and the respective 1,3-dipole (0.2 mmol) in a mixture of dichloromethane and methanol (4:1, 20 mL) was irradiated at 350 nm at room temperature for 1 h. Solvents were removed under reduced pressure, and the residue was purified by flash column chromatography on silica gel (5 g) using various mixtures of petroleum ether and methyl tert-butyl ether, affording the desired pure cycloadducts, respectively.

1-Benzyl-5,6-dihydro-dithieno[2',3':3,4:3',2':7,8]cycloocta[1,2-*d*][1,2,3]triazole 9.

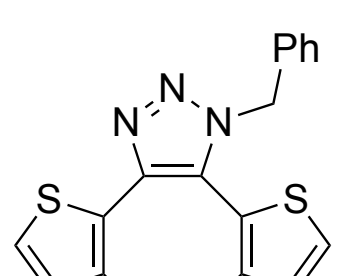

Yellow solid (29 mg, 83%): ^{1}H NMR (300 MHz, $CDCl_3$) δ 2.77–2.87 (m, 2H), 3.07–3.17 (m, 2H), 5.65 (s, 2H), 6.76 (d, *J* = 5.2 Hz, 1H), 6.90 (d, *J* = 5.1 Hz, 1H), 7.04–7.11 (m, 2H), 7.14–7.26 (m, 4H), 7.31 (d, *J* = 5.1 Hz, 1H); ^{13}C NMR (75 MHz, $CDCl_3$) δ 26.2 (CH_2), 32.7 (CH_2), 52.6 (CH_2), 121.1 (C), 125.5 (CH), 126.5 (C), 127.2 (2×CH), 127.7 (C), 128.3 (CH), 128.3 (CH), 128.9 (2×CH), 129.2 (CH), 131.1 (CH), 135.5 (C),

137.0 (C), 142.2 (C), 146.0 (C); HRMS (m/z): [M+H$^+$] calcd for $C_{13}H_{16}N_3S_2$, 350.0786; found 350.0787.

1-Benzyl-7,7-dimethyl-1,7-dihydrodithieno[2',3':6,7:3',2':2,3]silepino[4,5-d][1,2,3]triazole 10.

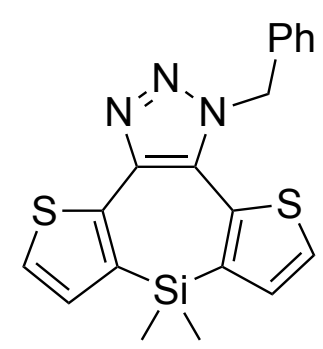

Yellow solid (32 mg, 83%): ^{1}H NMR (300 MHz, $CDCl_3$) δ 0.35 (s, 6H), 6.03 (s, 2H), 7.09–7.20 (m, 4H), 7.25–7.33 (m, 3H), 7.50 (d, J = 5.0 Hz, 1H), 7.54 (d, J = 5.0 Hz, 1H); ^{13}C NMR (75 MHz, $CDCl_3$) δ –2.8 (2×CH_3), 53.3 (CH_2), 127.0 (2×CH), 127.7 (CH), 128.3 (CH), 128.6 (CH), 128.9 (2×CH), 129.4 (C), 131.3 (CH), 132.1 (CH), 135.3 (C), 136.6 (C), 139.8 (C), 141.2 (C), 141.6 (C); missing quaternary C signal due to relaxation issue; ^{29}Si NMR (119 MHz, $CDCl_3$) δ -17.65 (s); HRMS ESI (m/z): [M+H$^+$] calcd for $C_{19}H_{18}N_3S_2Si$, 380.0711; found 380.0709.

2-Phenyl-5,6-dihydro-dithieno[2',3':3,4;3',2':7,8]cycloocta[1,2-*c*]pyrazole 15.

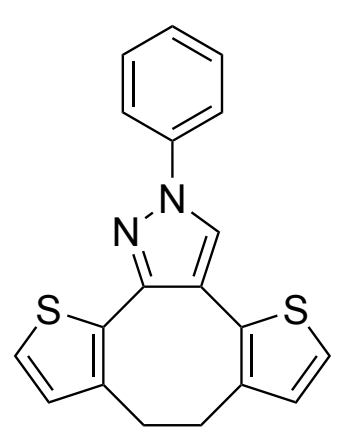

Pale yellow solid (27 mg, 82%): ^{1}H NMR (300 MHz, $CDCl_3$) δ 3.06–3.13 (m, 2H), 3.14–3.22 (m, 2H), 6.87 (d, J = 5.2 Hz, 1H), 6.90 (d, J = 5.2 Hz, 1H), 7.14 (d, J = 5.2 Hz, 1H), 7.22 (d, J = 5.2 Hz, 1H), 7.25–7.33 (m, 1H), 7.41–7.50 (m, 2H), 7.73–7.81 (m, 2H), 8.01 (s, 1H); ^{13}C NMR (75 MHz, $CDCl_3$) δ 28.6 (CH_2), 30.8 (CH_2), 116.7 (C), 119.1 (2×CH), 123.9 (CH), 125.6 (CH), 126.3 (CH), 126.8 (CH), 128.0 (C), 128.8 (C), 129.6 (2×CH), 130.4 (CH), 130.7 (CH), 139.1 (C), 139.4 (C), 139.7 (C), 146.6 (C); HRMS (m/z): [M+H$^+$] calcd for $C_{19}H_{15}N_2S_2$, 335.0677; found 335.0678.

2-(4-Methoxyphenyl)-5,6-dihydro-dithieno[2',3':3,4;3',2':7,8]cycloocta[1,2-*c*]pyrazole 16.

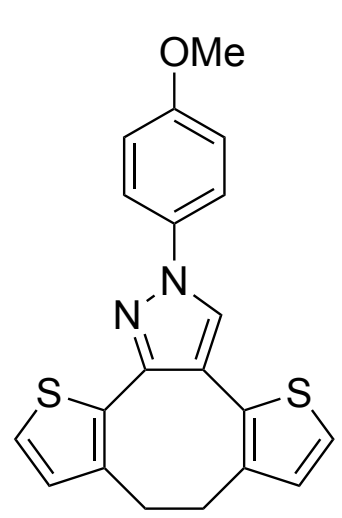

Pale yellow solid (35 mg, 96%): ^{1}H NMR (400 MHz, $CDCl_3$) δ 3.09–3.15 (m, 2H), 3.17–3.23 (m, 2H), 3.86 (s, 3H), 6.90 (d, J = 5.2 Hz, 1H), 6.93 (d, J = 5.1 Hz, 1H), 6.99 (d, J = 9.0 Hz, 2H), 7.16 (d, J = 5.1 Hz, 1H), 7.23 (d, J = 5.2 Hz, 1H), 7.69 (d, J = 9.0 Hz, 2H), 7.94 (s, 1H); ^{13}C NMR (101 MHz, $CDCl_3$) δ 28.6 (CH_2), 30.8 (CH_2), 55.7 (OCH_3), 114.7 (2×CH), 116.3 (C), 120.8 (2×CH), 123.8 (CH), 125.5 (CH), 126.4 (CH), 128.1 (C), 128.9 (C), 130.4 (CH), 130.6 (CH), 133.5 (C), 139.0 (C), 139.2 (C), 146.0 (C), 158.6 (C); HRMS (m/z): [M+H$^+$] calcd for $C_{20}H_{17}N_2OS_2$, 365.0782; found 365.0781.

2-[4-(Trifluoromethyl)phenyl]-5,6-dihydro-dithieno[2',3':3,4;3',2':7,8]cycloocta[1,2-*c*]pyrazole 17.

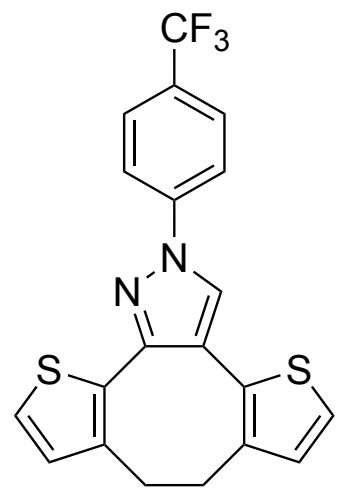

Yellow solid (35 mg, 88%): ^{1}H NMR (300 MHz, $CDCl_3$) δ 3.08–3.15 (m, 2H), 3.18–3.25 (m, 2H), 6.91 (d, *J* = 5.2 Hz, 1H), 6.94 (d, *J* = 5.2 Hz, 1H), 7.20 (d, *J* = 5.2 Hz, 1H), 7.27 (d, *J* = 5.2 Hz, 1H), 7.74 (d, *J* = 8.3 Hz, 2H), 7.92 (d, *J* = 8.3 Hz, 2H), 8.09 (s, 1H); ^{13}C NMR (75 MHz, $CDCl_3$) δ 28.5 (CH_2), 30.8 (CH_2), 117.7 (C), 118.7 (2×CH), 124.1 (q, *J* = 272.5 Hz, CF_3), 124.3 (CH), 126.0 (CH), 126.2 (CH), 126.9 (q, *J* = 3.8 Hz, 2×CH), 127.4 (C), 128.2 (C), 128.6 (q, *J* = 32.9 Hz, C), 130.4 (CH), 130.8 (CH), 139.5 (C), 139.8 (C), 142.0 (C), 147.6 (C); ^{19}F NMR (376 MHz, $CDCl_3$) δ –62.24 (s); HRMS ESI (m/z): [M+H$^+$] calcd for $C_{20}H_{14}F_3N_2S_2$, 403.0550; found 403.0551

3-Chloro-2-phenyl-5,6-dihydro-dithieno[2',3':3,4;3',2':7,8]cycloocta[1,2-*c*]pyrazole 18.

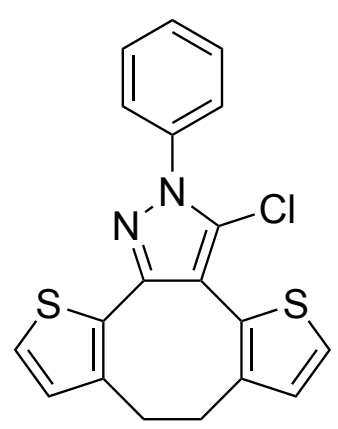

Yellow solid (33 mg, 89%): ^{1}H NMR (300 MHz, $CDCl_3$) δ 3.03–3.12 (m, 2H), 3.22–3.31 (m, 2H), 6.86 (d, *J* = 5.2 Hz, 1H), 7.00 (d, *J* = 5.1 Hz, 1H), 7.22 (d, *J* = 5.2 Hz, 1H), 7.32 (d, *J* = 5.1 Hz, 1H), 7.41–7.59 (m, 3H), 7.65–7.73 (m, 2H); ^{13}C NMR (75 MHz, $CDCl_3$) δ 26.7 (CH_2), 32.8 (CH_2), 112.9 (C), 125.4 (2×CH), 125.6 (CH), 125.8 (CH), 126.6 (C), 128.0 (C), 128.7 (CH), 128.8 (CH), 129.2 (2×CH), 131.3 (CH), 138.2 (C), 138.4 (C), 141.9 (C), 147.2 (C), missing C–Cl signal due to relaxation issue; HRMS (m/z): [M+H$^+$] calcd for $C_{19}H_{14}ClN_2S_2$, 369.0287; found 369.0291.

7,7-Dimethyl-2-phenyl-2,7-dihydrodithieno[2',3':6,7:3',2':2,3]silepino[4,5-c]pyrazole 19.

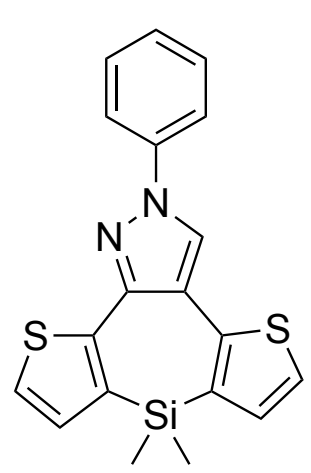

Yellow solid (30 mg, 83%): ^{1}H NMR (300 MHz, $CDCl_3$) δ 0.46 (s, 6H), 7.16 (d, *J* = 5.0 Hz, 1H), 7.20 (d, *J* = 5.0 Hz, 1H), 7.31 (d, *J* = 5.0 Hz, 1H), 7.30–7.38 (m, 1H), 7.42 (d, *J* = 5.0 Hz, 1H), 7.46–7.56 (m, 2H), 7.78–7.90 (m, 2H), 8.25 (s, 1H); ^{13}C NMR (75 MHz, $CDCl_3$) δ –1.4 (2×CH_3), 117.5 (C), 119.2 (2×CH), 124.5 (CH), 125.9 (CH), 126.5 (CH), 127.0 (CH), 129.6 (2×CH), 132.0 (CH), 132.5 (CH), 135.2 (C), 136.9 (C), 139.5 (C), 140.7 (C), 142.0 (C), 145.6 (C); ^{29}Si NMR (119 MHz, $CDCl_3$) δ –17.40 (s); HRMS ESI (m/z): [M+H$^+$] calcd for $C_{19}H_{17}N_2S_2Si$, 365.0602; found 365.0605.

7,7-Dimethyl-2-[4-(trifluoromethyl)phenyl]-2,7-dihydrodithieno[2',3':6,7:3',2':2,3]silepino[4,5-c]pyrazole 20.

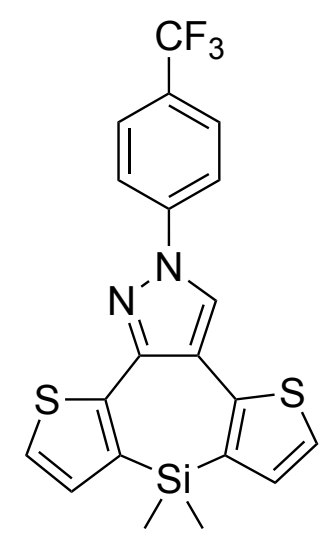

Orange solid (33 mg, 77%): ^{1}H NMR (300 MHz, $CDCl_3$) δ 0.47 (s, 6H), 7.17 (d, *J* = 5.0 Hz, 1H), 7.21 (d, *J* = 5.0 Hz, 1H), 7.33 (d, *J* = 5.0 Hz, 1H), 7.45 (d, *J* = 5.0 Hz, 1H), 7.76 (d, *J* = 8.5 Hz, 2H), 7.96 (d, *J* = 8.5 Hz, 2H), 8.29 (s, 1H); ^{13}C NMR (75 MHz, $CDCl_3$) δ –1.33 (2×CH_3), 118.4 (C), 118.9 (2×CH), 124.1 (q, *J* = 271.8 Hz, CF_3), 124.9 (CH), 125.8 (CH), 126.9 (CH), 127.0 (q, *J* = 4.2 Hz, 2×CH), 128.7 (q, *J* = 33.0 Hz, C), 132.1 (CH), 132.6 (CH), 135.7 (C), 137.6 (C), 140.1 (C), 141.5 (C), 141.9 (C), 146.5 (C); ^{19}F NMR (282 MHz, $CDCl_3$) δ –62.26 (s); ^{29}Si NMR (60 MHz, $CDCl_3$) δ –17.30 (s); HRMS ESI (m/z): [M+H$^+$] calcd for $C_{20}H_{16}F_3N_2S_2Si$, 433.0476; found 433.0482.

Stability Studies of photo-DTPO, DTPO and photo-DTPH.

The stability of the cyclopropenone-protected **photo-DTPO** and **photo-DTPH** in CH_2Cl_2:MeOH (2:1), in PBS pH 7.4 (with some DMF for solubility) and in 1 mM glutathione (GSH) in PBS pH 7.4 (with some DMF for solubility) was determined by measuring their respective absorption overtime at 21 °C.

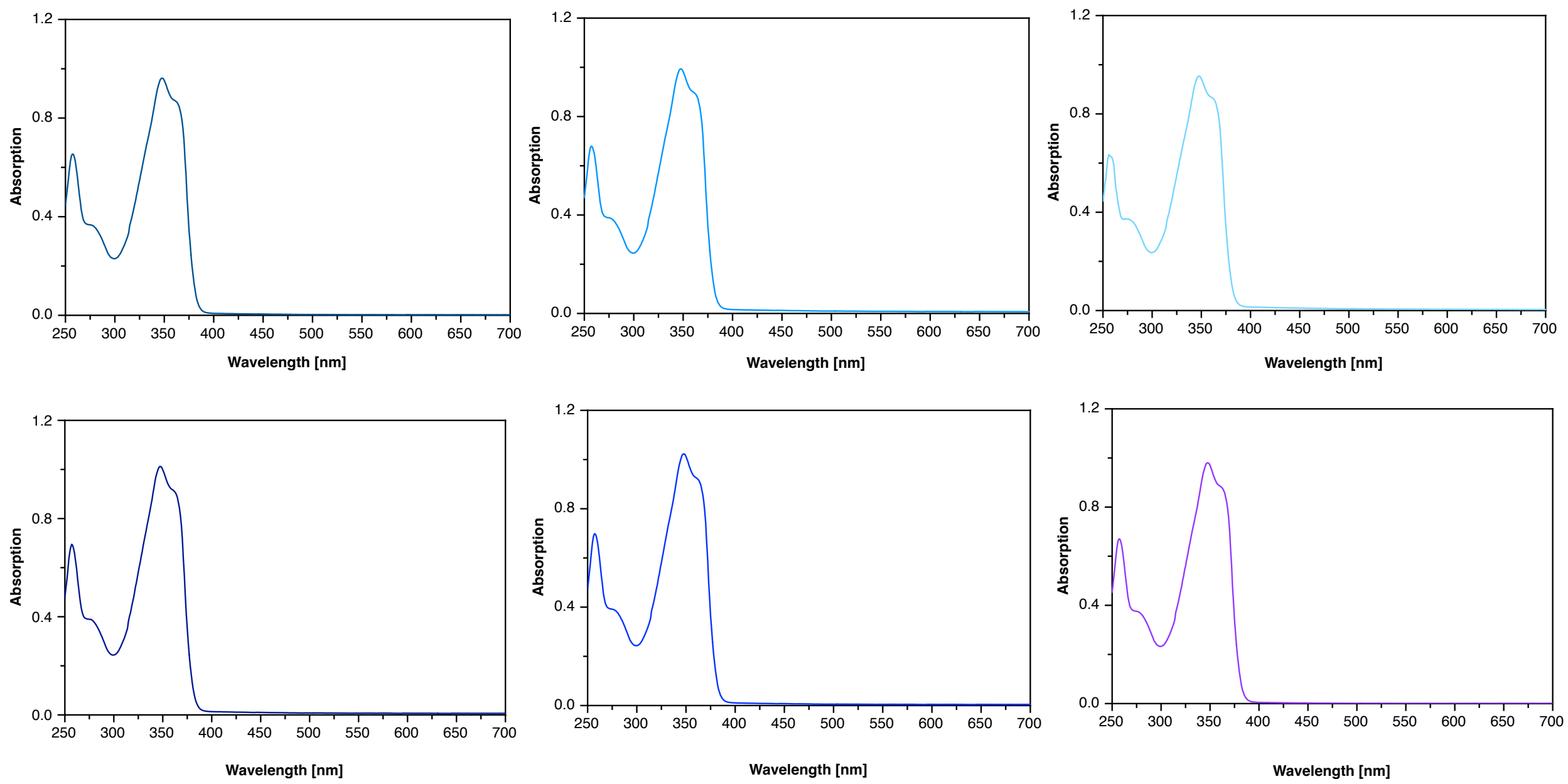

Figure S1. Absorption spectra of **photo-DTPO** (50 µM) in CH_2Cl_2:MeOH (2:1) at 0, 3 h, 6 h, 9 h, 24 h, 48 h.

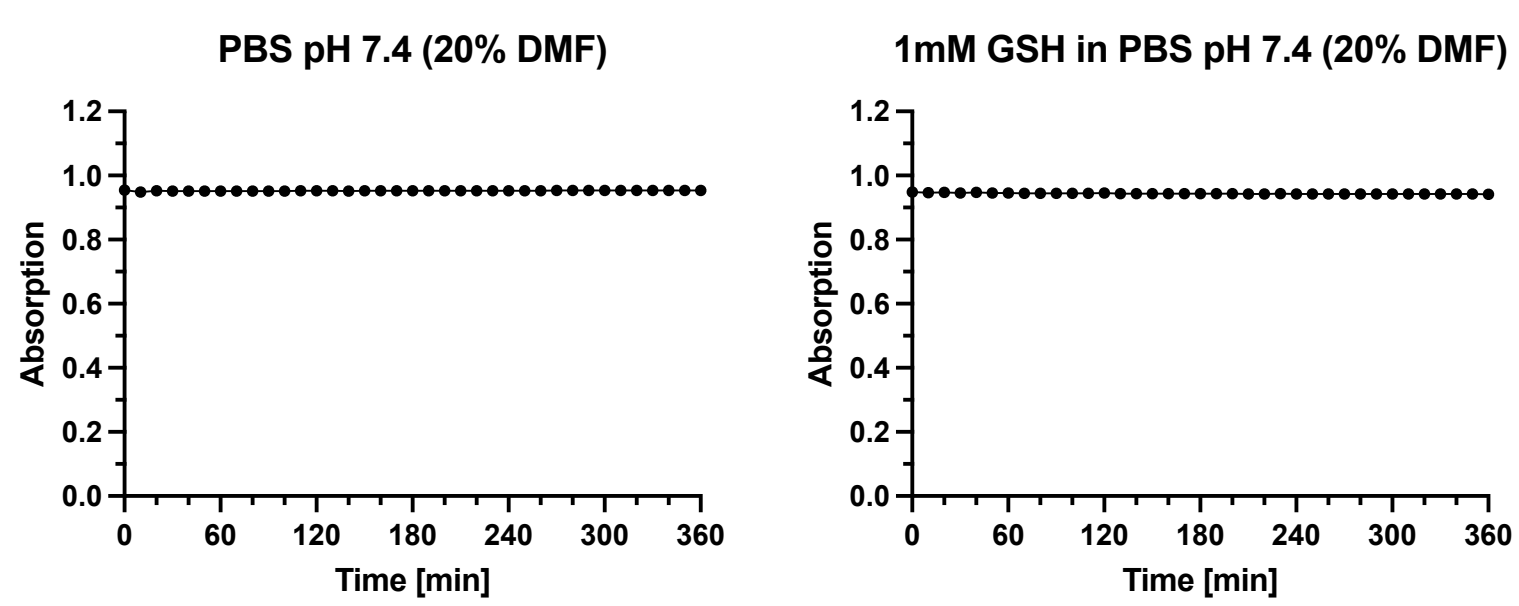

Figure S2. Absorption measurements of **photo-DTPO** (50 µM) in PBS pH 7.4 (with 20% DMF) and in 1 mM glutathione in PBS pH 7.4 (with 20% DMF) at λ_{abs} = 350 nm over 6 h.

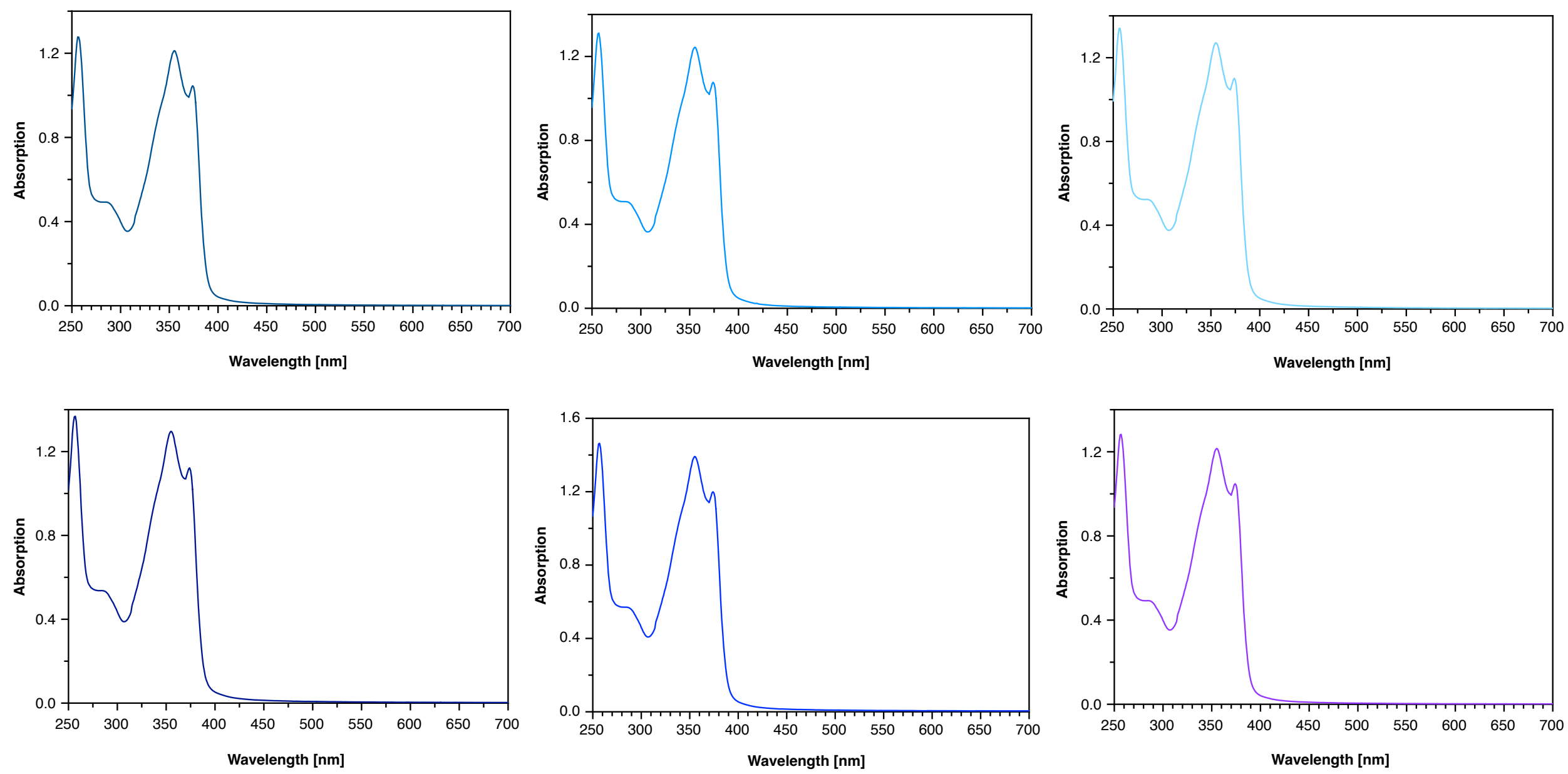

Figure S3. Absorption spectra of **photo-DTPH** (50 µM) in CH_2Cl_2:MeOH (2:1) at 0, 3 h, 6 h, 9 h, 24 h, 48 h.

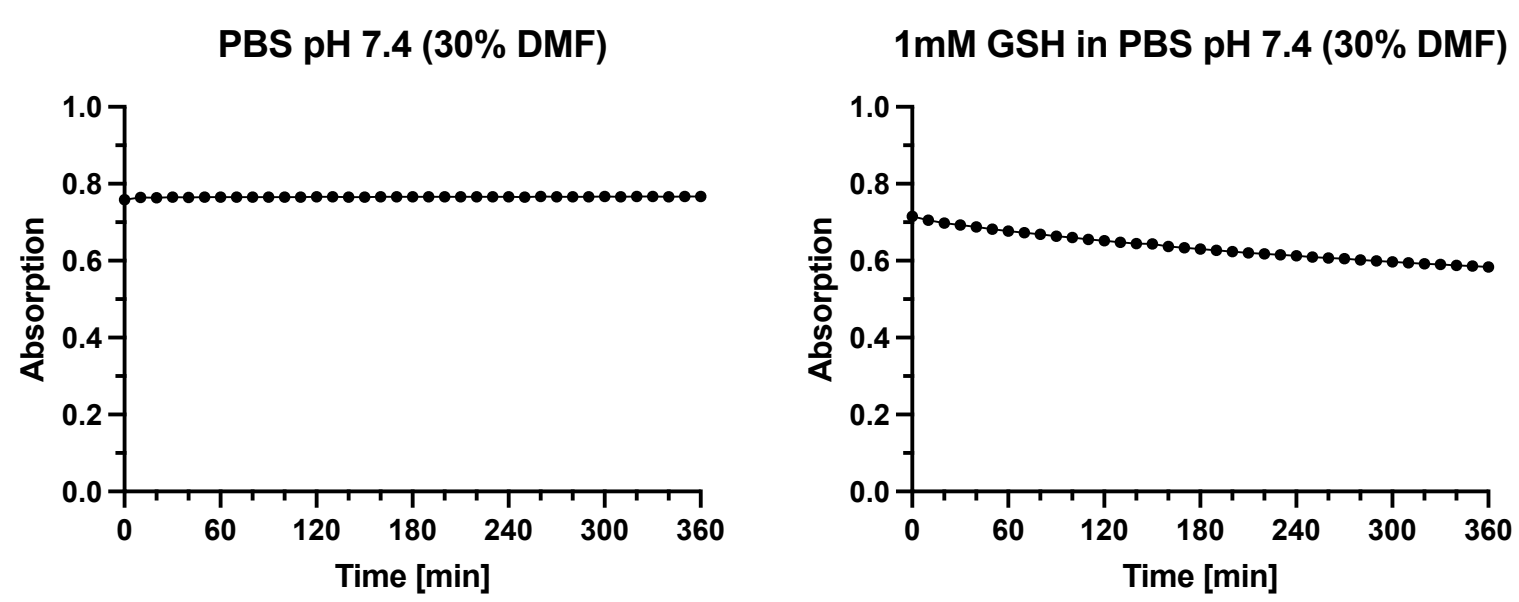

Figure S4. Absorption measurements of **photo-DTPH** (50 µM) in PBS pH 7.4 (with 30% DMF) and in 1 mM glutathione in PBS pH 7.4 (with 30% DMF) at λ_{abs} = 355 nm over 6 h.

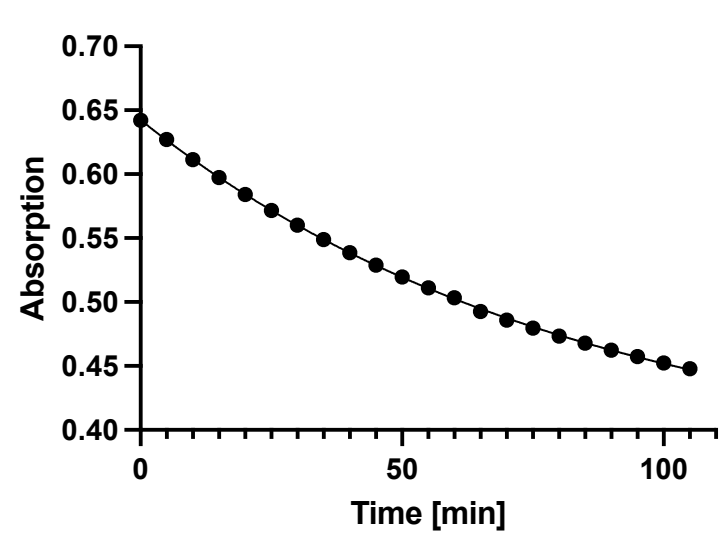

Figure S5. Absorption measurements of **DTPO** (50 µM) in CH_2Cl_2:MeOH (2:1) at λ_{abs} = 330 nm over 105 min – $t_{1/2}$ = 55 min

Absorption and Fluorescence Spectra

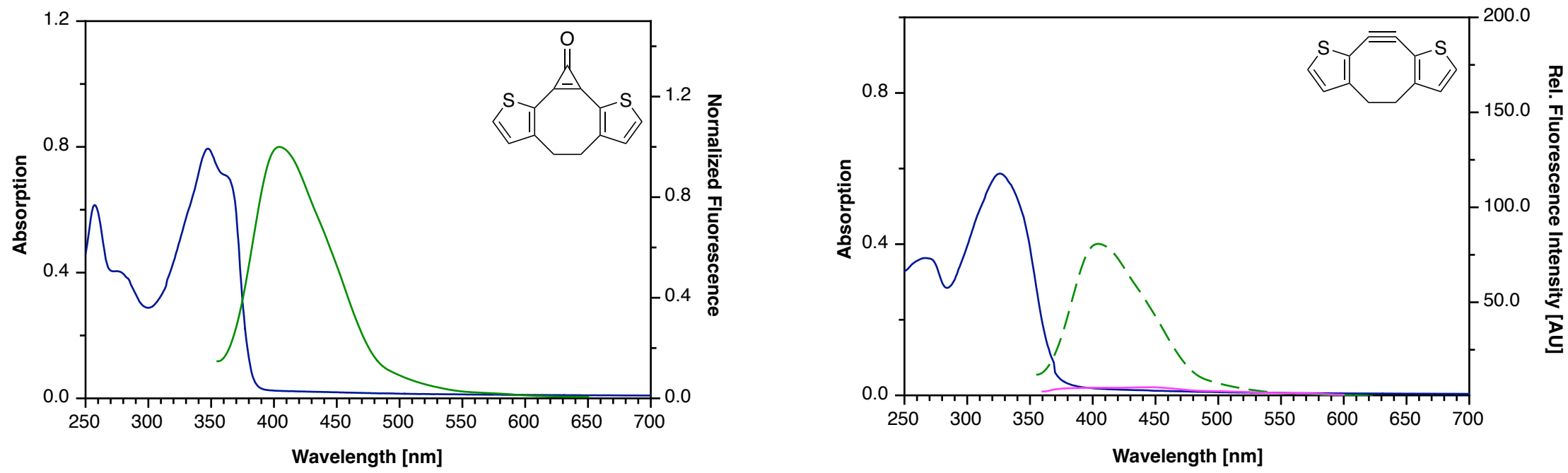

Figure S6. Absorption (blue traces (50 μM in CH_2Cl_2:MeOH (2:1) at 21 °C)) and fluorescence emission spectra (solid and dashed green trace, λ_{exc}=350 nm) of **photo-DTPO** and deprotected alkyne **DTPO** (solid pink trace, λ_{exc}=325 nm).

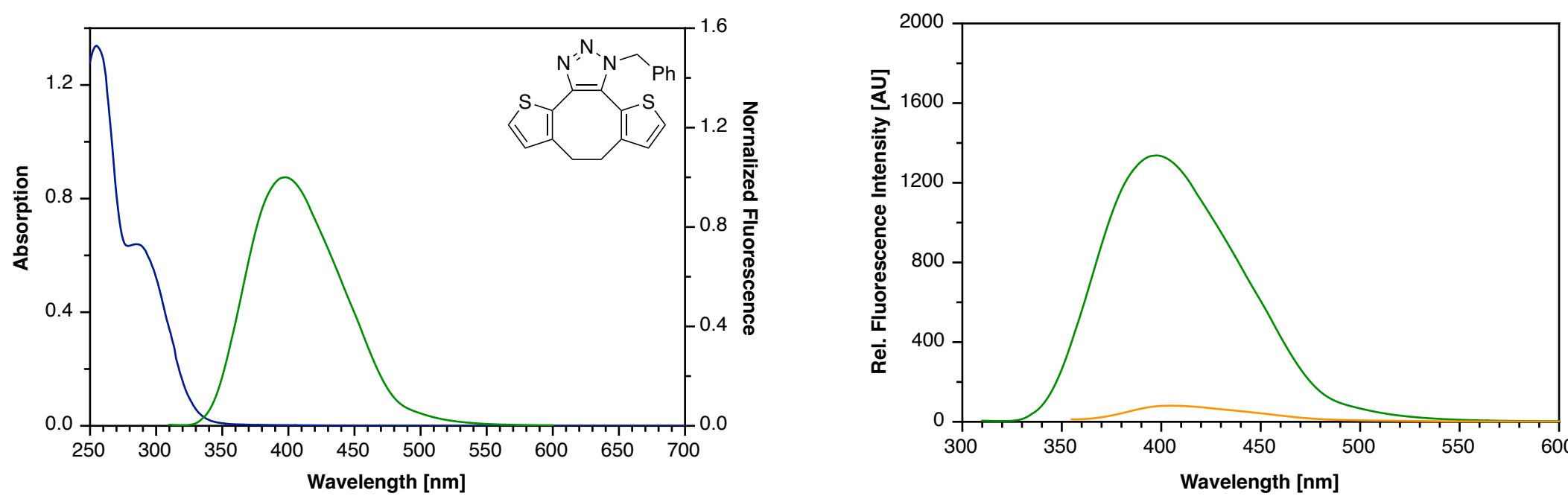

Figure S7. Absorption (blue trace (50 μM in CH_2Cl_2:MeOH (2:1) at 21 °C)) and fluorescence emission spectra (solid green traces, λ_{exc}=285 nm) of triazole **9** and **photo-DTPO** (solid orange trace, λ_{exc}=350 nm).

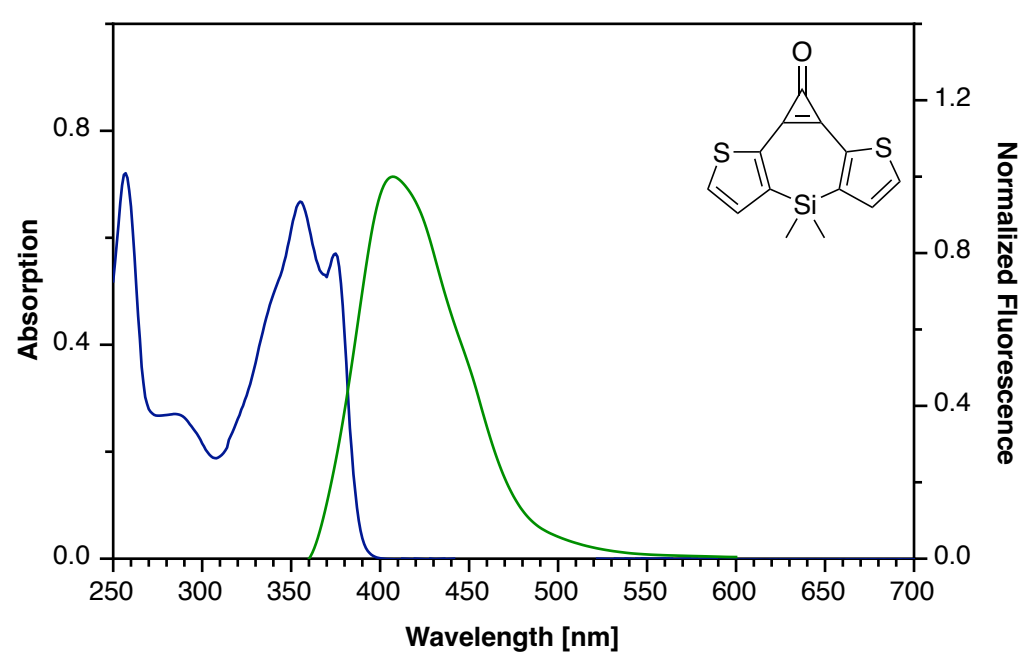

Figure S8. Absorption (blue trace (50 μM in CH_2Cl_2:MeOH (2:1) at 21 °C)) and fluorescence emission spectra (solid green trace, λ_{exc}=355 nm) of **photo-DTPH**.

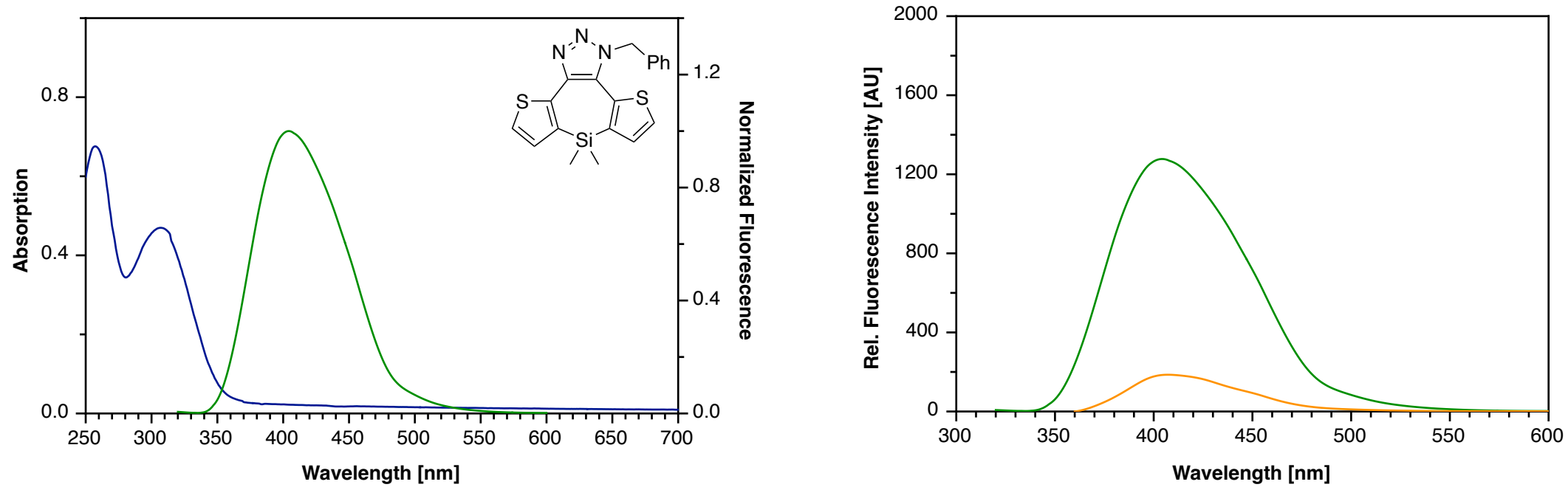

Figure S9. Absorption (blue trace (50 µM in CH_2Cl_2:MeOH (2:1) at 21 °C)) and fluorescence emission spectra (solid green traces, λ_{exc}=310 nm) of triazole **10** and **photo-DTPH** (solid orange trace, λ_{exc}=355 nm).

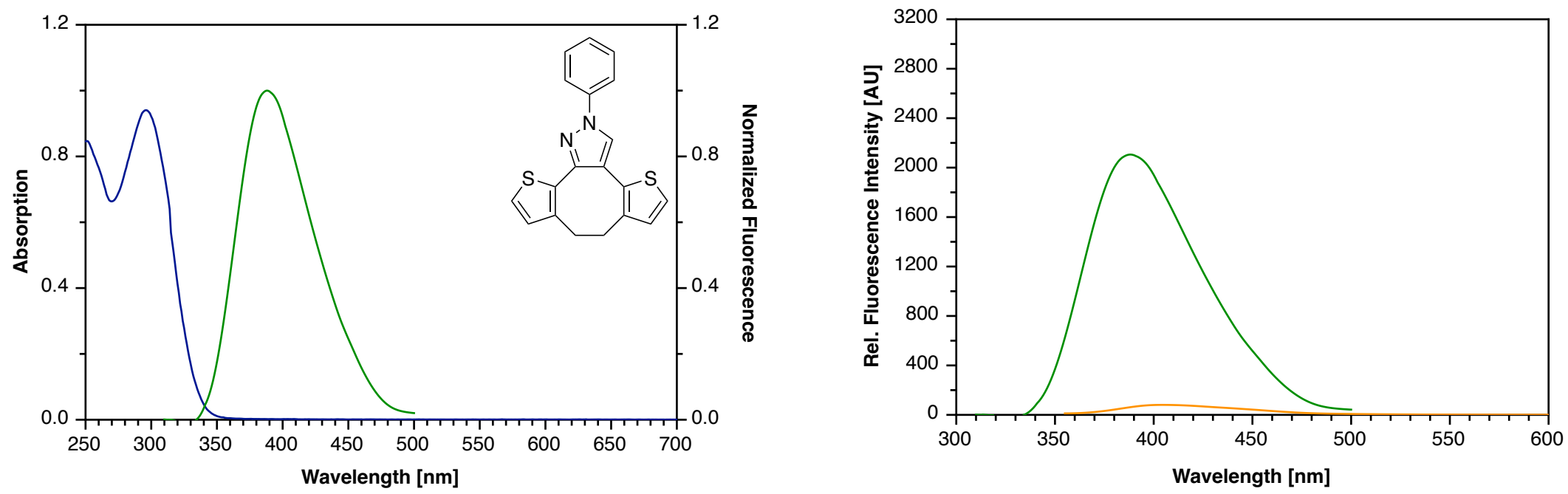

Figure S10. Absorption (blue trace (50 µM in CH_2Cl_2:MeOH (2:1) at 21 °C)) and fluorescence emission spectra (solid green traces, λ_{exc}=295 nm) of pyrazole **15** and **photo-DTPO** (solid orange trace, λ_{exc}=350 nm).

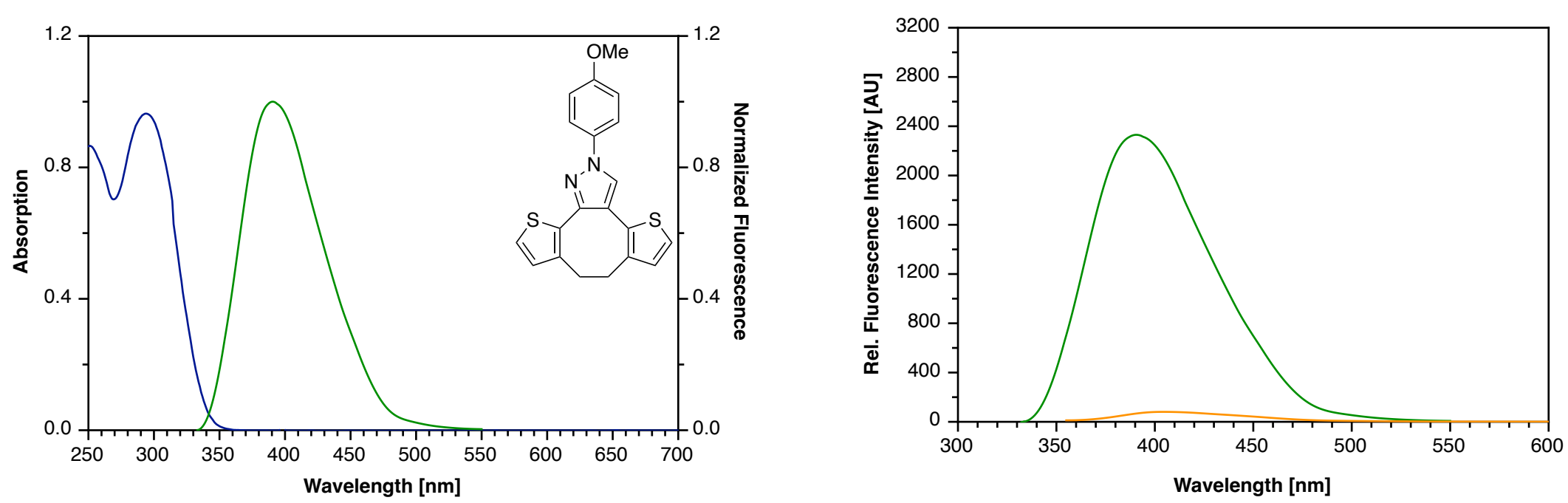

Figure S11. Absorption (blue trace (50 µM in CH_2Cl_2:MeOH (2:1) at 21 °C)) and fluorescence emission spectra (solid green traces, λ_{exc}=295 nm) of pyrazole **16** and **photo-DTPO** (solid orange trace, λ_{exc}=350 nm).

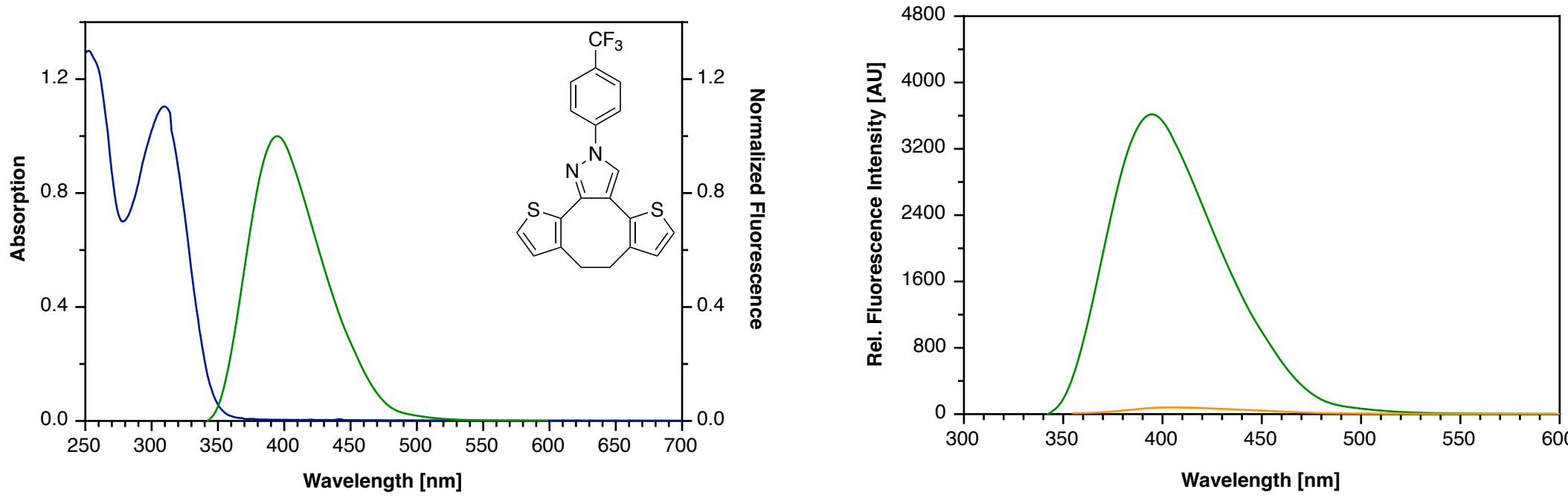

Figure S12. Absorption (blue trace (50 µM in CH_2Cl_2:MeOH (2:1) at 21 °C)) and fluorescence emission spectra (solid green traces, λ_{exc}=305 nm) of pyrazole **17** and **photo-DTPO** (solid orange trace, λ_{exc}=350 nm).

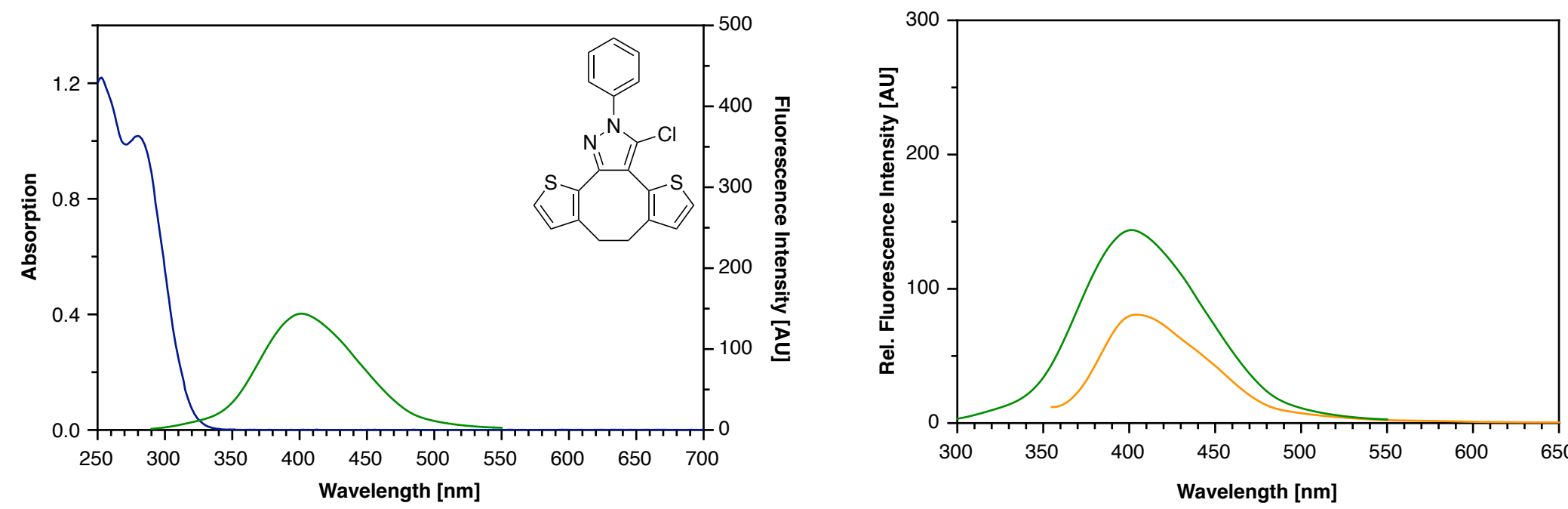

Figure S13. Absorption (blue trace (50 µM in CH_2Cl_2:MeOH (2:1) at 21 °C)) and fluorescence emission spectra (solid green traces, λ_{exc}=280 nm) of pyrazole **18** and **photo-DTPO** (solid orange trace, λ_{exc}=350 nm).

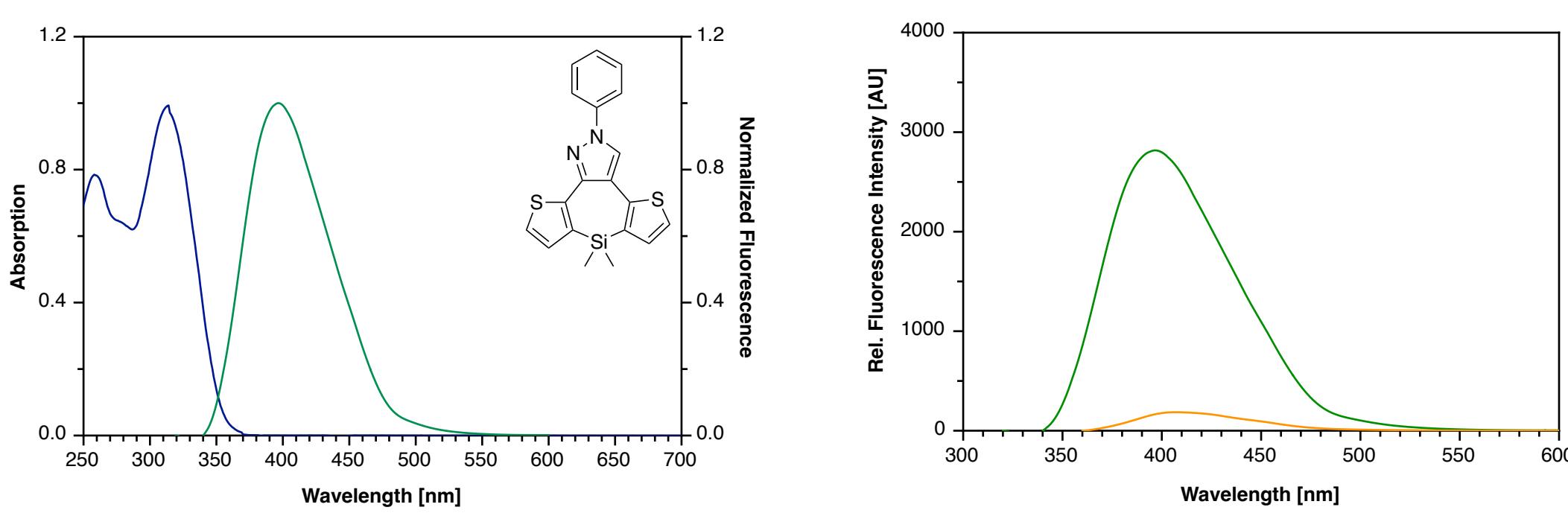

Figure S14. Absorption (blue trace (50 µM in CH_2Cl_2:MeOH (2:1) at 21 °C)) and fluorescence emission spectra (solid green traces, λ_{exc}=315 nm) of pyrazole **19** and **photo-DTPH** (solid orange trace, λ_{exc}=355 nm).

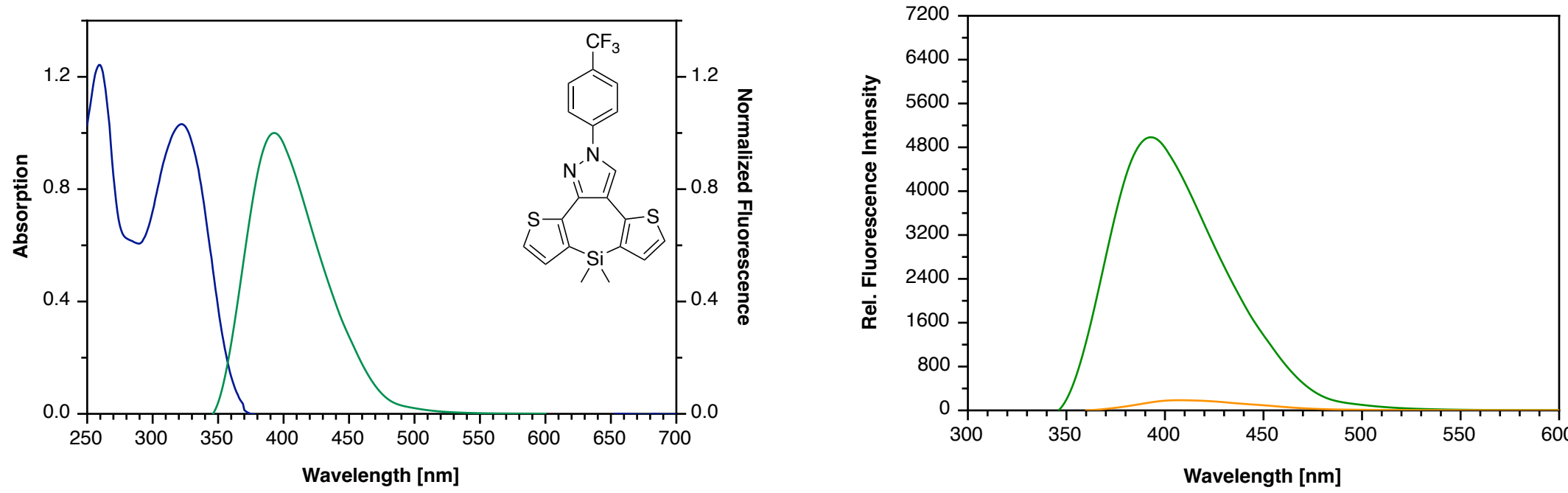

Figure S15. Absorption (blue trace (50 µM in CH_2Cl_2:MeOH (2:1) at 21 °C)) and fluorescence emission spectra (solid green traces, λ_{exc}=320 nm) of pyrazole **20** and **photo-DTPH** (solid orange trace, λ_{exc}=355 nm).

Stability Studies of triazole 9 and 10 as well as pyrazoles 15 and 19.

The stability of the triazoles **9** and **10** as well as the pyrazoles **15** and **19** in PBS pH 7.4 (with some DMF for solubility) and in 1 mM glutathione (GSH) in PBS pH 7.4 (with some DMF for solubility) was determined by measuring their respective fluorescence overtime at 21 °C.

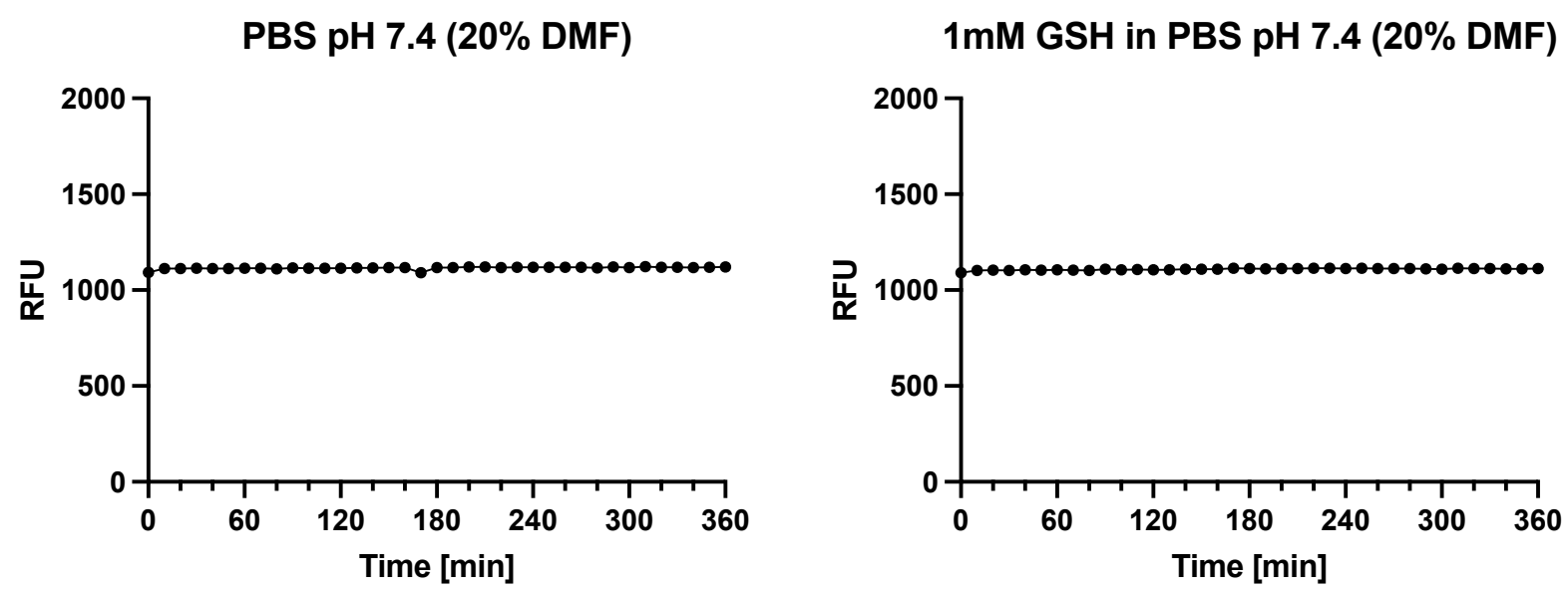

Figure S16. Fluorescence measurements of triazole **9** (50 μM) in PBS pH 7.4 (with 20% DMF) and in 1 mM GSH in PBS pH 7.4 (with 20% DMF) at λ_{exc} = 285 nm / λ_{em} = 400 nm over 6 h.

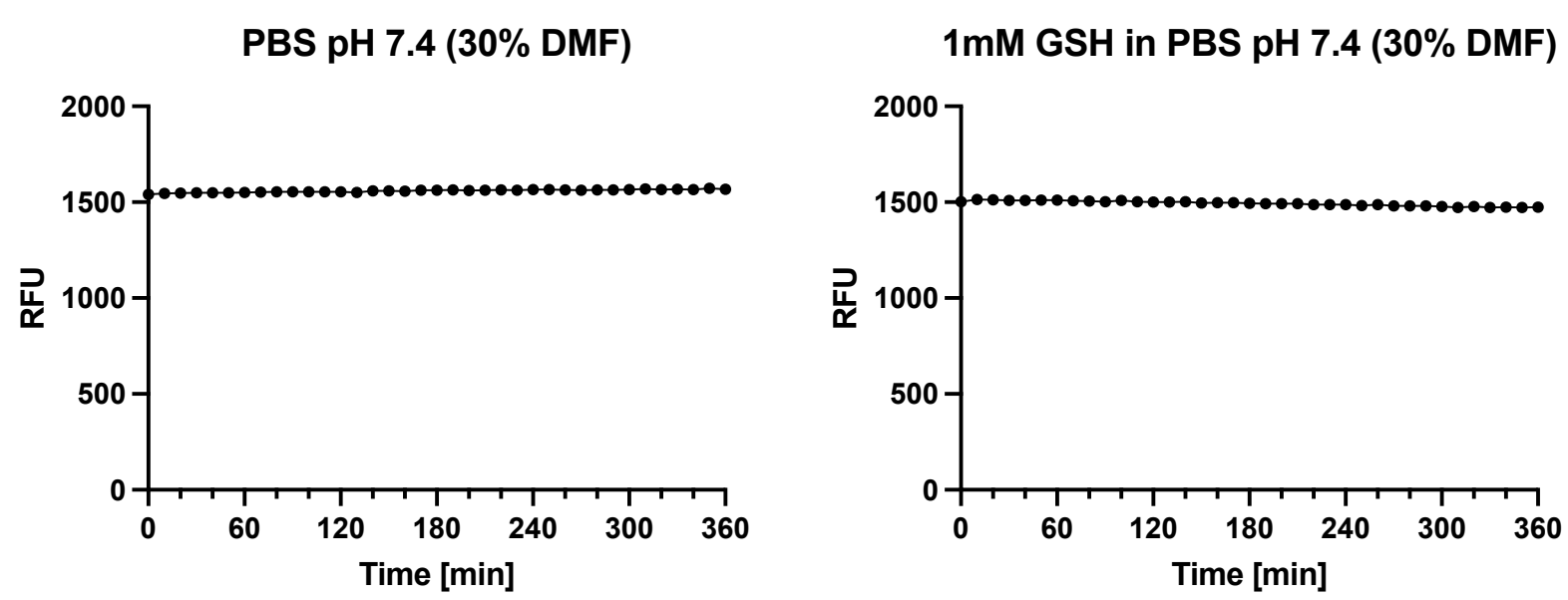

Figure S17. Fluorescence measurements of triazole **10** (50 μM) in PBS pH 7.4 (with 30% DMF) and in 1 mM GSH in PBS pH 7.4 (with 30% DMF) at λ_{exc} = 310 nm / λ_{em} = 405 nm over 6 h.

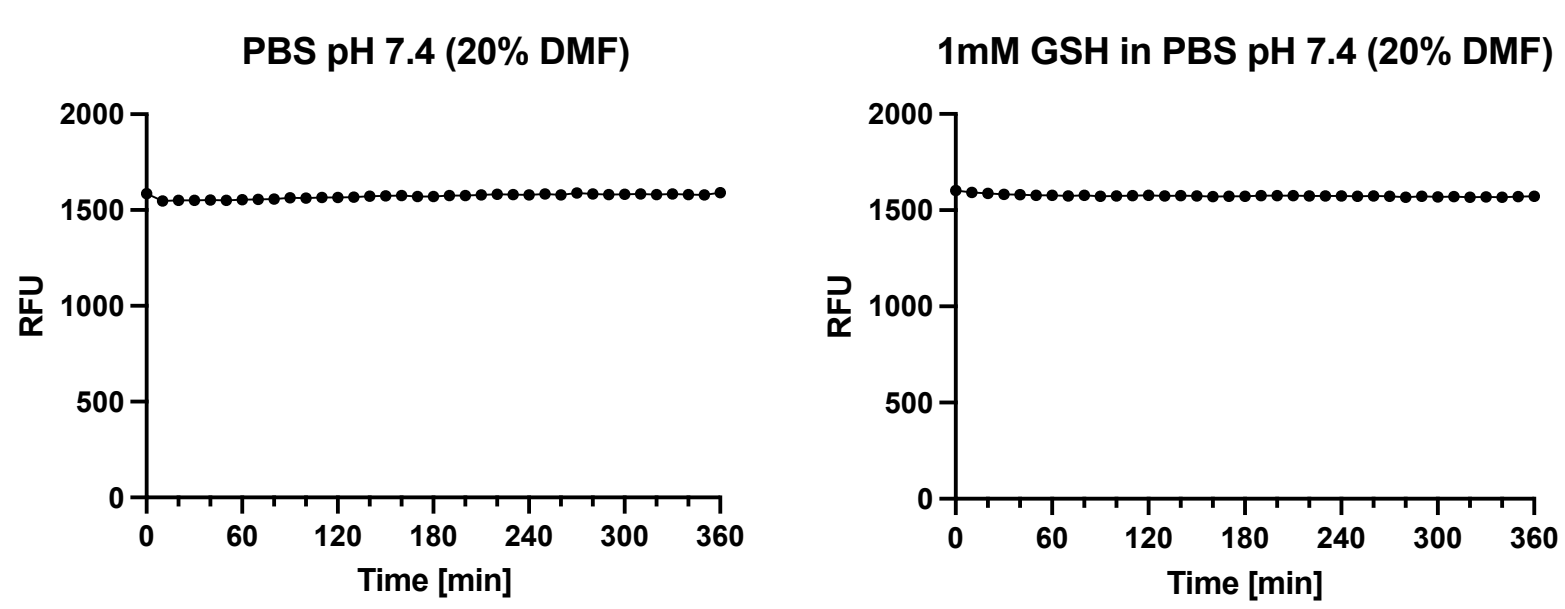

Figure S18. Fluorescence measurements of pyrazole **15** (50 μM) in PBS pH 7.4 (with 20% DMF) and in 1 mM GSH in PBS pH 7.4 (with 20% DMF) at λ_{exc} = 295 nm / λ_{em} = 390 nm over 6 h.

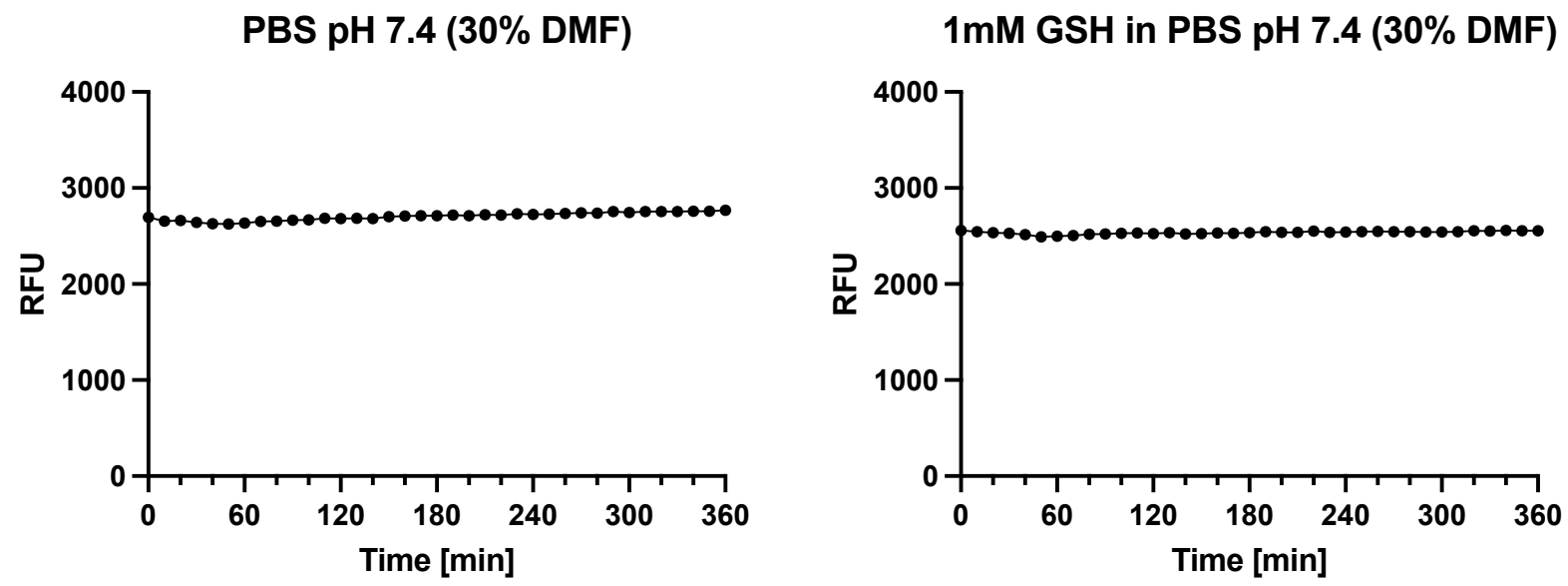

Figure S19. Fluorescence measurements of pyrazole **19** (50 μM) in PBS pH 7.4 (with 30% DMF) and in 1 mM GSH in PBS pH 7.4 (with 30% DMF) at λ_{exc} = 315 nm / λ_{em} = 395 nm over 6 h.

Kinetic Measurements

The rate measurements of cycloaddition of cyclopropenone-protected cyclooctyne **photo-DTPO** with benzyl azide or sydnones **11-13** were conducted by using fluorescence spectroscopy (SpectraMax M2E Reader from molecular devices using a cuvette with 1 cm path length) at 21 °C. A stock solution of **photo-DTPO** (100 µM) in CH_2Cl_2:MeOH (2:1) or in PBS pH 7.4 (with 20% DMF) was first irradiated at 350 nm to fully generate the corresponding cyclooctyne. A stock solution of benzyl azide (50 mM) or sydnones **11-13** (50 mM) in CH_2Cl_2:MeOH (2:1) or in PBS pH 7.4 (with 20% DMF) was then added to reach final concentrations in cyclooctyne of 5 µM, 12.5 µM, 25 µM or 50 µM (depending on the experiment) and in 1,3-dipoles ranging from 10 µM to 5 mM. The reactions were monitored by following the increase of fluorescence overtime. Formation of the cycloadduct was fitted to a single exponential equation to obtained the corresponding observed rate constants k'. The second-order rate constants were obtained by linear fitting of the various k' vs their corresponding 1,3-dipole concentrations. Experiments were performed in triplicates (Figures S20-25).

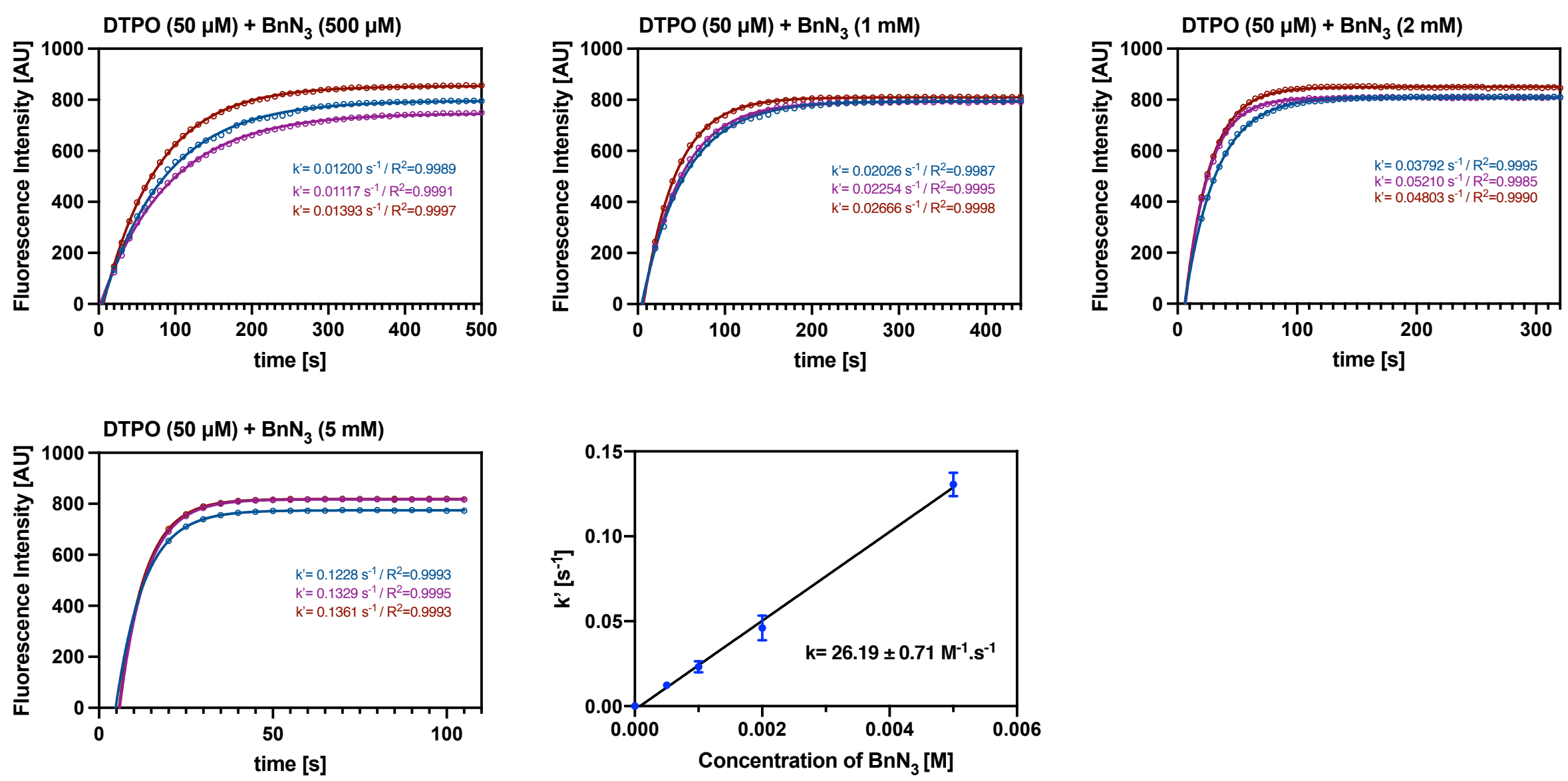

Figure S20. Kinetics measurement plots of the reaction between **DTPO** (50 µM) and benzyl azide (500 µM – 5 mM) – Fluorescence recorded at λ_{exc} = 300 nm / λ_{em} = 390 nm. Error bars are indicative of the standard deviation of three individual experiments.

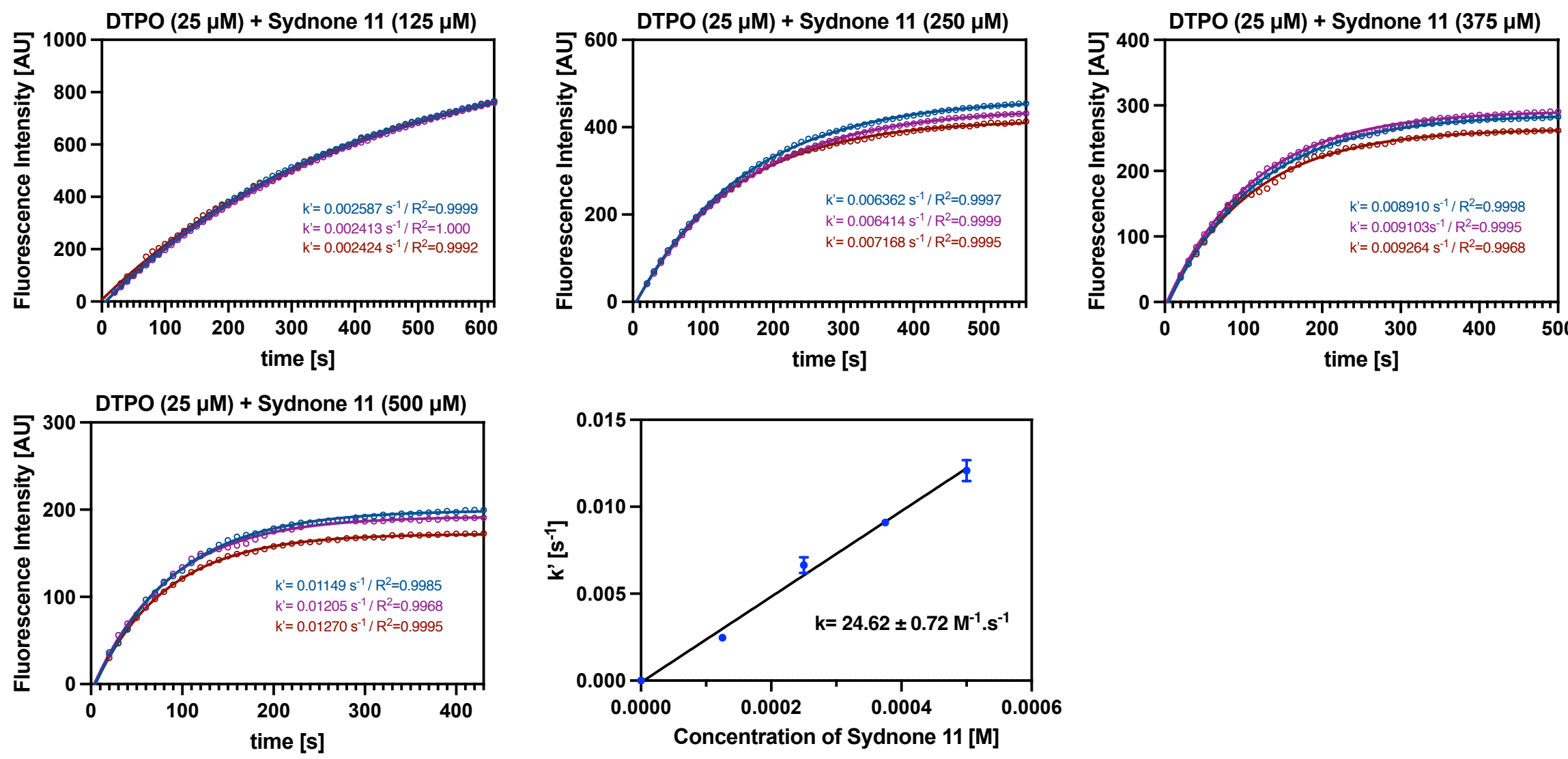

Figure S21. Kinetics measurement plots of the reaction between **DTPO** (25 μM) and sydnone **11** (125 μM – 500 μM) – Fluorescence recorded at λ_{exc} = 300 nm / λ_{em} = 385 nm. Error bars are indicative of the standard deviation of three individual experiments.

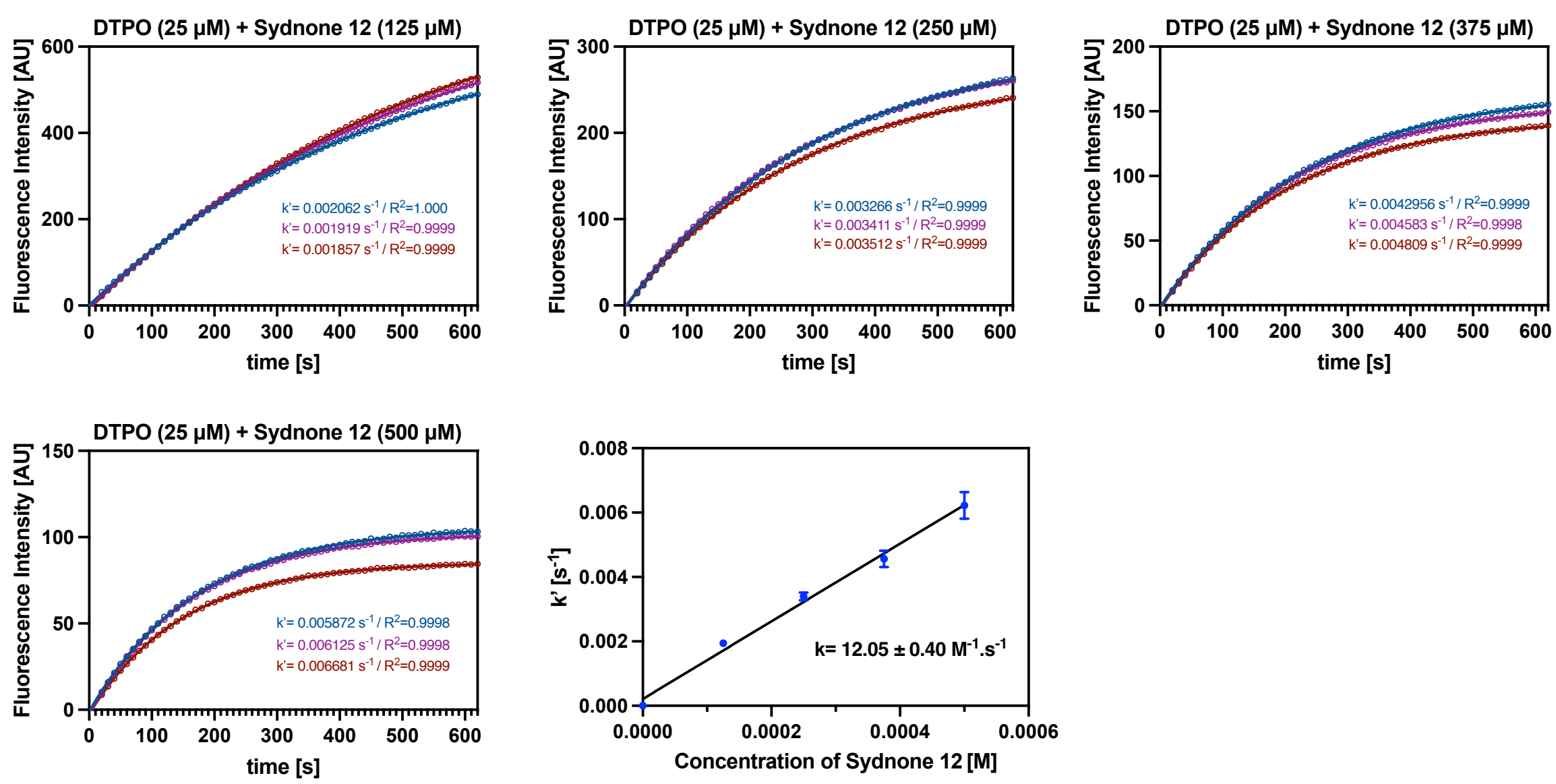

Figure S22. Kinetics measurement plots of the reaction between **DTPO** (25 μM) and sydnone **12** (125 μM – 500 μM) – Fluorescence recorded at λ_{exc} = 290 nm / λ_{em} = 390 nm. Error bars are indicative of the standard deviation of three individual experiments.

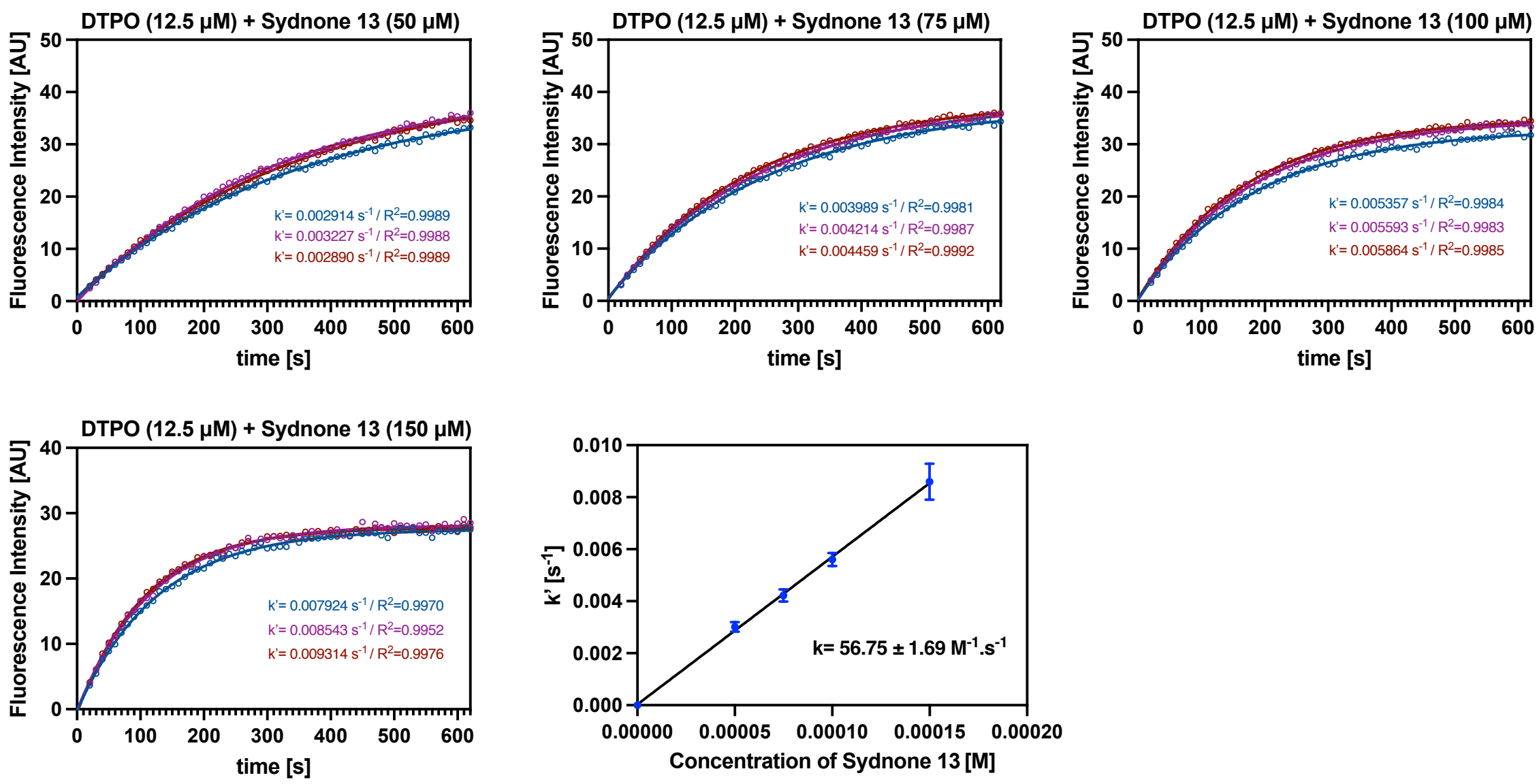

Figure S23. Kinetics measurement plots of the reaction between **DTPO** (12.5 µM) and sydnone **13** (50 µM – 150 µM) – Fluorescence recorded at λ_{exc} = 320 nm / λ_{em} = 390 nm. Error bars are indicative of the standard deviation of three individual experiments.

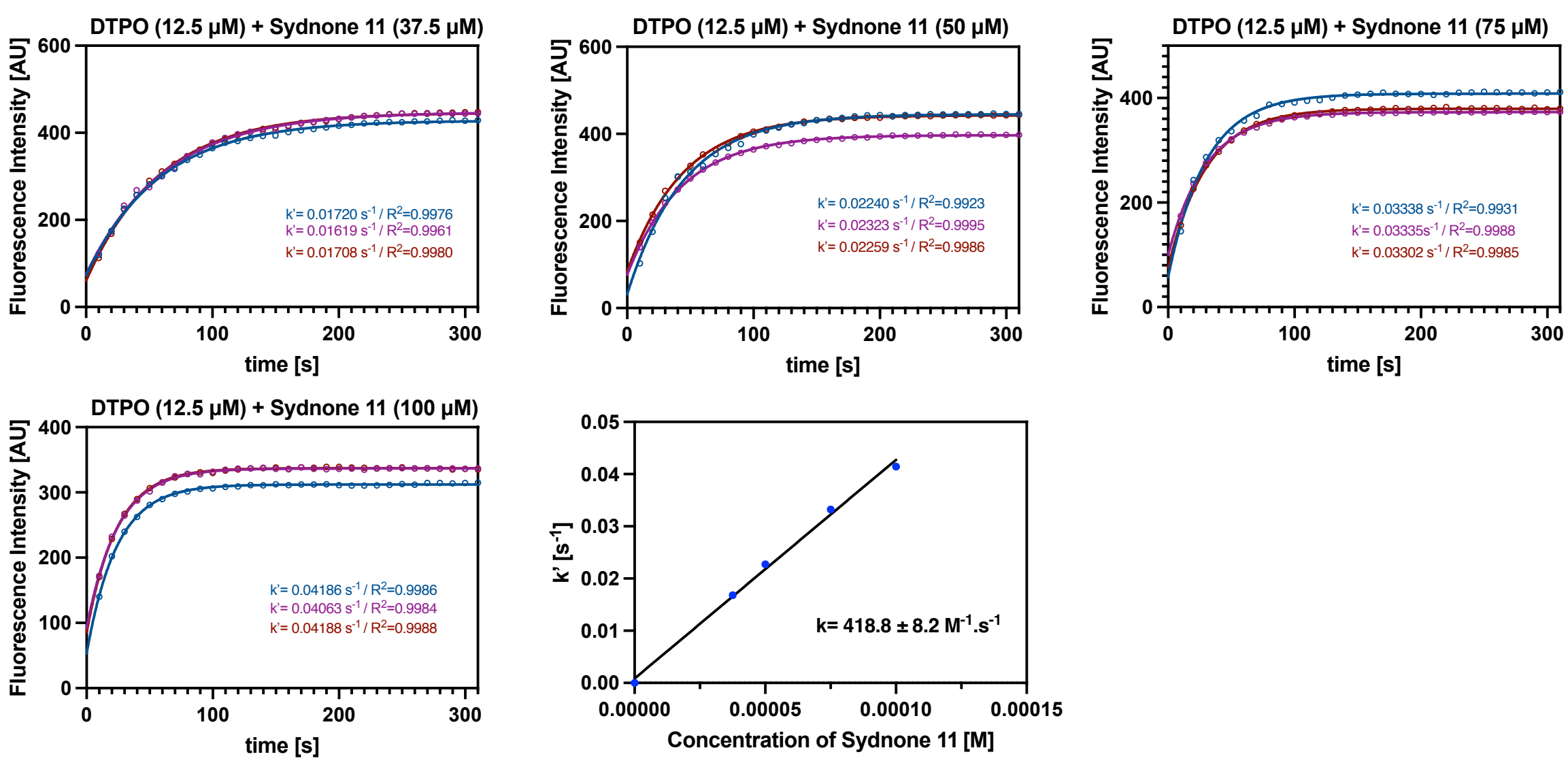

Figure S24. Kinetics measurement plots of the reaction between **DTPO** (12.5 µM) and sydnone **11** (37.5 µM – 100 µM) in PBS pH 7.4 (20% DMF) – Fluorescence recorded at λ_{exc} = 295 nm / λ_{em} = 390 nm. Error bars are indicative of the standard deviation of three individual experiments.

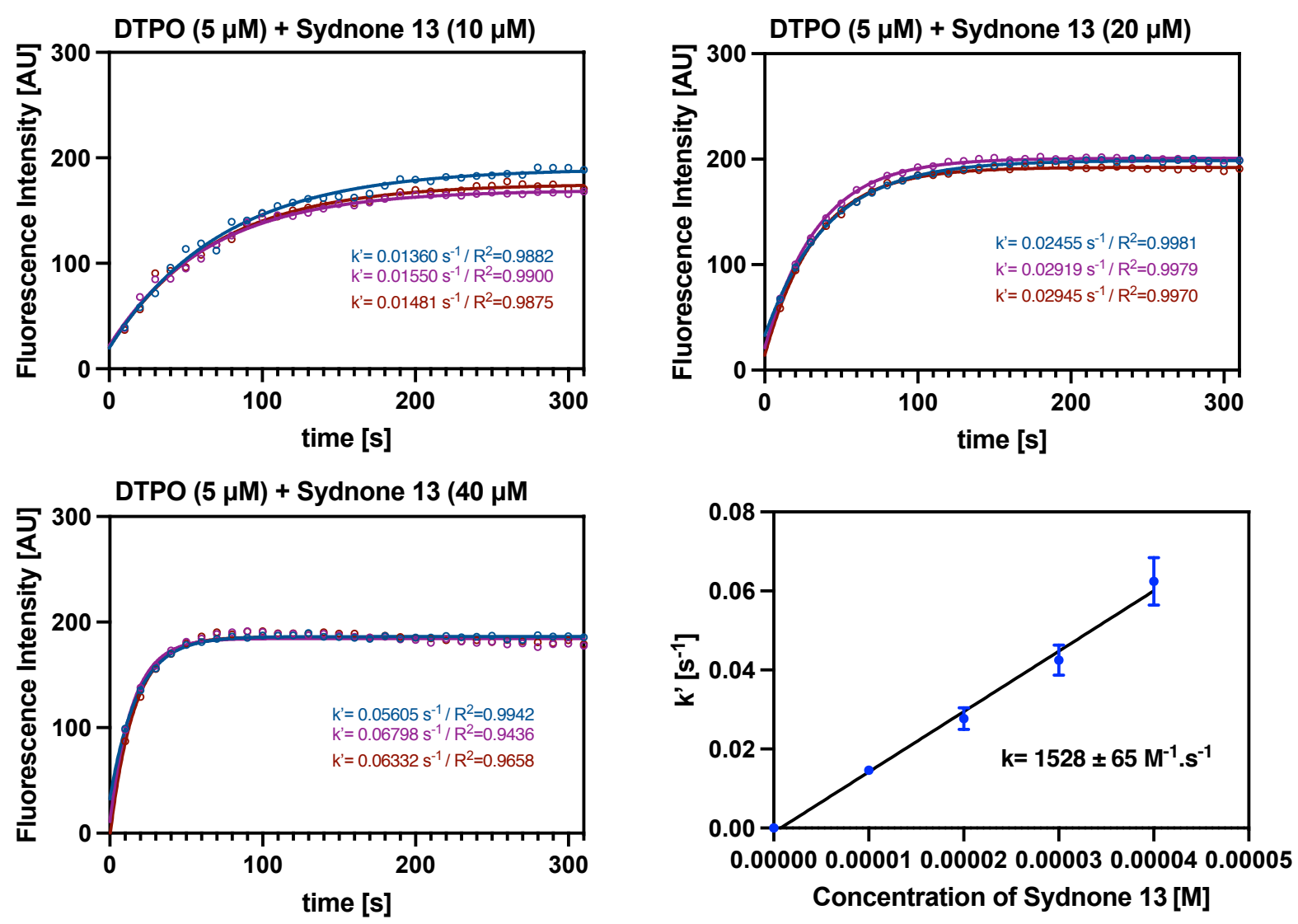

Figure S25. Kinetics measurement plots of the reaction between **DTPO** (5 µM) and sydnone **13** (10 µM – 40 µM) in PBS pH 7.4 (20% DMF) – Fluorescence recorded at λ_{exc} = 320 nm / λ_{em} = 390 nm. Error bars are indicative of the standard deviation of three individual experiments.

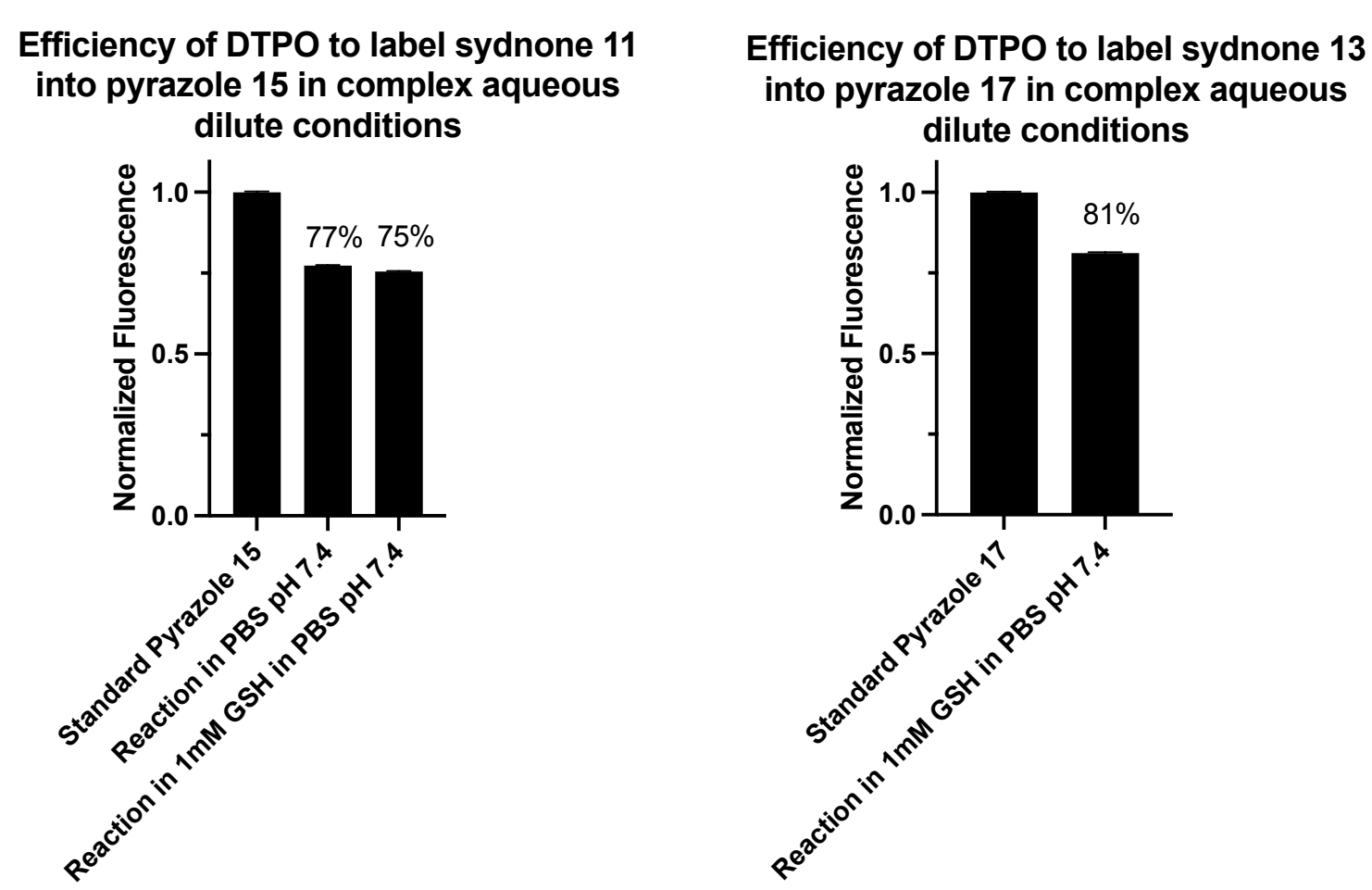

Figure S26. Fluorescence measurements (λ_{exc} = 295 nm / λ_{em} = 390 nm) of pyrazole **15** (20 µM) or pyrazole **17** (20 µM) preformed, or generated in situ from reacting photo-DTPO (20 µM) with sydnone **11** (50 µM) or sydnone **13** (50 µM), respectively, for 5 min at room temperature under irradiation (350 nm) in PBS pH 7.4 (with 20% DMF for solubility), or in 1 mM glutathione in PBS pH 7.4 (with 20% DMF for solubility).

DFT Calculations

Computational Details

All calculations were carried out by using the Gaussian 16 Rev. C.01.[1] The geometry optimizations and frequency calculations were performed at the wb97xd/6-31+g(d) level[2-3] of theory with employing the conductor-like polarizable continuum model[4] (CPCM) and the experimentally used solvent of methanol. Vibrational analysis is verified the optimized structures as either energy minima or transition states on the PES. Subsequent single point energies were computed at the wb97xd/6-311++g(d,p)[5] (CPCM-methanol) level. Intrinsic reaction coordinate (IRC) calculations were performed to verify that the saddle points found were true transition states connecting the reactants and the products.[6-7] All thermodynamic quantities (1 mol/L, 298.15 K) were computed in the GoodVibes code[8] with quasiharmonic corrections.

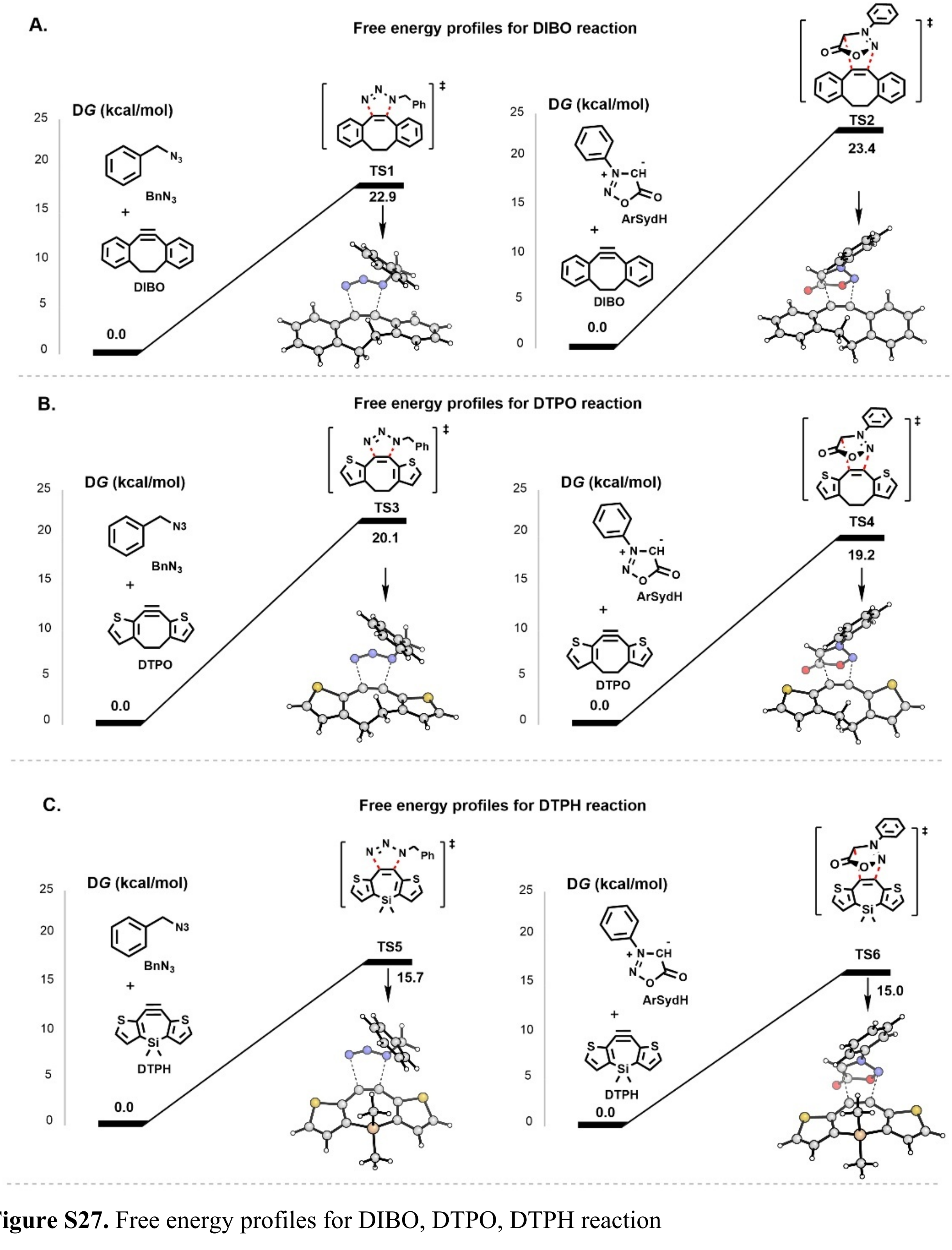

Figure S27. Free energy profiles for DIBO, DTPO, DTPH reaction

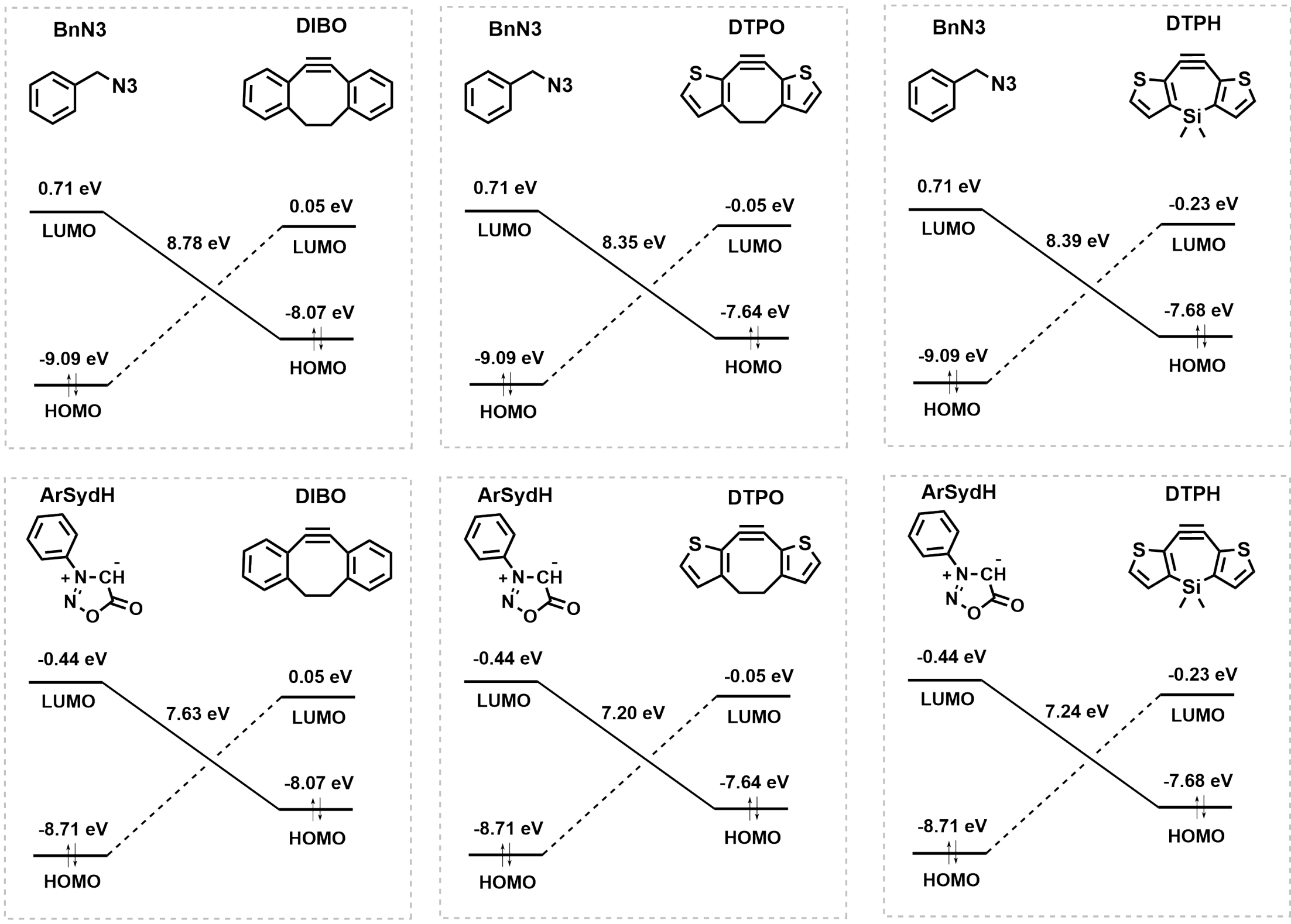

Figure S28. Frontier Molecular Orbitals (FMOs) of DIBO, DTPO, DTPH Reaction

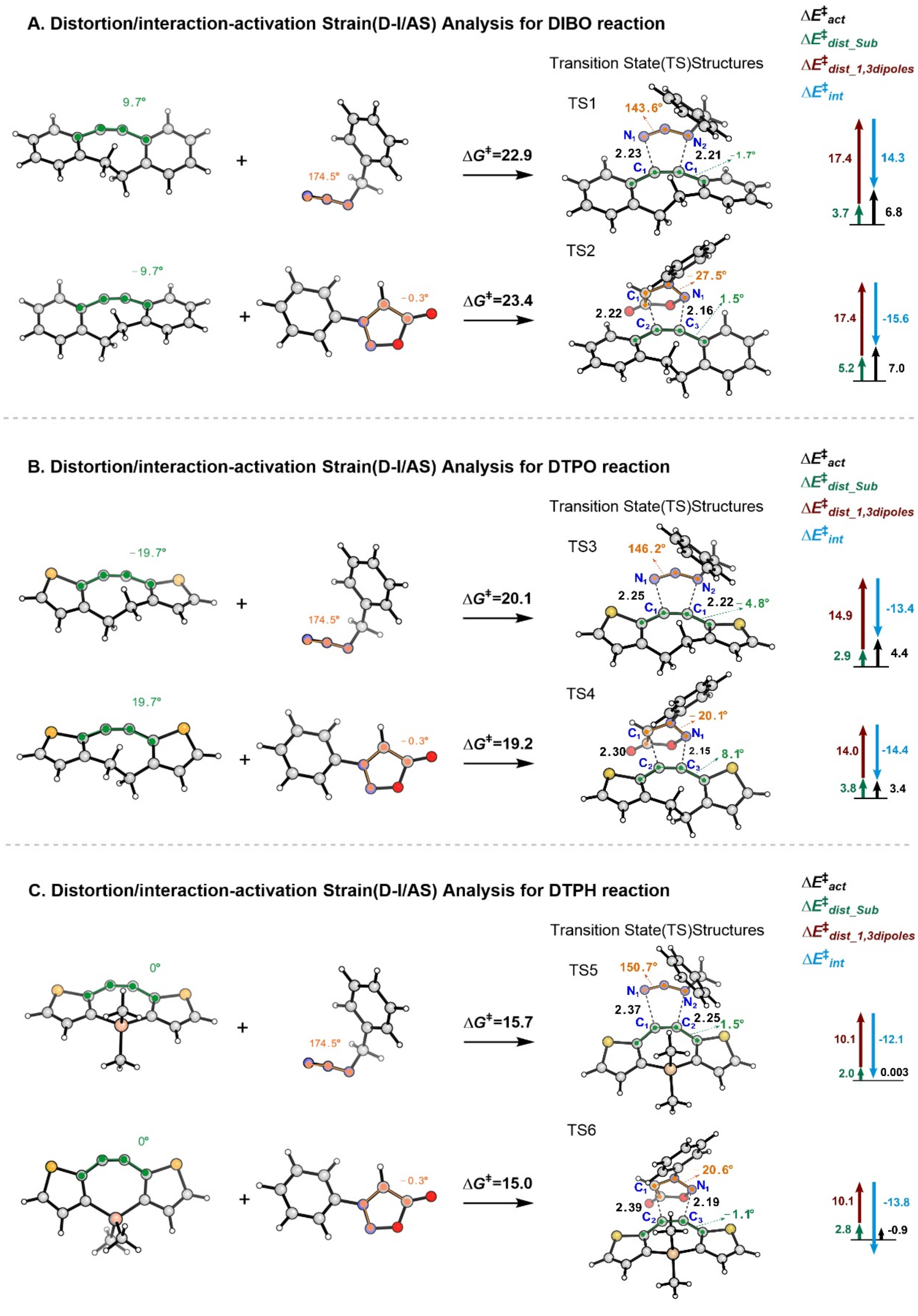

Figure S29. (a) TS Structures and D-I/AS Analysis of DIBO reaction; (b) TS Structures and D-I/AS Analysis of DTPO reaction; (c) TS Structures and D-I/AS Analysis of DTPH reaction; Energies are in kcal/mol

Cartesian Coordinates of Calculated Structure

DIBO

E=-616.800752

H=-616.55948

G=-616.605451

Imag. Freq. -453.68

Cartesian coordinates

C	1.381823	3.118397	0.000000
C	0.224894	5.220003	-0.226199
C	-0.989226	4.571701	0.006549
C	-1.020406	3.195661	0.229013
C	0.166317	2.468057	0.229594
H	0.234275	6.292367	-0.405585
H	-1.965260	2.690987	0.406854
H	0.157793	1.397769	0.412612
C	2.670767	2.499711	0.033656
C	3.886037	2.500294	0.015981
C	5.175282	3.118140	0.049571
C	7.577525	3.194363	-0.179092
C	6.333001	5.219344	0.275492
C	7.546882	4.570462	0.043082
H	8.522204	2.689273	-0.356680
H	6.324036	6.291751	0.454637
H	8.470361	5.142285	0.040089
C	5.128382	4.515858	0.292374
C	3.813433	5.206752	0.599160
H	3.376650	4.740198	1.490564
H	4.029262	6.243689	0.872699
C	1.429248	4.516038	-0.243117
C	2.744328	5.206501	-0.550274
H	3.181046	4.739364	-1.441403
H	2.528679	6.243320	-0.824403
C	6.390520	2.467213	-0.179727
H	6.398638	1.396889	-0.362533
H	-1.912457	5.143925	0.009571

DTPO

E=-1258.340263

H=-1258.166701

G=-1258.212232

Imag. Freq. -400.29

Cartesian coordinates

C	3.417389	3.877936	0.000000
C	4.486305	4.448738	0.136993

C	3.500802	6.543429	-1.575442
H	3.497503	5.699265	-2.274462
H	3.399144	7.447396	-2.182593
C	2.235433	6.424997	-0.641521
H	2.548100	6.606900	0.393100
H	1.539343	7.228213	-0.899243
C	5.336260	5.547186	-0.116920
C	4.843744	6.591259	-0.885610
S	6.911709	5.879920	0.519274
C	5.775829	7.670096	-0.941491
C	6.927017	7.425673	-0.245107
H	5.598009	8.587955	-1.491783
H	7.793872	8.065992	-0.147812
C	2.041811	3.905745	-0.317650
C	1.481032	5.117519	-0.693397
S	0.925913	2.589547	-0.455878
C	0.123901	4.949421	-1.100482
C	-0.308544	3.654840	-1.016942
H	-0.508408	5.766774	-1.430682
H	-1.293033	3.266583	-1.242491

DTPH

E=-1549.073145

H=-1548.878787

G=-1548.931556

Imag. Freq. -308.66

Cartesian coordinates

C	-0.365119	0.611915	0.000000
C	0.722129	1.157626	0.041786
C	1.501457	2.245254	-0.424769
C	0.922468	3.125728	-1.328752
S	3.125174	2.666504	-0.004408
C	1.855774	4.161092	-1.667359
C	3.064534	4.043737	-1.039734
H	1.638215	4.969734	-2.357878
H	3.926920	4.691588	-1.128477
C	-1.671942	0.653018	-0.546225
C	-1.976718	1.671583	-1.439097
S	-3.002473	-0.408078	-0.239386
C	-3.341375	1.554625	-1.865155
H	-3.816845	2.234641	-2.564818
Si	-0.824026	3.044058	-2.091928
C	-0.623440	2.783667	-3.943462
H	-0.131597	1.827611	-4.155564
H	-0.017841	3.584109	-4.385195
H	-1.599236	2.783275	-4.443946
C	-1.664473	4.695704	-1.773929
H	-1.795772	4.872186	-0.700482
H	-1.070129	5.520595	-2.184581
H	-2.652784	4.728015	-2.247836
C	-4.005901	0.496913	-1.309861
H	-5.033268	0.197104	-1.470888

BnN_3

E=-435.10507

H=-434.962977

G=-435.002081

Imag. Freq. nan

Cartesian coordinates

N	1.898310	3.726029	0.000000
N	1.377044	3.155062	0.831795
N	0.866825	2.440098	1.692277
C	-0.137376	3.125238	2.553263
H	-0.494890	2.343902	3.224542
H	0.367810	3.888560	3.155637
C	-1.272219	3.726557	1.760351
C	-1.281819	5.090570	1.457653
C	-2.306660	2.914959	1.281792
C	-2.310038	5.637844	0.689203
H	-0.485171	5.731470	1.828547
C	-3.333201	3.458587	0.514232
H	-2.305965	1.852417	1.513275
C	-3.336298	4.822677	0.216195
H	-2.308360	6.700167	0.462981
H	-4.133357	2.820042	0.151110
H	-4.138327	5.247367	-0.380611

ArSydH

E=-568.294009

H=-568.152384

G=-568.192813

Imag. Freq. nan

Cartesian coordinates

C	3.903495	4.834957	0.000000
C	4.018509	6.088605	-0.591191
C	2.739340	4.078017	-0.076985
C	2.921542	6.598225	-1.280148
H	4.947097	6.644653	-0.522913
C	1.654664	4.599772	-0.776285
H	2.674391	3.110525	0.410144
C	1.744343	5.856401	-1.374559
H	2.992812	7.573288	-1.750776
H	0.737028	4.024902	-0.844558
H	0.894321	6.258380	-1.916803
N	5.028140	4.300125	0.717577
N	5.675203	5.083901	1.528797
O	6.664717	4.311284	2.030635
C	6.612591	3.003007	1.519123
O	7.429465	2.165299	1.862765
C	5.504297	3.046151	0.646377
H	5.094729	2.295124	-0.006859

TS1 (DIBO-BnN_3)

E=-1051.894614

H=-1051.510281

G=-1051.573688

Imag. Freq. -430.94

Cartesian coordinates

C	2.296776	3.798101	0.000000
C	3.649049	3.869945	0.507444
C	4.706048	4.514438	0.631577
C	5.513167	5.683329	0.388953
N	4.230701	1.826148	1.193785
N	5.355740	2.078212	1.295614
C	3.963832	6.086885	-1.567713
H	4.094450	5.059798	-1.930206
H	3.926038	6.726962	-2.454043
N	6.177948	3.020162	1.312678
C	7.404900	2.846669	0.497319
H	7.910518	1.930196	0.816490
H	8.045237	3.689127	0.759931
C	2.610928	6.201665	-0.790770
H	2.818241	6.557301	0.225900
H	1.994811	6.973124	-1.262107
C	6.598966	6.051633	1.190039
H	6.850398	5.442865	2.053477
C	7.353223	7.178756	0.879823
H	8.197476	7.453866	1.505122
C	7.024023	7.946548	-0.236586
H	7.614349	8.822242	-0.490726
C	5.930030	7.593161	-1.026421
H	5.672317	8.199913	-1.891321
C	5.158174	6.469509	-0.731327
C	0.522993	4.863427	-1.251816
H	0.136697	5.725598	-1.790333
C	1.800262	4.931356	-0.692288
C	-0.268182	3.722651	-1.123481
H	-1.260723	3.699475	-1.564336
C	0.216421	2.620107	-0.423185
H	-0.393917	1.728579	-0.311855
C	1.492741	2.658240	0.129857
H	1.877690	1.796984	0.663454
C	7.111889	2.836677	-0.986132
C	6.578508	1.696832	-1.597068
C	7.322101	3.983343	-1.756425
C	6.239905	1.710834	-2.948868
H	6.426004	0.790676	-1.014888
C	6.986247	3.999547	-3.108762
H	7.737910	4.874847	-1.293473
C	6.438858	2.865153	-3.706683
H	5.823659	0.820081	-3.410629
H	7.148128	4.900977	-3.693139
H	6.172790	2.878073	-4.759801

TS2 (DIBO-ArSydH)

E=-1185.083571

H=-1184.699312

G=-1184.763977

Imag. Freq. -453.68

Cartesian coordinates

C	2.821116	7.954401	0.036930
C	4.059349	8.205385	-0.673180
C	5.130440	8.857473	-0.750765
C	6.035523	9.856913	-0.229022
C	4.563699	9.912739	1.838446
H	4.671574	8.832956	1.996637
H	4.581343	10.372589	2.830692
C	3.180544	10.191364	1.164900
H	3.340789	10.689892	0.201280
H	2.609576	10.888567	1.784661
C	7.146259	10.321832	-0.946742
H	7.359349	9.897533	-1.920528
C	7.962587	11.319989	-0.424372
H	8.818499	11.669398	-0.994325
C	7.675760	11.868949	0.823476
H	8.307501	12.649649	1.237385
C	6.573524	11.411501	1.544313
H	6.353090	11.840246	2.518912
C	5.744947	10.406496	1.044242
C	1.184630	8.716043	1.646477
H	0.833360	9.471886	2.344845
C	2.371172	8.940463	0.946986
C	0.447363	7.546754	1.467198
H	-0.474158	7.396316	2.022087
C	0.898339	6.573926	0.576987
H	0.336432	5.655556	0.435173
C	2.075722	6.780009	-0.134383
H	2.429307	6.010331	-0.813577
C	4.265621	9.412427	-3.762028
C	5.118751	10.115671	-4.609580
C	3.010046	9.905462	-3.415139
C	4.699907	11.340580	-5.119153
H	6.089608	9.704688	-4.865663
C	2.598502	11.125813	-3.945047
H	2.368854	9.360037	-2.730513
C	3.440578	11.844615	-4.791918
H	5.356155	11.897234	-5.780540
H	1.621404	11.518498	-3.682269
H	3.116302	12.798169	-5.196942
N	4.716753	8.173681	-3.221188
N	5.922359	8.184589	-2.639875
O	6.112303	6.868508	-2.240577
C	4.899131	6.225996	-2.178496
O	4.789503	5.082850	-1.790779
C	3.955942	7.216832	-2.654135
H	2.943681	7.018764	-2.973331

TS3 (DTPO-BnN$_3$)

E=-1693.438252

H=-1693.12183

G=-1693.185328

Imag. Freq. -403.84

Cartesian coordinates

C	1.597334	4.363035	0.000000
C	2.633614	5.028610	0.175633
N	2.186351	2.301174	0.684379
N	3.297204	2.589968	0.802536
C	1.717791	6.868920	-1.914814
H	1.886156	5.924756	-2.446202
H	1.671643	7.652387	-2.676758
N	4.129020	3.520404	0.837347
C	5.327761	3.352368	-0.027735
H	5.854091	2.444170	0.281034
H	5.965661	4.204863	0.206407
C	0.331606	6.799654	-1.176091
H	0.463036	7.176854	-0.155669
H	-0.365011	7.483926	-1.669325
C	4.971606	3.322965	-1.496142
C	4.484636	2.150894	-2.083834
C	5.067795	4.481390	-2.272120
C	4.079565	2.142884	-3.417603
H	4.420568	1.236800	-1.497626
C	4.665049	4.475962	-3.605775
H	5.450399	5.397231	-1.828948
C	4.164081	3.307802	-4.179800
H	3.700561	1.226725	-3.861300
H	4.740188	5.385348	-4.195238
H	3.846401	3.303482	-5.218552
C	3.319539	6.249402	-0.052228
C	2.896379	7.140953	-1.019730
S	4.697831	6.841544	0.818961
C	3.712954	8.311469	-1.045167
C	4.720831	8.287077	-0.122433
H	3.556560	9.135231	-1.733867
H	5.475375	9.040590	0.061061
C	0.274078	4.338742	-0.511811
C	-0.314074	5.439111	-1.111703
S	-0.751624	2.937579	-0.585958
C	-1.603463	5.122245	-1.635285
C	-1.970922	3.823149	-1.419855
H	-2.235424	5.843133	-2.143553
H	-2.897675	3.339394	-1.699531

TS4 (DTPO-ArSydH)

E=-1826.628827

H=-1826.312655

G=-1826.377452

Imag. Freq. -400.29

Cartesian coordinates

C	0.306056	3.421478	0.000000
C	1.366891	4.086036	-0.071222
C	0.971065	5.012938	2.724628
H	1.097239	3.936947	2.888847
H	1.093953	5.492536	3.699874
C	-0.489819	5.274859	2.208875
H	-0.442371	5.961545	1.356016
H	-1.053319	5.790954	2.991413
C	0.556529	4.632660	-3.125373
C	1.411107	5.355440	-3.954196
C	-0.726853	5.080824	-2.823256
C	0.963172	6.557417	-4.492927
H	2.403579	4.977090	-4.174876
C	-1.165589	6.278654	-3.381435
H	-1.367726	4.518816	-2.151796
C	-0.323566	7.017512	-4.211219
H	1.618556	7.130110	-5.141200
H	-2.164086	6.638321	-3.154712
H	-0.669833	7.953262	-4.638868
N	1.032468	3.414948	-2.555681
N	2.216517	3.463597	-1.940095
O	2.437999	2.156403	-1.534770
C	1.248172	1.462046	-1.529236
O	1.170251	0.316338	-1.144935
C	0.292848	2.407085	-2.065357
H	-0.696198	2.174857	-2.430410
C	-0.883864	3.224135	0.755160
C	-1.258943	4.046197	1.803856
S	-2.008930	1.919161	0.528659
C	-2.461632	3.590810	2.422788
C	-2.978237	2.466001	1.843261
H	-2.927840	4.094297	3.263294
H	-3.877997	1.929223	2.113588
C	2.247455	5.020039	0.528117
C	2.064344	5.501098	1.812769
S	3.598673	5.774096	-0.262032
C	3.030159	6.498580	2.138844
C	3.916481	6.742193	1.126117
H	3.066518	7.004921	3.097752
H	4.749192	7.433434	1.124964

TS5 (DTPH-BnN$_3$)

E=-1984.178227

H=-1983.841001

G=-1983.911619

Imag. Freq. -298.97

Cartesian coordinates

C	2.078996	3.283506	0.759197
C	1.102177	2.523023	0.843599
N	2.135823	0.395311	0.658248
N	3.147669	0.912640	0.495166
N	3.860236	1.930420	0.516406
C	4.755832	2.147716	-0.648196
H	5.459884	1.311935	-0.704492
H	5.323592	3.044141	-0.397327
C	3.999693	2.325777	-1.943125
C	3.570041	1.211265	-2.670164
C	3.690254	3.605162	-2.412912
C	2.833320	1.373135	-3.842491
H	3.816891	0.210329	-2.322877
C	2.961019	3.769213	-3.588272
H	4.017912	4.478302	-1.853883
C	2.527239	2.653323	-4.303186
H	2.504256	0.499989	-4.398446
H	2.725186	4.768710	-3.941764
H	1.954808	2.780853	-5.217473
C	-0.311932	2.584564	0.969414
C	-1.018275	3.748084	0.693668
S	-1.333172	1.308673	1.553081
C	-2.410188	3.571523	0.984699
C	-2.722417	2.324042	1.450074
H	-3.159267	4.344670	0.847710
H	-3.695045	1.944570	1.735823
C	2.446701	4.654752	0.753345
C	1.503991	5.635267	0.479733
S	3.991800	5.312221	1.184828
C	2.081574	6.939021	0.629503
H	1.537243	7.863127	0.464461
Si	-0.250613	5.268638	-0.134417
C	-1.335908	6.765098	0.195102
H	-1.401288	6.988993	1.265787
H	-2.351746	6.596518	-0.180959
H	-0.940560	7.651147	-0.315209
C	-0.153525	4.907870	-1.976315
H	0.464934	4.021794	-2.160899
H	-1.152290	4.720378	-2.388951
H	0.293300	5.748696	-2.520475
C	3.398501	6.920971	0.994437
H	4.055770	7.763920	1.164234

TS6 (DTPH-ArSydH)

E=-2117.368798

H=-2117.031776

G=-2117.103516

Imag. Freq. -322.07

Cartesian coordinates

C	3.195993	4.455168	-2.157717
C	3.981643	5.395731	-2.818209
C	1.898901	4.740542	-1.739930
C	3.447787	6.656637	-3.064869
H	4.986471	5.139618	-3.136614
C	1.375008	6.002866	-2.005025
H	1.314683	4.002845	-1.199734
C	2.146873	6.959963	-2.661998
H	4.047648	7.399824	-3.580070
H	0.366056	6.239768	-1.683145
H	1.734382	7.944188	-2.860526
N	3.758172	3.173595	-1.875969
N	4.959064	3.156465	-1.300057
O	5.252589	1.814054	-1.173088
C	4.106502	1.058362	-1.319878
O	4.106935	-0.145697	-1.186128
C	3.096372	2.030627	-1.659891
H	2.108849	1.835618	-2.047742
C	4.098065	3.337361	0.709441
C	3.095237	2.595509	0.663011
C	2.029378	2.174460	1.507445
C	1.699746	2.855524	2.671493
S	1.061854	0.753389	1.271262
C	0.647168	2.177094	3.368363
C	0.208835	1.044618	2.740201
H	0.221910	2.523324	4.304753
H	-0.570520	0.364746	3.059312
C	4.812510	4.222248	1.556010
C	4.243395	4.722754	2.718941
S	6.461540	4.716778	1.348175
C	5.197285	5.509455	3.442415
H	4.981517	6.006820	4.382362
Si	2.407990	4.545478	3.146289
C	1.484177	5.842143	2.145094
H	1.649317	5.682565	1.072680
H	0.405233	5.792176	2.334855
H	1.832668	6.851494	2.395136
C	2.169218	4.821610	4.987497
H	2.701303	4.069053	5.580060
H	1.106488	4.775606	5.252317
H	2.537986	5.811047	5.281819
C	6.417977	5.593450	2.831610
H	7.295251	6.130148	3.169210

Rea1 (DIBO-BnN$_3$)

E=-1051.919101

H=-1051.533681

G=-1051.600361

Imag. Freq. 0

Cartesian coordinates

C	1.733011	7.714422	0.202009
C	3.000380	7.940505	0.823178
C	4.041425	8.545414	0.985985
C	5.043793	9.504410	0.649333
N	4.163909	5.078159	2.041051
N	5.135086	5.657452	1.931323
C	3.616157	9.795158	-1.445510
H	3.734824	8.740788	-1.724145
H	3.683729	10.363586	-2.378110
N	6.137900	6.364728	1.905895
C	7.097019	6.075836	0.804702
H	7.495538	5.063723	0.941244
H	7.910027	6.784865	0.962407
C	2.185321	10.000972	-0.836329
H	2.280542	10.475160	0.148018
H	1.637228	10.709102	-1.464883
C	6.178227	9.787630	1.412856
H	6.347917	9.241375	2.335444
C	7.087907	10.744604	0.972969
H	7.974888	10.960707	1.561111
C	6.856644	11.420869	-0.224555
H	7.564842	12.167150	-0.573173
C	5.720173	11.138398	-0.984410
H	5.558459	11.664403	-1.922363
C	4.796298	10.177303	-0.574201
C	0.150949	8.583016	-1.394619
H	-0.168851	9.358700	-2.086383
C	1.342453	8.748561	-0.688314
C	-0.639756	7.445151	-1.222948
H	-1.565136	7.343903	-1.782728
C	-0.248170	6.443772	-0.335213
H	-0.864331	5.559698	-0.200906
C	0.941478	6.576144	0.375753
H	1.267711	5.799588	1.061177
C	6.502707	6.239085	-0.573919
C	5.736765	5.213459	-1.139383
C	6.692090	7.420241	-1.295830
C	5.152860	5.374531	-2.394755
H	5.602033	4.277657	-0.601449
C	6.119411	7.578840	-2.555569
H	7.280010	8.227263	-0.865406
C	5.343370	6.559332	-3.105346
H	4.556902	4.572338	-2.820443
H	6.268310	8.506789	-3.100538
H	4.890599	6.686582	-4.084566

Rea2 (DIBO-ArSydH)

E=-1185.110766

H=-1184.725663

G=-1184.793684

Imag. Freq. 0

Cartesian coordinates

C	2.745496	8.702496	0.000000
C	4.049577	8.989009	-0.509447
C	4.995336	9.711686	-0.752259
C	5.718815	10.942863	-0.717054
C	4.000264	11.527769	1.075057
H	4.278397	10.653437	1.675276
H	3.842211	12.347160	1.782281
C	2.643600	11.236566	0.341235
H	2.777289	11.442069	-0.727012
H	1.897619	11.954836	0.693765
C	6.812550	11.259729	-1.525738
H	7.207529	10.511449	-2.206238
C	7.368865	12.534032	-1.467621
H	8.215100	12.784778	-2.100251
C	6.829653	13.486972	-0.604395
H	7.258057	14.484179	-0.560108
C	5.740949	13.166070	0.208645
H	5.333690	13.917241	0.881158
C	5.166574	11.895406	0.177598
C	0.803481	9.646013	1.066536
H	0.257111	10.501938	1.455395
C	2.059335	9.843522	0.491571
C	0.231351	8.374748	1.138810
H	-0.750303	8.251935	1.587258
C	0.909637	7.267843	0.630322
H	0.460780	6.280317	0.679300
C	2.170067	7.430574	0.062685
H	2.713742	6.578241	-0.334297
C	4.560491	10.284956	-4.102519
C	5.561103	10.827205	-4.900976
C	3.549867	11.061637	-3.547531
C	5.549420	12.198528	-5.136957
H	6.329453	10.191101	-5.326480
C	3.549808	12.430280	-3.796251
H	2.793381	10.611118	-2.913861
C	4.550403	12.999028	-4.583740
H	6.323233	12.640080	-5.756333
H	2.773617	13.052054	-3.362039
H	4.550200	14.068630	-4.768586
N	4.596819	8.884464	-3.791854
N	5.754316	8.345687	-3.542554
O	5.473651	7.062830	-3.226735
C	4.091213	6.815707	-3.248322
O	3.649340	5.712271	-2.972987
C	3.550359	8.058674	-3.639564
H	2.531381	8.349379	-3.827447

Rea3 (DTPO-BnN$_3$)

E=-1693.458453

H=-1693.14082

G=-1693.207387

Imag. Freq. 0

Cartesian coordinates

C	1.925599	4.812408	0.000000
C	2.859777	5.554705	0.246539
N	2.881393	2.160017	1.525598
N	3.893378	2.638496	1.333318
C	2.213186	7.038667	-2.150287
H	2.477486	6.078231	-2.606208
H	2.199556	7.772470	-2.961646
N	4.960461	3.234057	1.211283
C	5.770094	2.819795	0.030880
H	5.991585	1.749165	0.109250
H	6.705367	3.369835	0.135526
C	0.756442	6.919758	-1.560147
H	0.747123	7.358525	-0.555824
H	0.085700	7.530563	-2.171213
C	5.099388	3.138885	-1.283155
C	4.185768	2.242879	-1.847091
C	5.355347	4.348112	-1.934392
C	3.527500	2.556680	-3.035192
H	3.990639	1.290119	-1.359834
C	4.708780	4.658621	-3.127686
H	6.058615	5.055137	-1.500925
C	3.789013	3.765901	-3.678081
H	2.815933	1.854809	-3.460638
H	4.914120	5.604132	-3.621797
H	3.278449	4.012085	-4.604883
C	3.657827	6.669950	-0.081413
C	3.319033	7.421621	-1.195493
S	5.016828	7.319216	0.769363
C	4.190462	8.543011	-1.338634
C	5.150421	8.610395	-0.367065
H	4.110867	9.264162	-2.145423
H	5.935829	9.346532	-0.257328
C	0.717659	4.536182	-0.672614
C	0.182052	5.524533	-1.484054
S	-0.150209	3.043094	-0.785187
C	-0.951383	5.038955	-2.202063
C	-1.248949	3.733567	-1.922076
H	-1.528894	5.644839	-2.892318
H	-2.064989	3.139209	-2.311606

Rea4 (DTPO-ArSydH)

E=-1826.649525

H=-1826.332069

G=-1826.399636

Imag. Freq. 0

Cartesian coordinates

C	0.075017	3.705117	0.000000
C	1.067338	4.365253	-0.255980
C	0.091060	6.411761	1.499647
H	0.255668	5.578134	2.191685
H	-0.011882	7.309868	2.115078
C	-1.267901	6.176401	0.737012
H	-1.105977	6.404869	-0.321590
H	-1.998100	6.906186	1.098352
C	0.125750	4.989022	-3.511247
C	0.952989	5.750301	-4.329831
C	-1.018107	5.512433	-2.918520
C	0.619757	7.082755	-4.553598
H	1.833128	5.308843	-4.783946
C	-1.342767	6.843583	-3.161582
H	-1.630224	4.904098	-2.259819
C	-0.524694	7.628754	-3.972879
H	1.253981	7.691733	-5.189390
H	-2.229700	7.268535	-2.702742
H	-0.779913	8.668170	-4.153570
N	0.474701	3.623646	-3.242167
N	1.732018	3.329340	-3.085249
O	1.741206	2.008389	-2.799917
C	0.440620	1.482247	-2.745671
O	0.252458	0.304838	-2.488898
C	-0.368266	2.597139	-3.053644
H	-1.435114	2.673681	-3.172452
C	-1.267626	3.637797	0.432089
C	-1.894318	4.804769	0.844579
S	-2.268277	2.240509	0.634512
C	-3.206951	4.532358	1.334441
C	-3.543772	3.208521	1.274170
H	-3.878480	5.299604	1.705127
H	-4.478695	2.746221	1.562321
C	1.819286	5.553927	-0.159792
C	1.317595	6.574999	0.633515
S	3.231774	6.028567	-1.038200
C	2.109023	7.754975	0.508887
C	3.168727	7.605200	-0.343272
H	1.900935	8.676194	1.042721
H	3.927702	8.334555	-0.594179

Rea5 (DTPH-BnN$_3$)

E=-1984.195536

H=-1983.857035

G=-1983.929651

Imag. Freq. 0

Cartesian coordinates

C	0.627288	3.285039	0.000000
C	1.560644	4.042191	0.203532
N	1.758962	0.608412	1.345772
N	2.734833	1.095287	1.027208
N	3.771551	1.702776	0.776282
C	4.454271	1.288668	-0.480759
H	4.732174	0.231302	-0.399321
H	5.370009	1.879886	-0.498830
C	3.632149	1.534684	-1.723216
C	2.661346	0.610612	-2.126117
C	3.820012	2.692487	-2.480820
C	1.882458	0.849185	-3.255990
H	2.515606	-0.305290	-1.557525
C	3.054343	2.924340	-3.620971
H	4.564385	3.423712	-2.174281
C	2.079988	2.006675	-4.007420
H	1.121848	0.131848	-3.549988
H	3.214296	3.831220	-4.197292
H	1.469524	2.195111	-4.886041
C	2.407488	5.014054	-0.381991
C	2.026538	5.550791	-1.604934
S	3.991522	5.497799	0.111383
C	3.077914	6.367449	-2.131417
C	4.180406	6.435015	-1.324534
H	3.018885	6.893599	-3.078543
H	5.092696	6.993751	-1.489015
C	-0.295594	2.821284	-0.969228
C	-0.436204	3.565657	-2.132964
S	-1.179522	1.336349	-1.000033
C	-1.289049	2.879309	-3.055641
H	-1.547221	3.259992	-4.038423
Si	0.308409	5.297672	-2.383170
C	0.404092	5.650647	-4.224803
H	1.043921	4.932017	-4.747745
H	0.798620	6.656719	-4.408818
H	-0.595094	5.603651	-4.673705
C	-0.851862	6.518815	-1.545891
H	-0.913469	6.316729	-0.469957
H	-0.497181	7.548027	-1.678807
H	-1.862909	6.445476	-1.964331
C	-1.761713	1.685650	-2.585391
H	-2.425603	0.989871	-3.081976

Rea6 (DTPH-ArSydH)

E=-1826.649525

H=-1826.332069

G=-1826.399636

Imag. Freq. 0

Cartesian coordinates

C	0.075017	3.705117	0.000000
C	1.067338	4.365253	-0.255980
C	0.091060	6.411761	1.499647
H	0.255668	5.578134	2.191685
H	-0.011882	7.309868	2.115078
C	-1.267901	6.176401	0.737012
H	-1.105977	6.404869	-0.321590
H	-1.998100	6.906186	1.098352
C	0.125750	4.989022	-3.511247
C	0.952989	5.750301	-4.329831
C	-1.018107	5.512433	-2.918520
C	0.619757	7.082755	-4.553598
H	1.833128	5.308843	-4.783946
C	-1.342767	6.843583	-3.161582
H	-1.630224	4.904098	-2.259819
C	-0.524694	7.628754	-3.972879
H	1.253981	7.691733	-5.189390
H	-2.229700	7.268535	-2.702742
H	-0.779913	8.668170	-4.153570
N	0.474701	3.623646	-3.242167
N	1.732018	3.329340	-3.085249
O	1.741206	2.008389	-2.799917
C	0.440620	1.482247	-2.745671
O	0.252458	0.304838	-2.488898
C	-0.368266	2.597139	-3.053644
H	-1.435114	2.673681	-3.172452
C	-1.267626	3.637797	0.432089
C	-1.894318	4.804769	0.844579
S	-2.268277	2.240509	0.634512
C	-3.206951	4.532358	1.334441
C	-3.543772	3.208521	1.274170
H	-3.878480	5.299604	1.705127
H	-4.478695	2.746221	1.562321
C	1.819286	5.553927	-0.159792
C	1.317595	6.574999	0.633515
S	3.231774	6.028567	-1.038200
C	2.109023	7.754975	0.508887
C	3.168727	7.605200	-0.343272
H	1.900935	8.676194	1.042721
H	3.927702	8.334555	-0.594179

Protein Modification

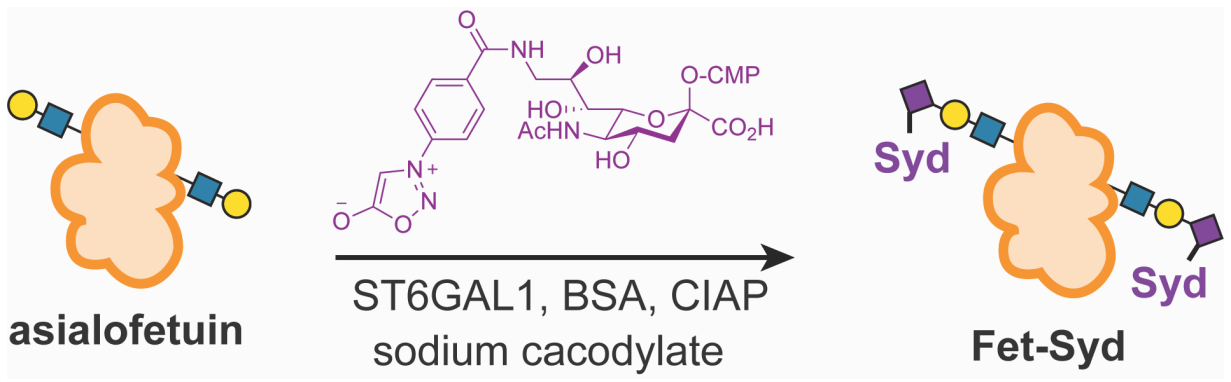

Scheme S2. Formation of fetuin having sialic acids displaying a sydnone reporter

rh-ST6GAL1 (10 µL, 10 µg) was added to a solution of asialofetuin (4.1 mg) and CMP-Neu9SydH (2.8 mg) in sodium cacodylate buffer (1.04 mL, 50 mM, pH 7.5) containing BSA (0.4%) and CIAP (19.4 µL, 1.9 U). The reaction was incubated at 37 °C for 18 h, spin-filtered (MWCO = 10 kDa) and washed with PBS buffer (pH 7.4). The modified fetuin was stored in PBS buffer (pH 7.4) at –20 °C.

Protein labeling.

Glycoprotein fetuin bearing sydnone-modified sialic acids (Fet-Syd, Scheme S2) and native fetuin (0.1 mg/mL final concentration) were incubated with the probes DTPO, photo-DTPO, or photo-DTPH (0-25 µM) in PBS (pH 7.4, containing 20% of DMF) or U2OS cell lysate (containing 20% of DMF) for 0-15 min at room temperature. To activate photo-DTPO or photo-DTPH in situ, the protein suspension was subjected to UV light (350 nm) for 2 min immediately after adding the compounds to the protein samples. Labeled proteins were then analyzed for fluorescence intensity (295 ex/ 385 em) using a microplate reader (SpectraMax M2E Reader from molecular devices) or via SDS-PAGE (10% gradient) and visualized by in-gel fluorescence scanning (BioRad, ChemiDoc MP imager, trans-UV excitation (302 nm)/standard emission filter). The gels were then stained with Coomassie Blue to reveal total protein content. Data points were collected in triplicate and are representative of three separate experiments.

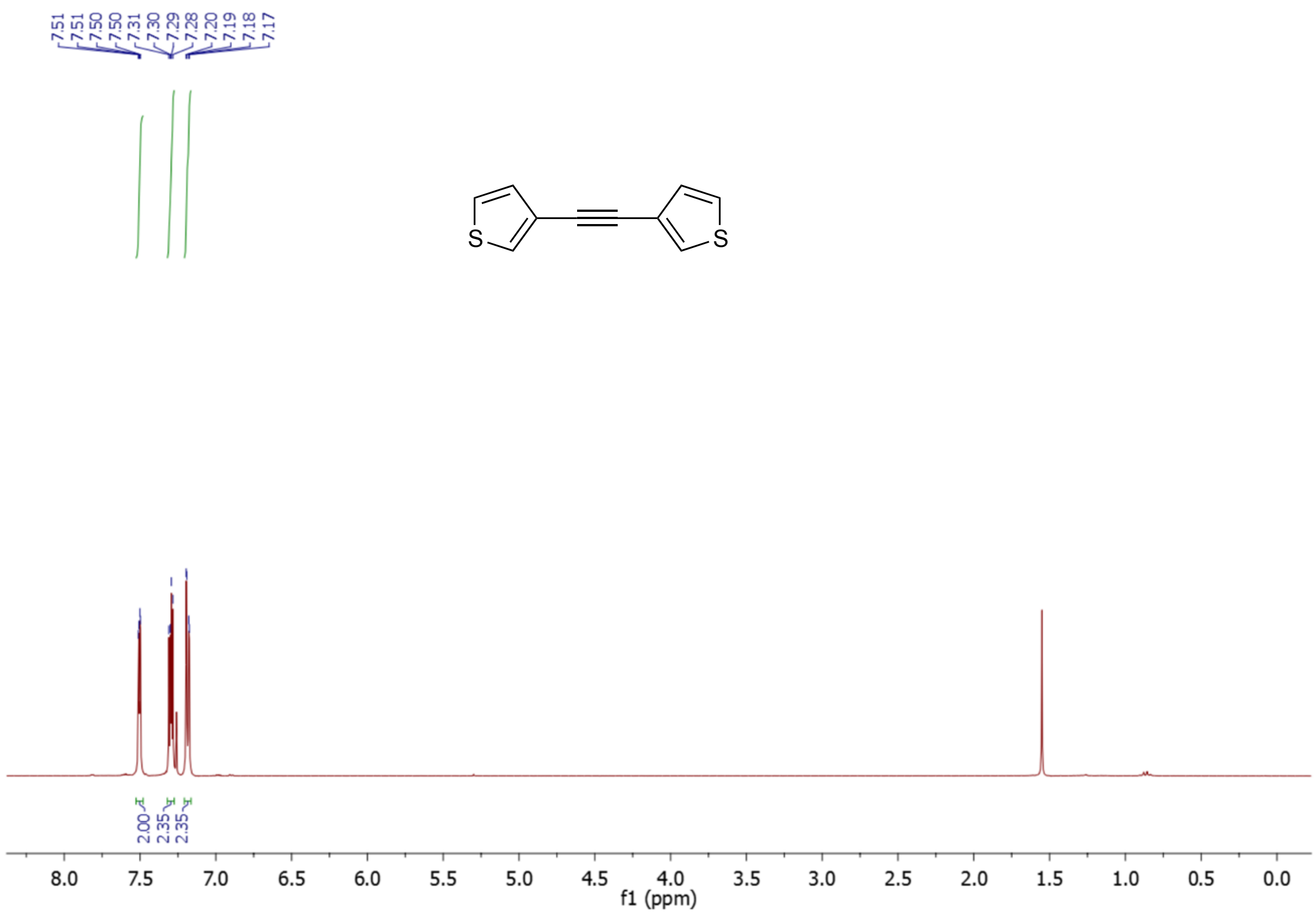
7.51
7.51
7.50
7.50
7.31
7.30
7.29
7.28
7.20
7.19
7.18
7.17
S
S
2.00
2.35
2.35
8.0 7.5 7.0 6.5 6.0 5.5 5.0 4.5 4.0 3.5 3.0 2.5 2.0 1.5 1.0 0.5 0.0
f1 (ppm)

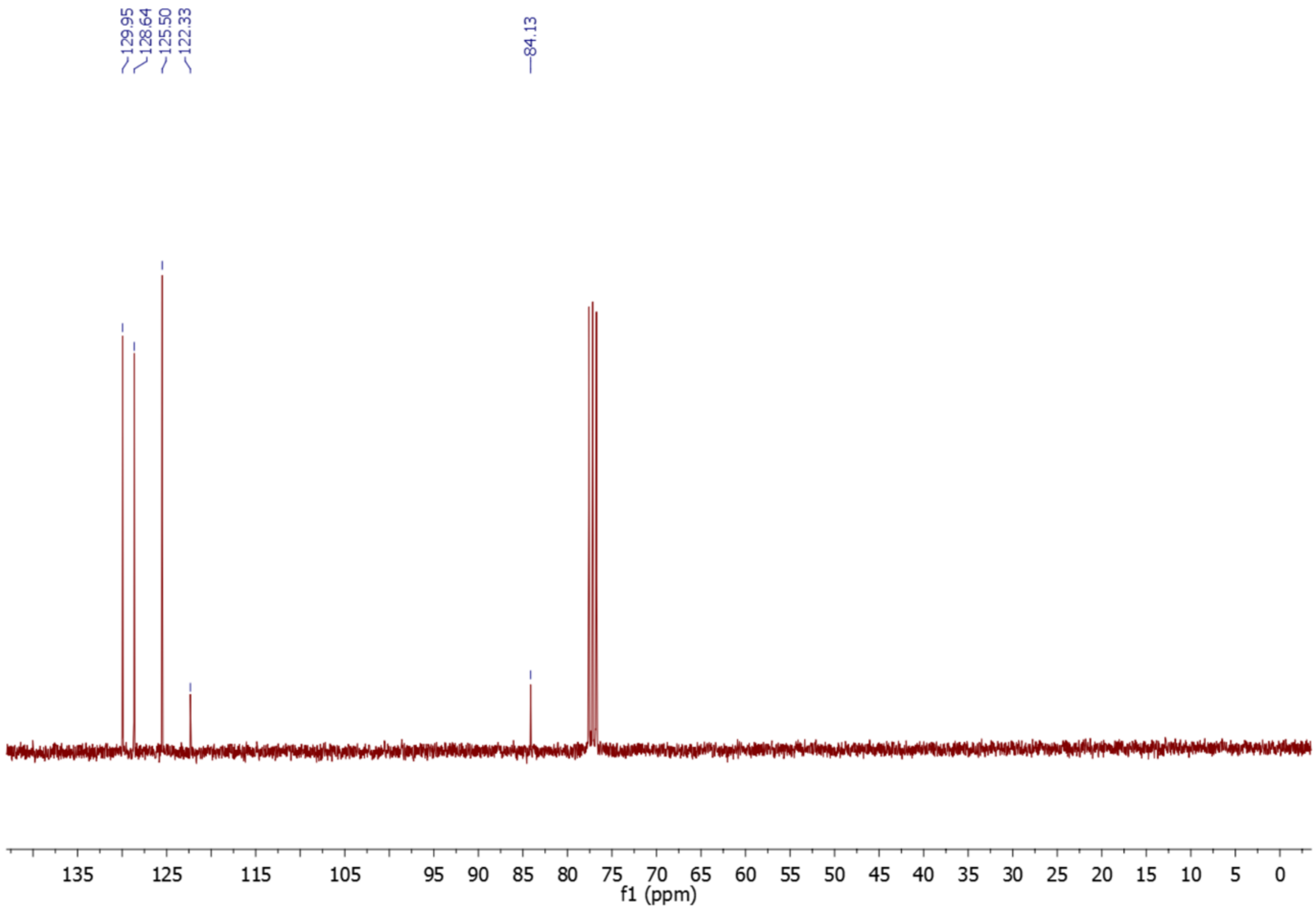
129.95
128.64
125.50
122.33
84.13
135 125 115 105 95 90 85 80 75 70 65 60 55 50 45 40 35 30 25 20 15 10 5 0
f1 (ppm)

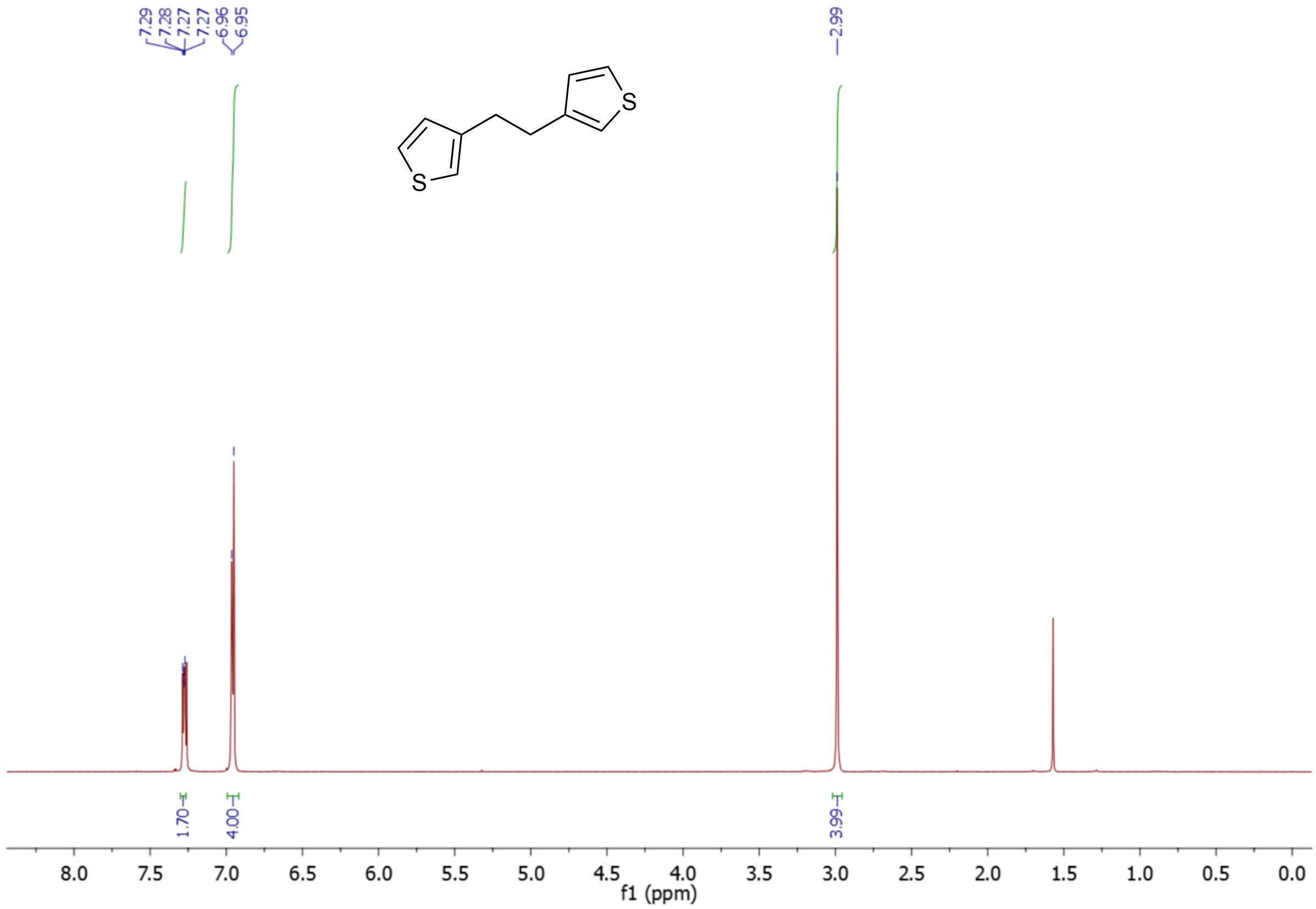

7.29
7.28
7.27
7.27
6.96
6.95
2.99
S
S
1.70
4.00
3.99
8.0 7.5 7.0 6.5 6.0 5.5 5.0 4.5 4.0 3.5 3.0 2.5 2.0 1.5 1.0 0.5 0.0
f1 (ppm)

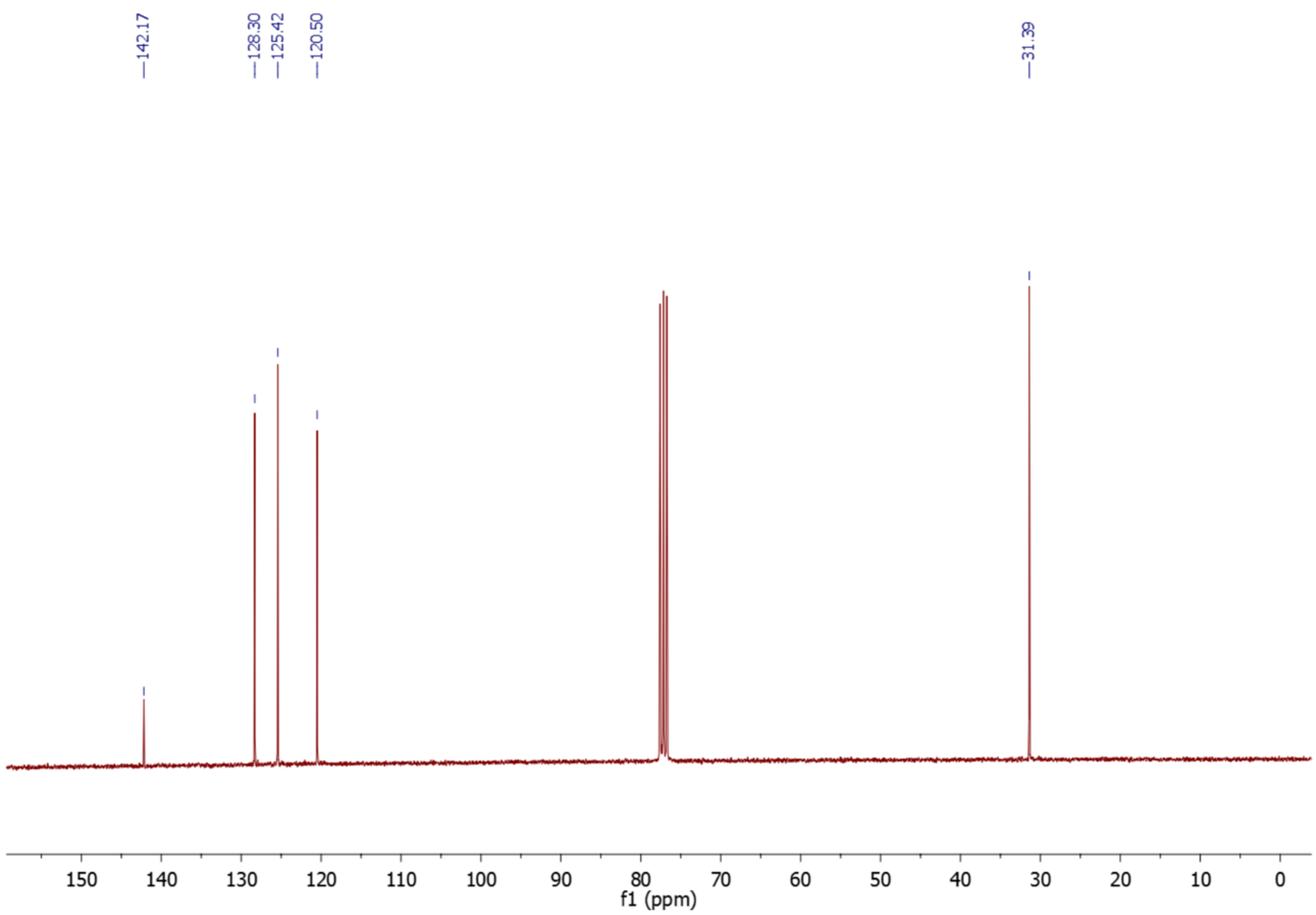

142.17
128.30
125.42
120.50
31.39
150 140 130 120 110 100 90 80 70 60 50 40 30 20 10 0
f1 (ppm)

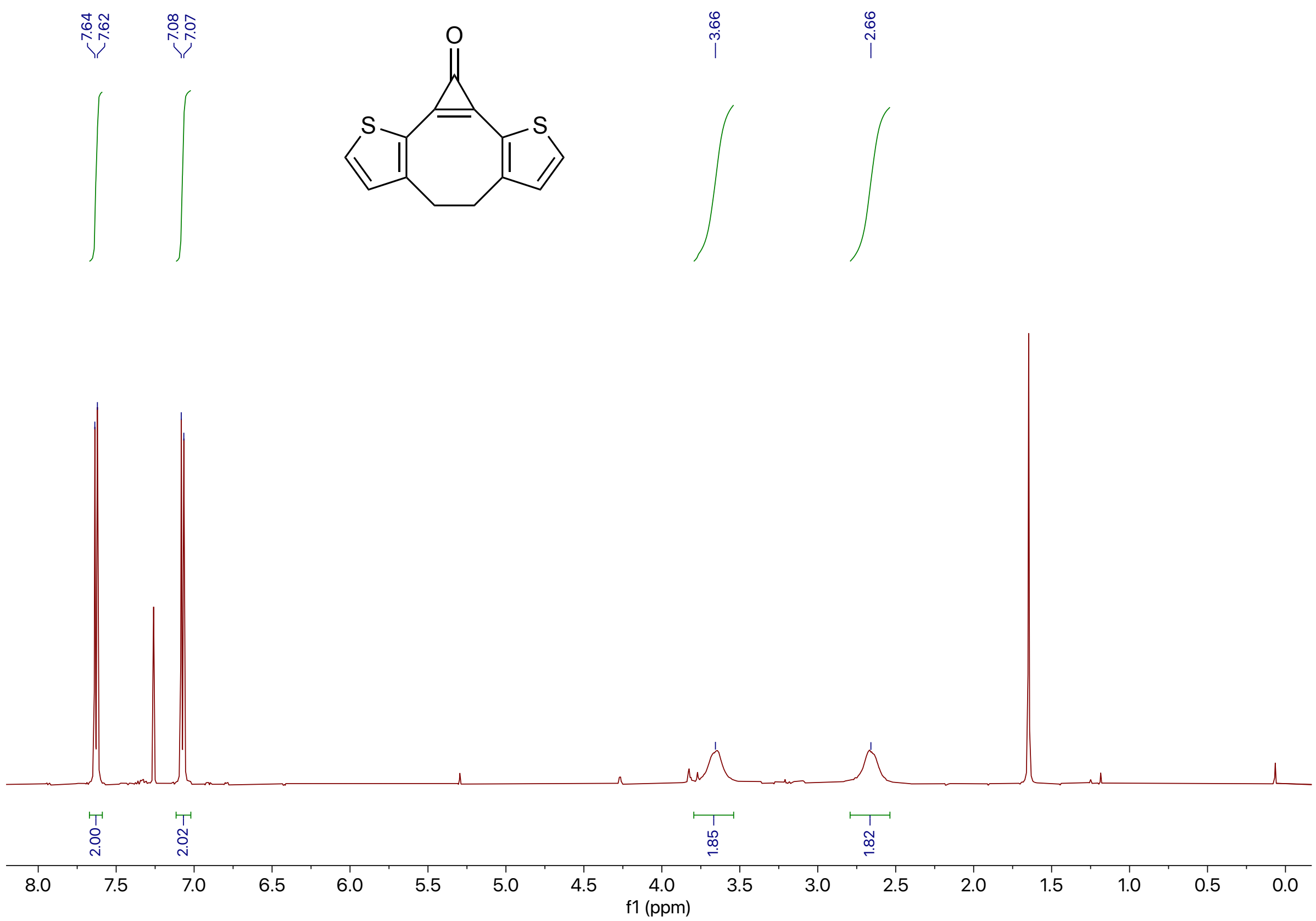

7.64
7.62
7.08
7.07
O
S
S
3.66
2.66
2.00
2.02
1.85
1.82
8.0 7.5 7.0 6.5 6.0 5.5 5.0 4.5 4.0 3.5 3.0 2.5 2.0 1.5 1.0 0.5 0.0
f1 (ppm)

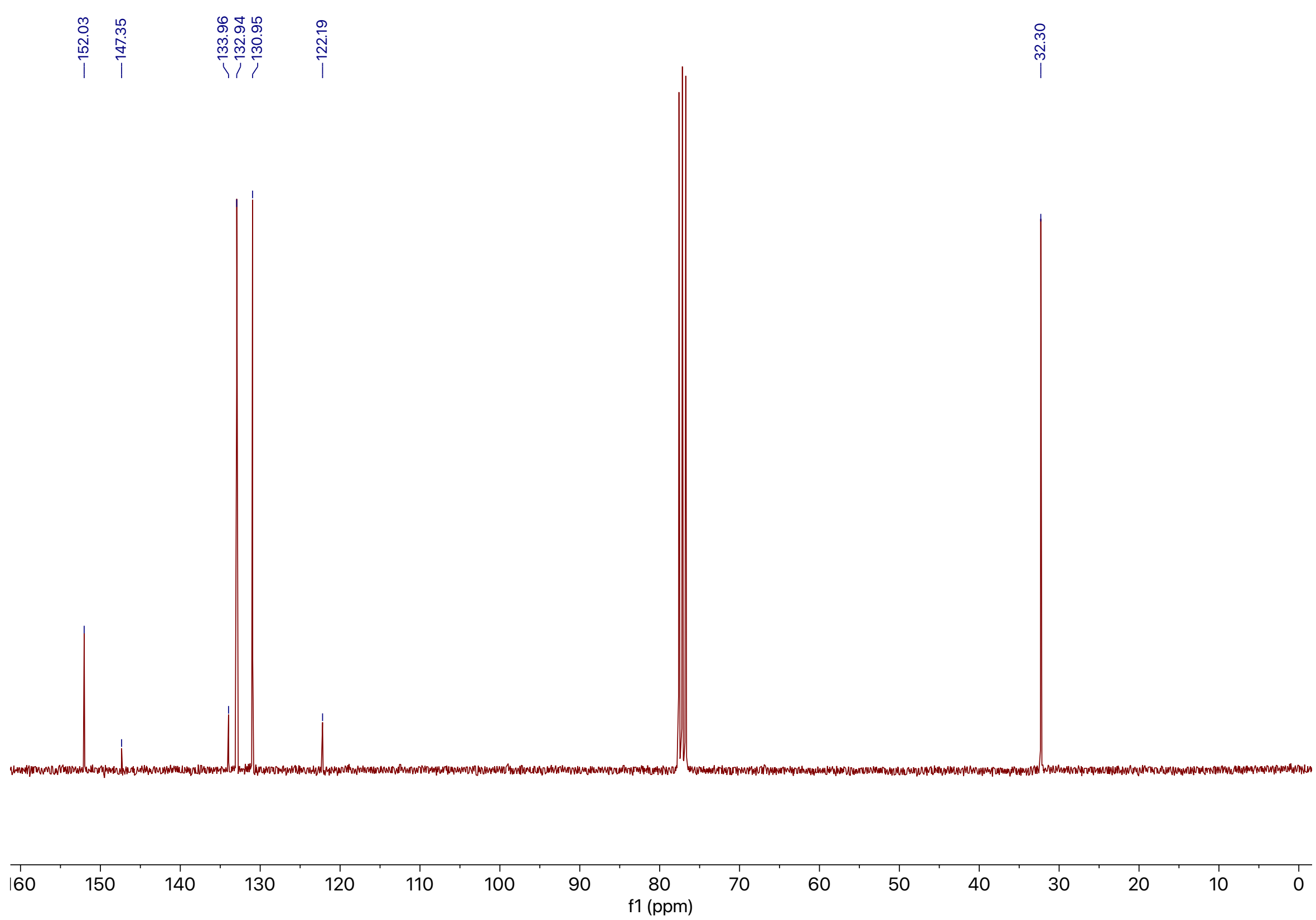

152.03
147.35
133.96
132.94
130.95
122.19
32.30
160 150 140 130 120 110 100 90 80 70 60 50 40 30 20 10 0
f1 (ppm)

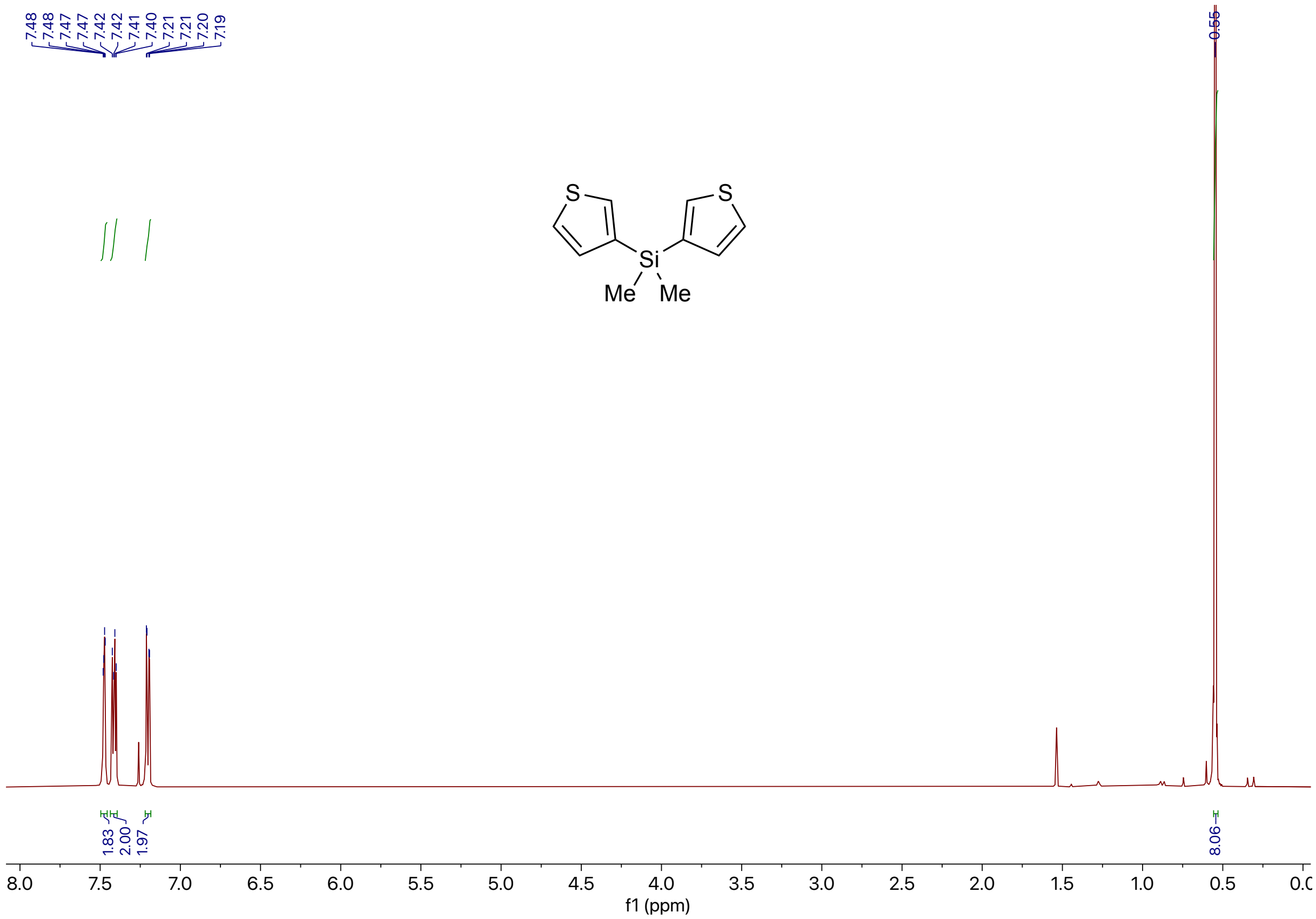
7.48
7.48
7.47
7.47
7.42
7.42
7.41
7.40
7.21
7.21
7.20
7.19
0.55
S
S
Si
Me
Me
1.83
2.00
1.97
8.06
8.0 7.5 7.0 6.5 6.0 5.5 5.0 4.5 4.0 3.5 3.0 2.5 2.0 1.5 1.0 0.5 0.0
f1 (ppm)

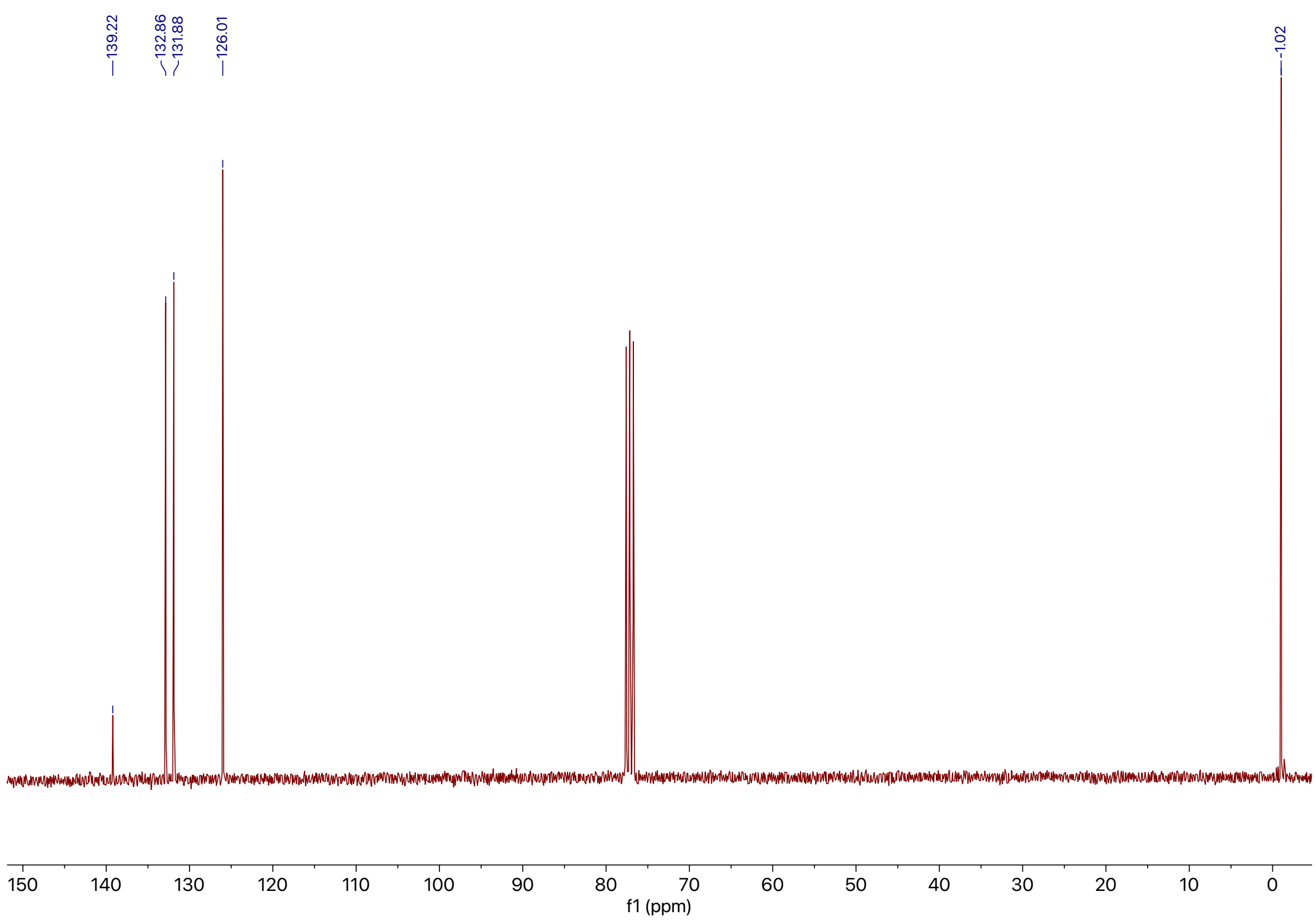
139.22
132.86
131.88
126.01
-1.02
150 140 130 120 110 100 90 80 70 60 50 40 30 20 10 0
f1 (ppm)

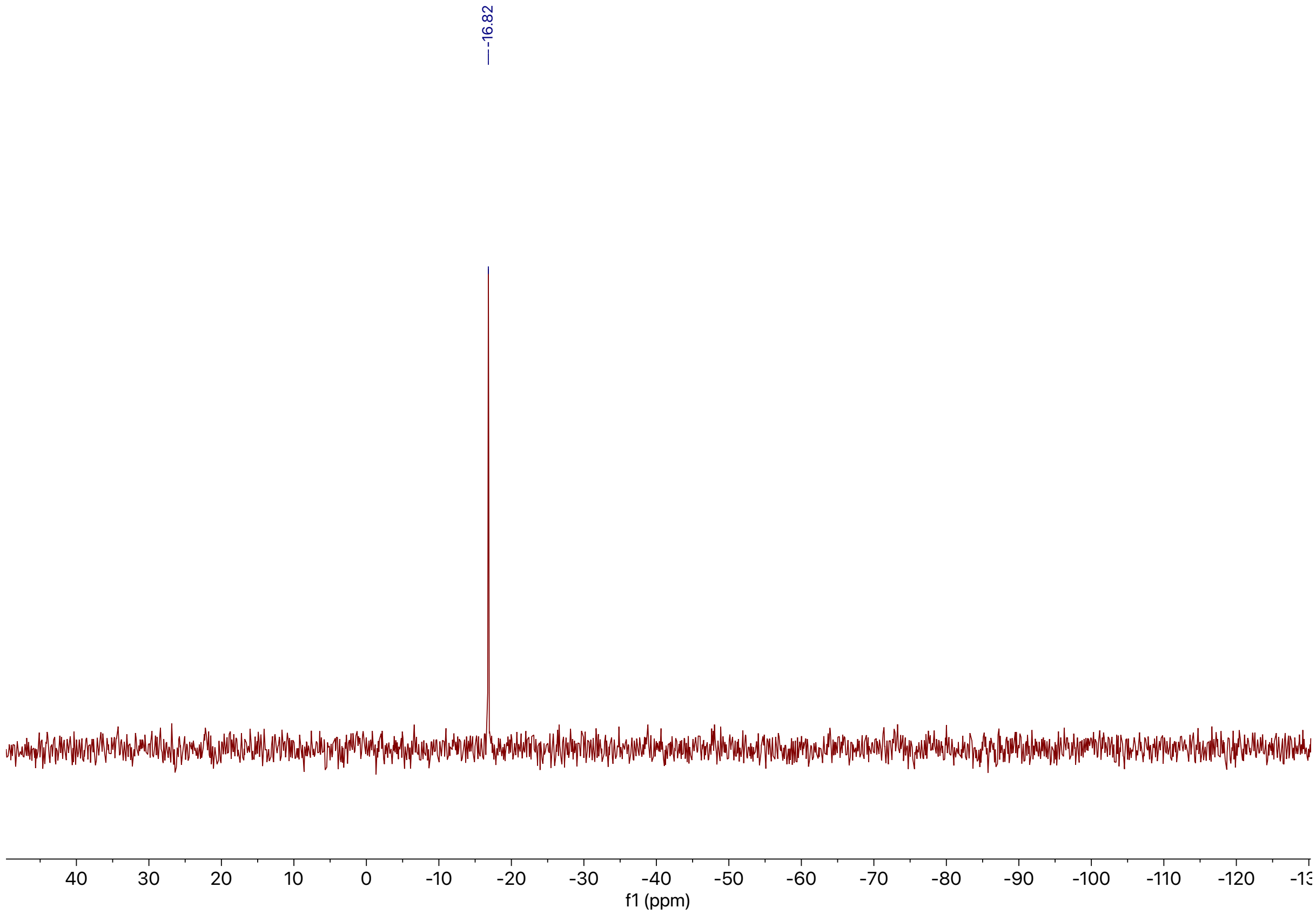
-16.82
40
30
20
10
0
-10
-20
-30
-40
-50
-60
-70
-80
-90
-100
-110
-120
f1 (ppm)

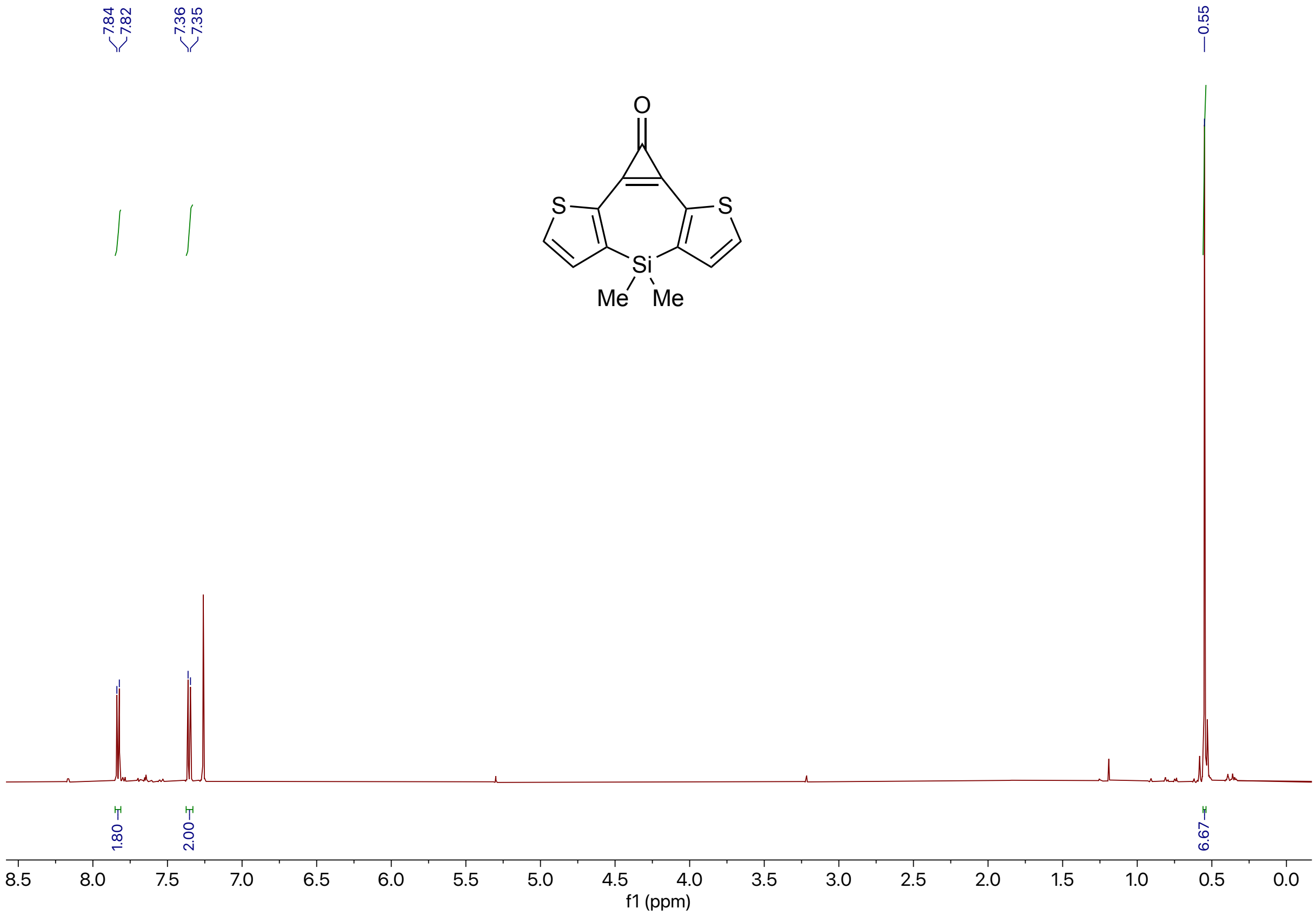
7.84
7.82
7.36
7.35
0.55
O
S
S
Si
Me
Me
1.80
2.00
6.67
8.5 8.0 7.5 7.0 6.5 6.0 5.5 5.0 4.5 4.0 3.5 3.0 2.5 2.0 1.5 1.0 0.5 0.0
f1 (ppm)

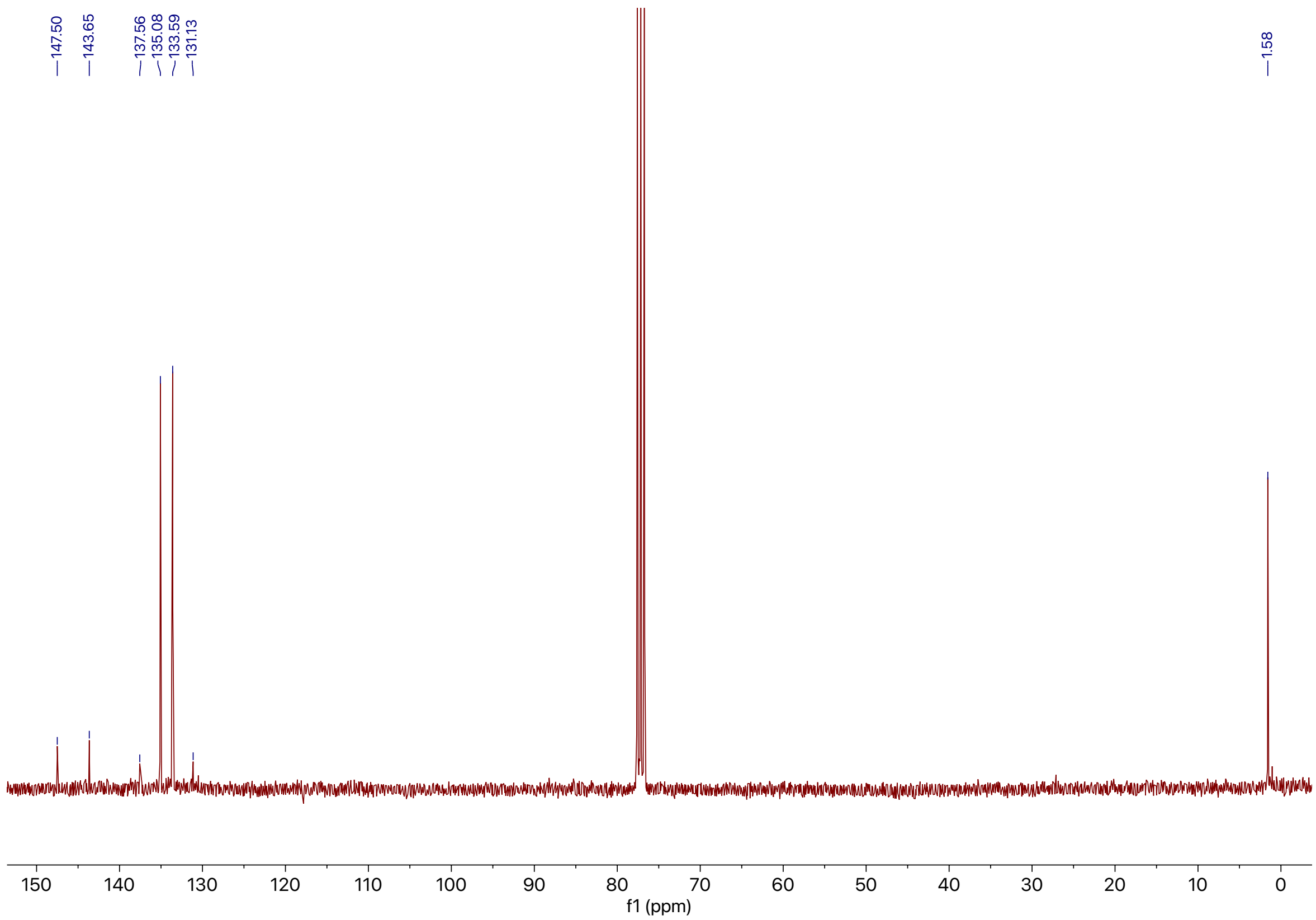
147.50
143.65
137.56
135.08
133.59
131.13
1.58
150 140 130 120 110 100 90 80 70 60 50 40 30 20 10 0
f1 (ppm)

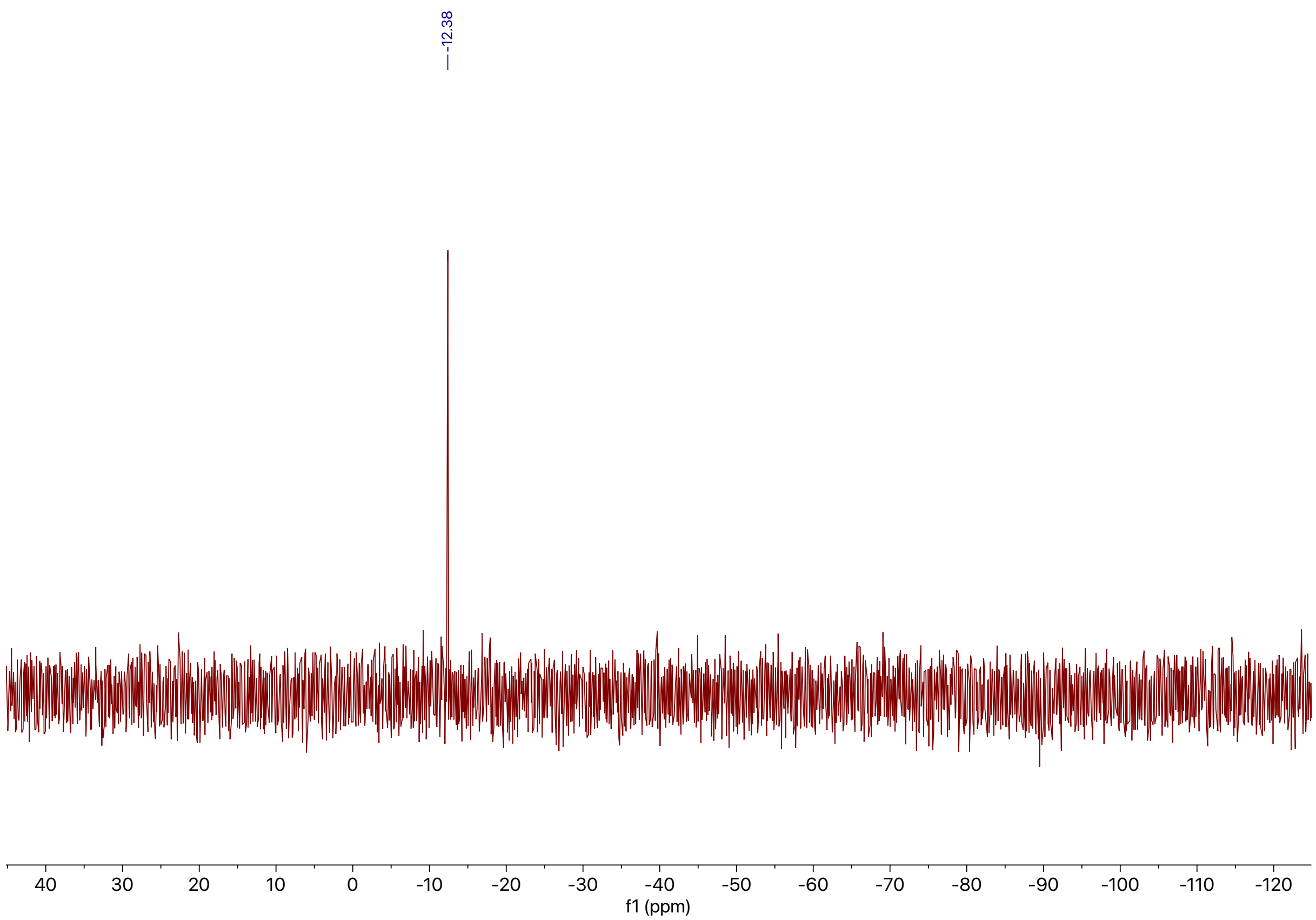
-12.38
40
30
20
10
0
-10
-20
-30
-40
-50
-60
-70
-80
-90
-100
-110
-120
f1 (ppm)

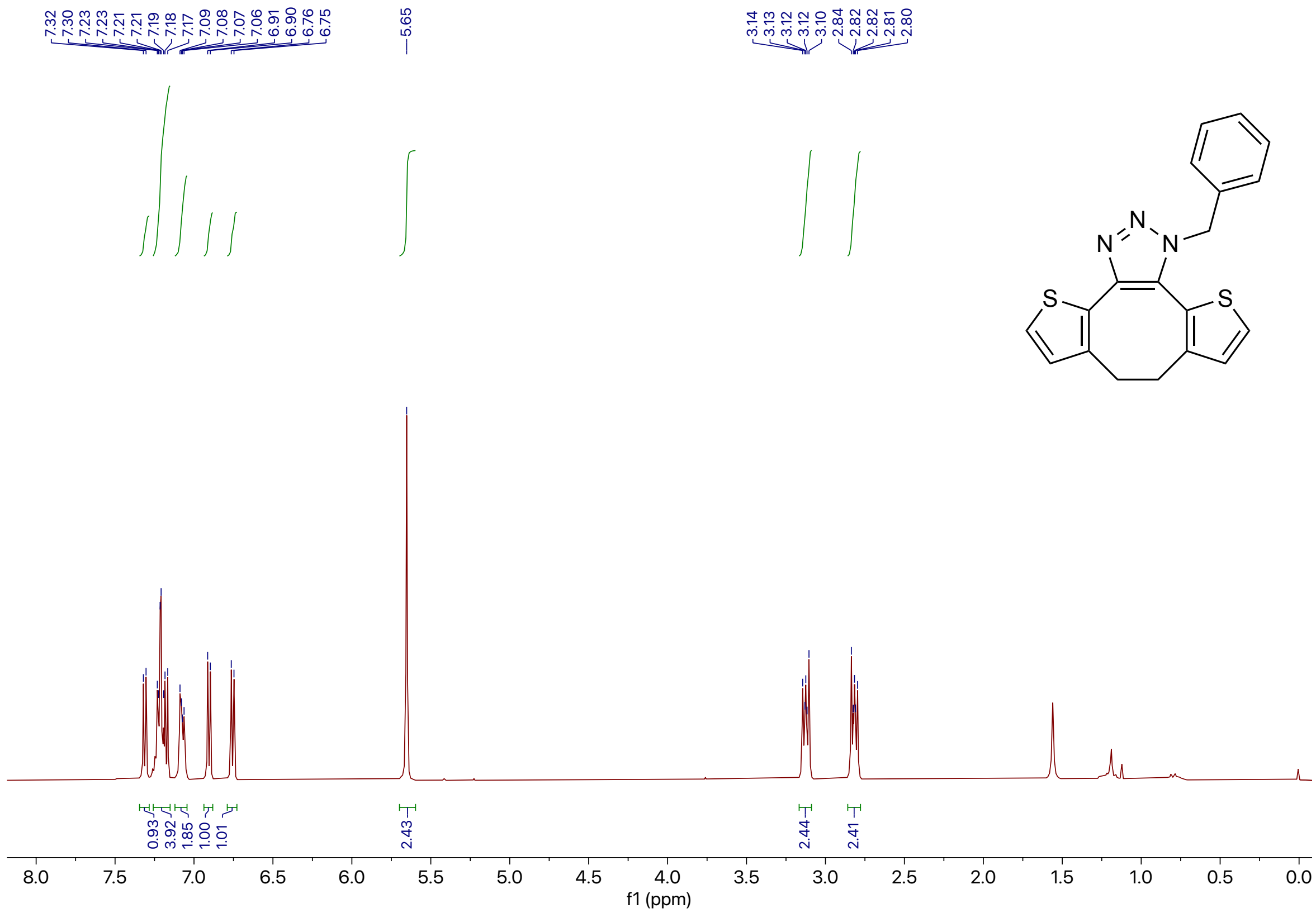
7.32
7.30
7.23
7.23
7.21
7.21
7.19
7.18
7.17
7.09
7.08
7.07
7.06
6.91
6.90
6.76
6.75
5.65
3.14
3.13
3.12
3.12
3.10
2.84
2.82
2.82
2.81
2.80
N
N
N
S
S
0.93
3.92
1.85
1.00
1.01
2.43
2.44
2.41
f1 (ppm)

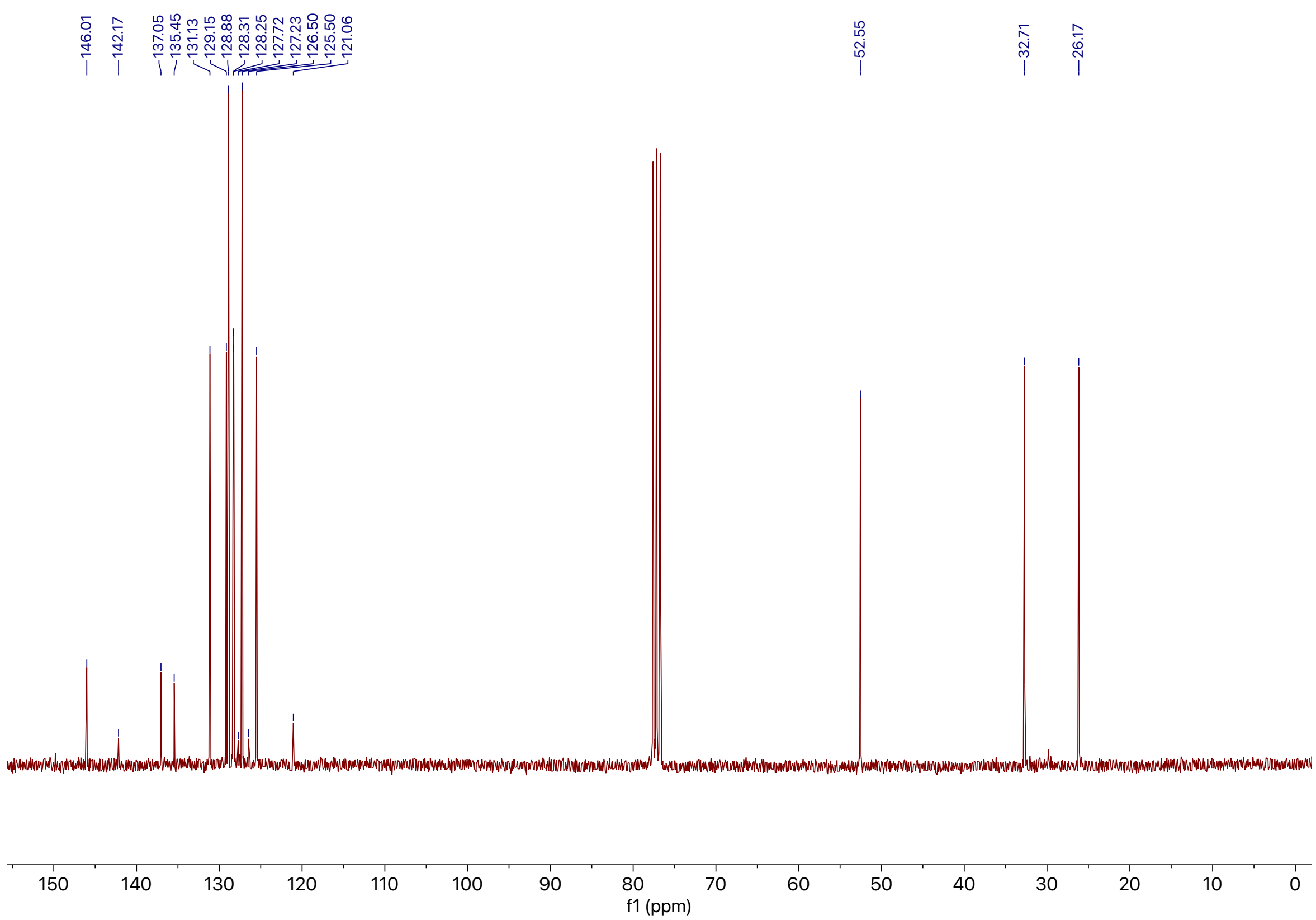
146.01
142.17
137.05
135.45
131.13
129.15
128.88
128.31
128.25
127.72
127.23
126.50
125.50
121.06
52.55
32.71
26.17
f1 (ppm)

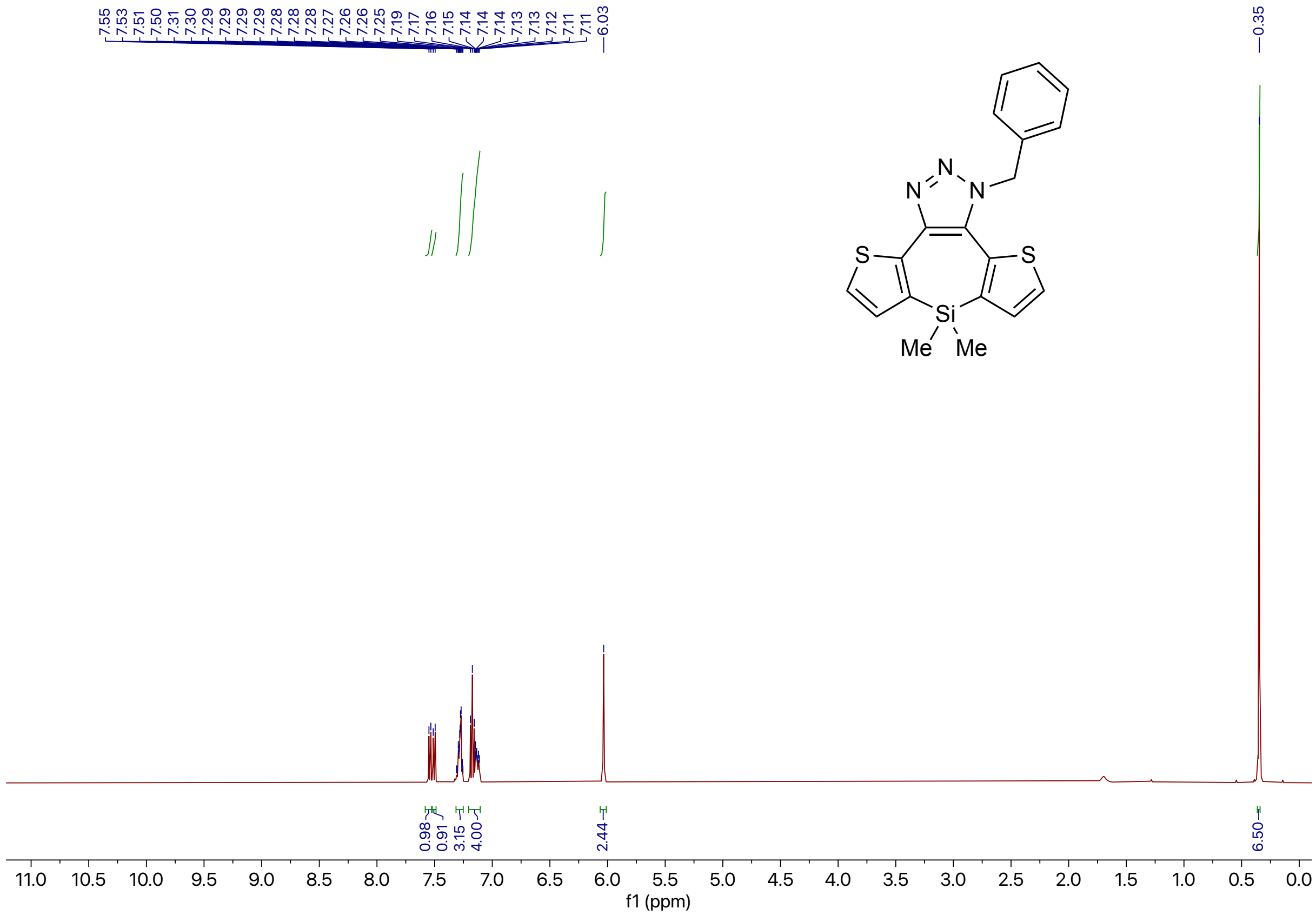
7.55 7.53 7.51 7.50 7.31 7.30 7.29 7.29 7.29 7.28 7.28 7.28 7.27 7.26 7.26 7.25 7.19 7.17 7.16 7.15 7.14 7.14 7.14 7.13 7.13 7.12 7.11
6.03
0.35
N
N
N
S
S
Si
Me
Me
0.98
0.91
3.15
4.00
2.44
6.50
11.0 10.5 10.0 9.5 9.0 8.5 8.0 7.5 7.0 6.5 6.0 5.5 5.0 4.5 4.0 3.5 3.0 2.5 2.0 1.5 1.0 0.5 0.0
f1 (ppm)

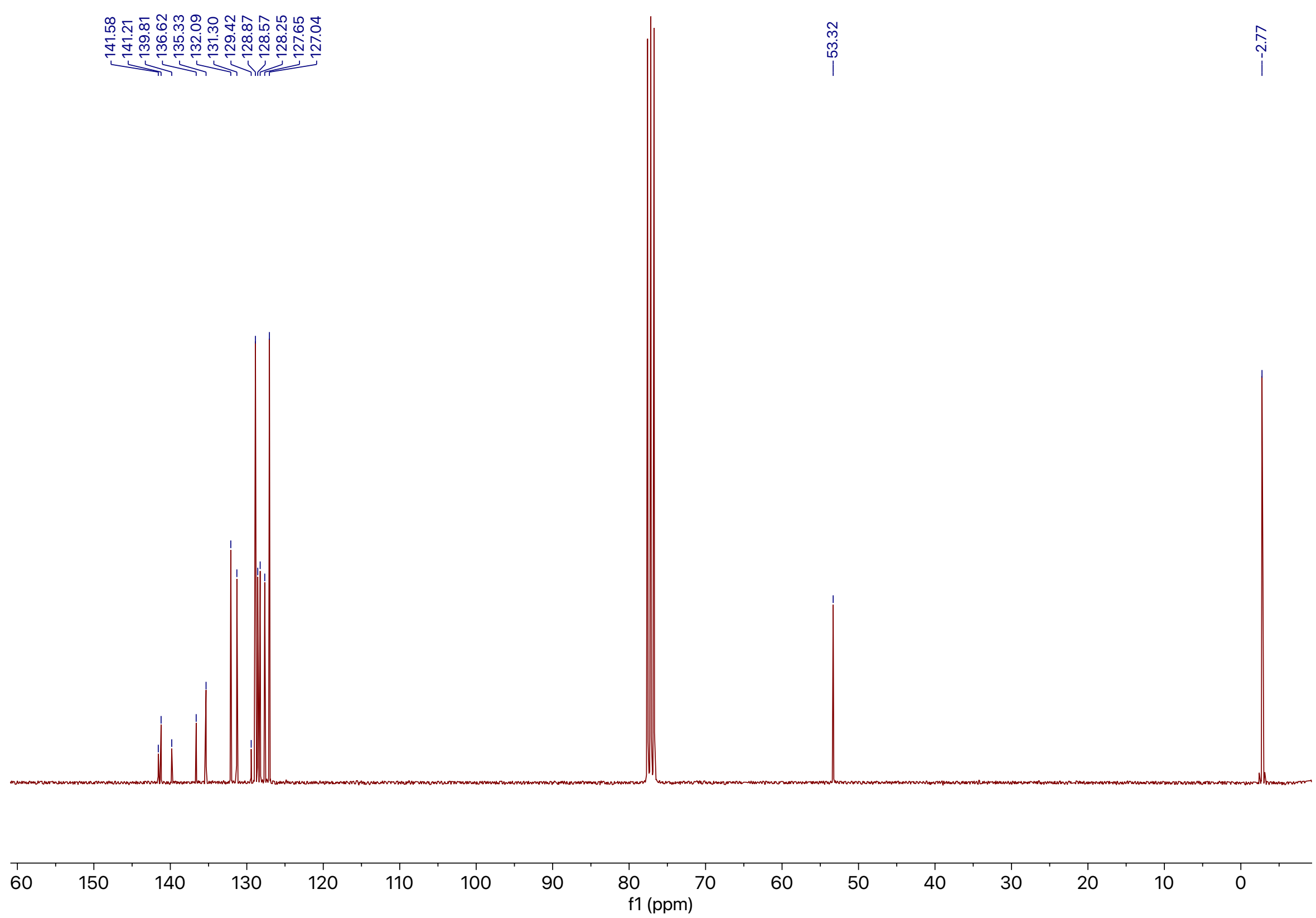
141.58 141.21 139.81 136.62 135.33 132.09 131.30 129.42 128.87 128.57 128.25 127.65 127.04
53.32
-2.77
60 150 140 130 120 110 100 90 80 70 60 50 40 30 20 10 0
f1 (ppm)

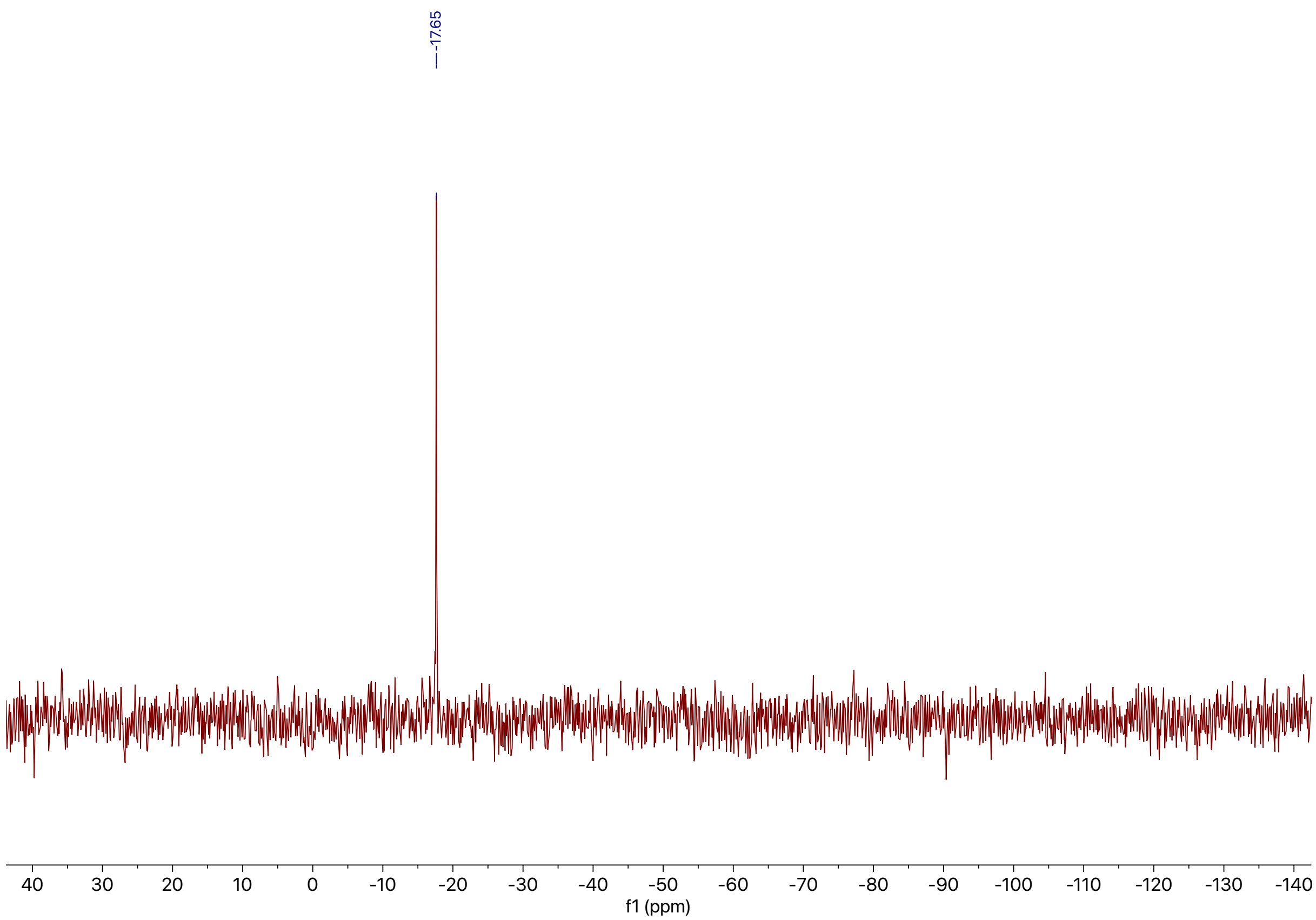
-17.65
40
30
20
10
0
-10
-20
-30
-40
-50
-60
-70
-80
-90
-100
-110
-120
-130
-140
f1 (ppm)

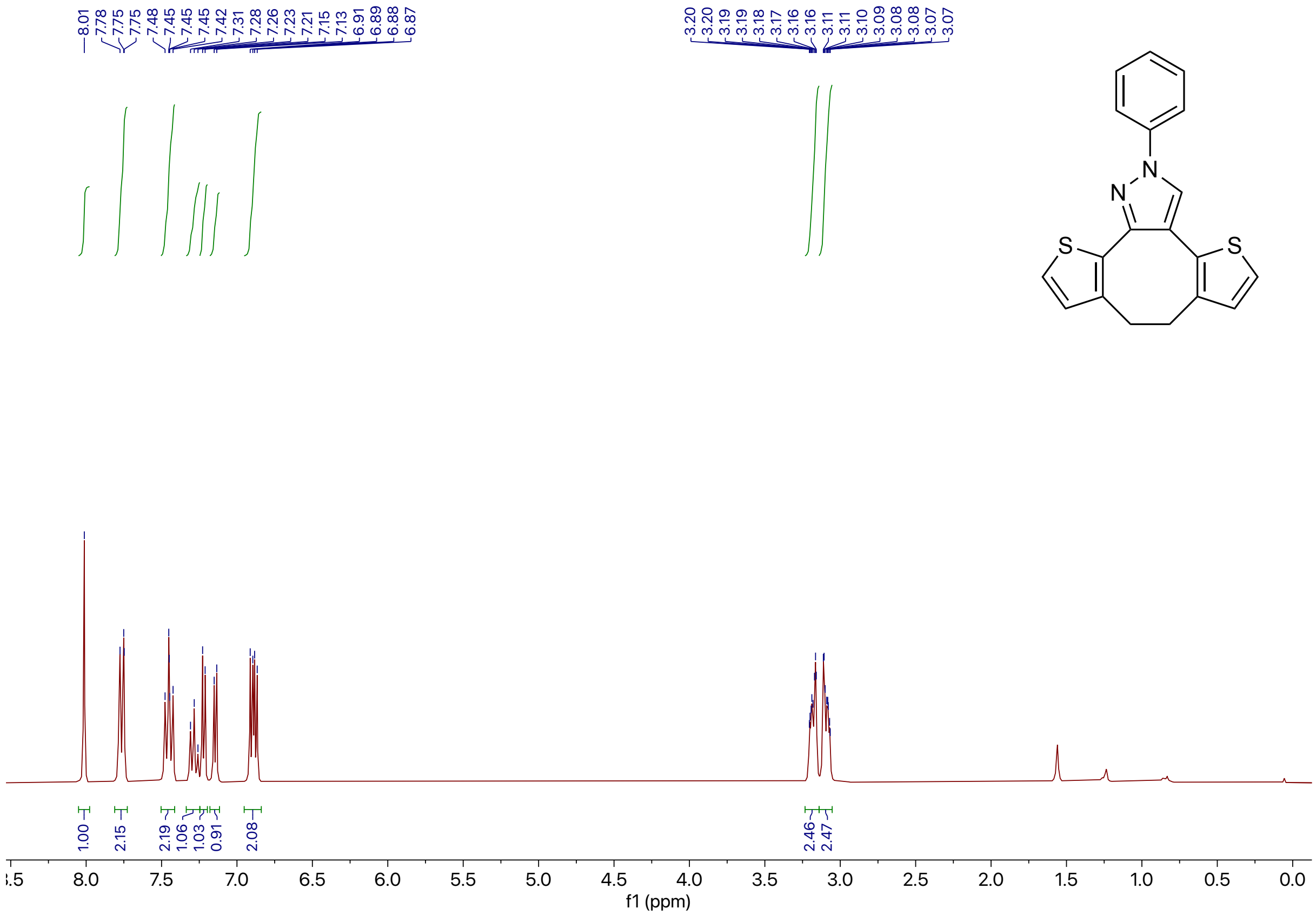
8.01
7.78
7.75
7.75
7.48
7.45
7.45
7.45
7.42
7.31
7.28
7.26
7.23
7.21
7.15
7.13
6.91
6.89
6.88
6.87
3.20
3.20
3.19
3.18
3.17
3.16
3.16
3.11
3.11
3.10
3.09
3.08
3.08
3.07
3.07
S
S
N
N
1.00
2.15
2.19
1.06
1.03
0.91
2.08
2.46
2.47
f1 (ppm)

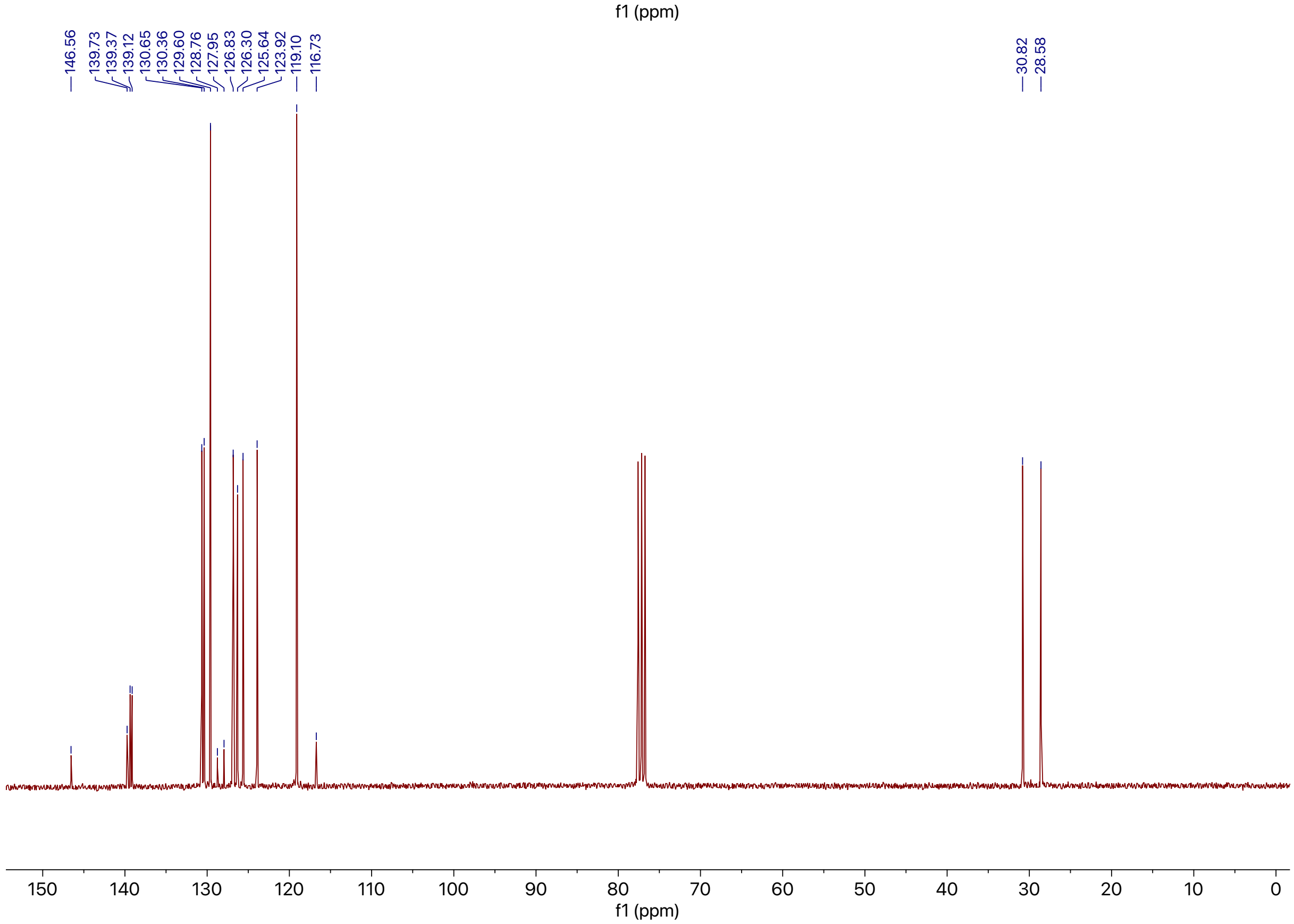
146.56
139.73
139.37
139.12
130.65
130.36
129.60
128.76
127.95
126.83
126.30
125.64
123.92
119.10
116.73
30.82
28.58
f1 (ppm)

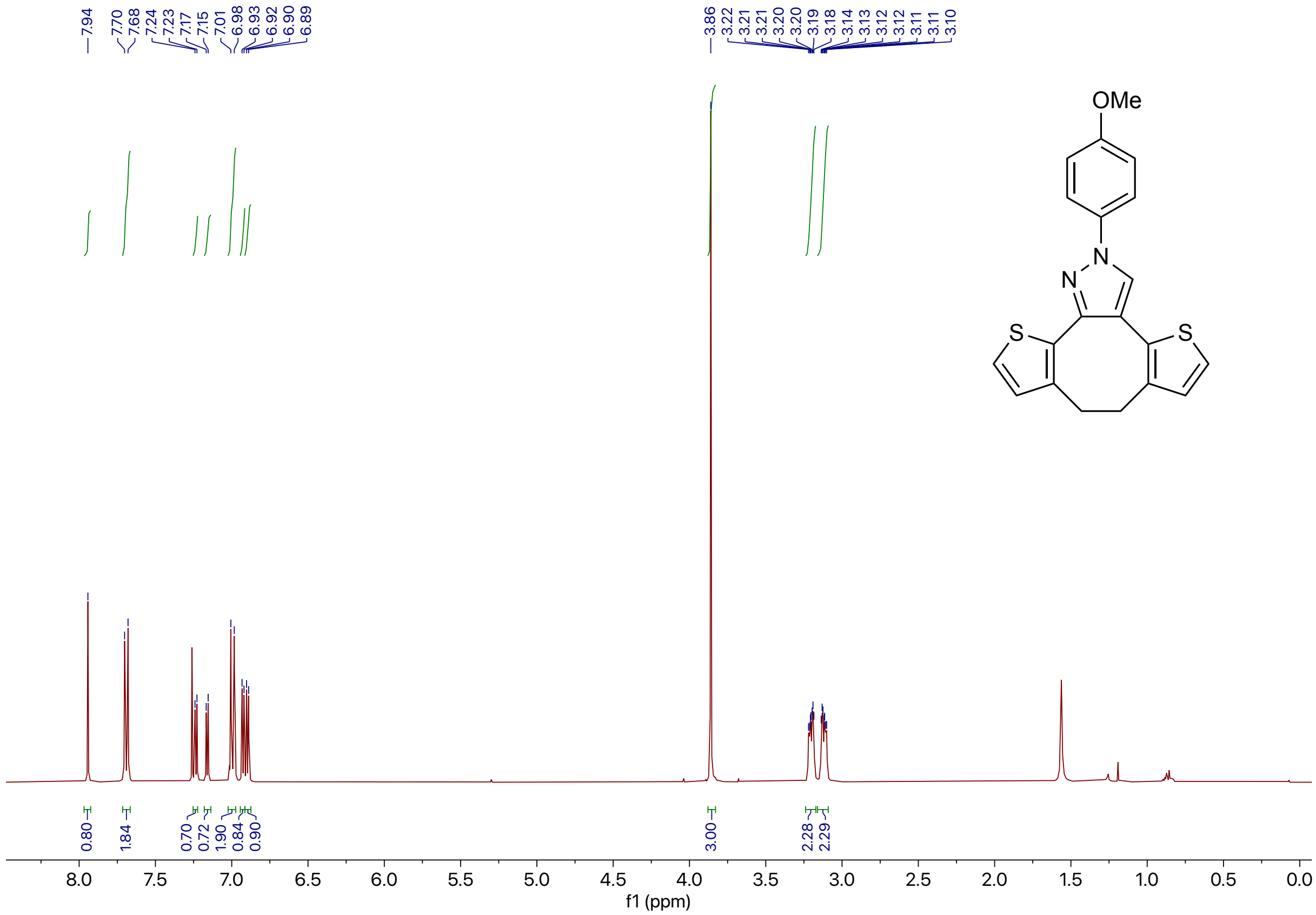
7.94
7.70
7.68
7.24
7.23
7.17
7.15
7.01
6.98
6.93
6.92
6.90
6.89
3.86
3.22
3.21
3.21
3.20
3.20
3.19
3.18
3.14
3.13
3.12
3.12
3.11
3.11
3.10
0.80
1.84
0.70
0.72
1.90
0.84
0.90
3.00
2.28
2.29
8.0 7.5 7.0 6.5 6.0 5.5 5.0 4.5 4.0 3.5 3.0 2.5 2.0 1.5 1.0 0.5 0.0
f1 (ppm)
OMe
N
N
S
S

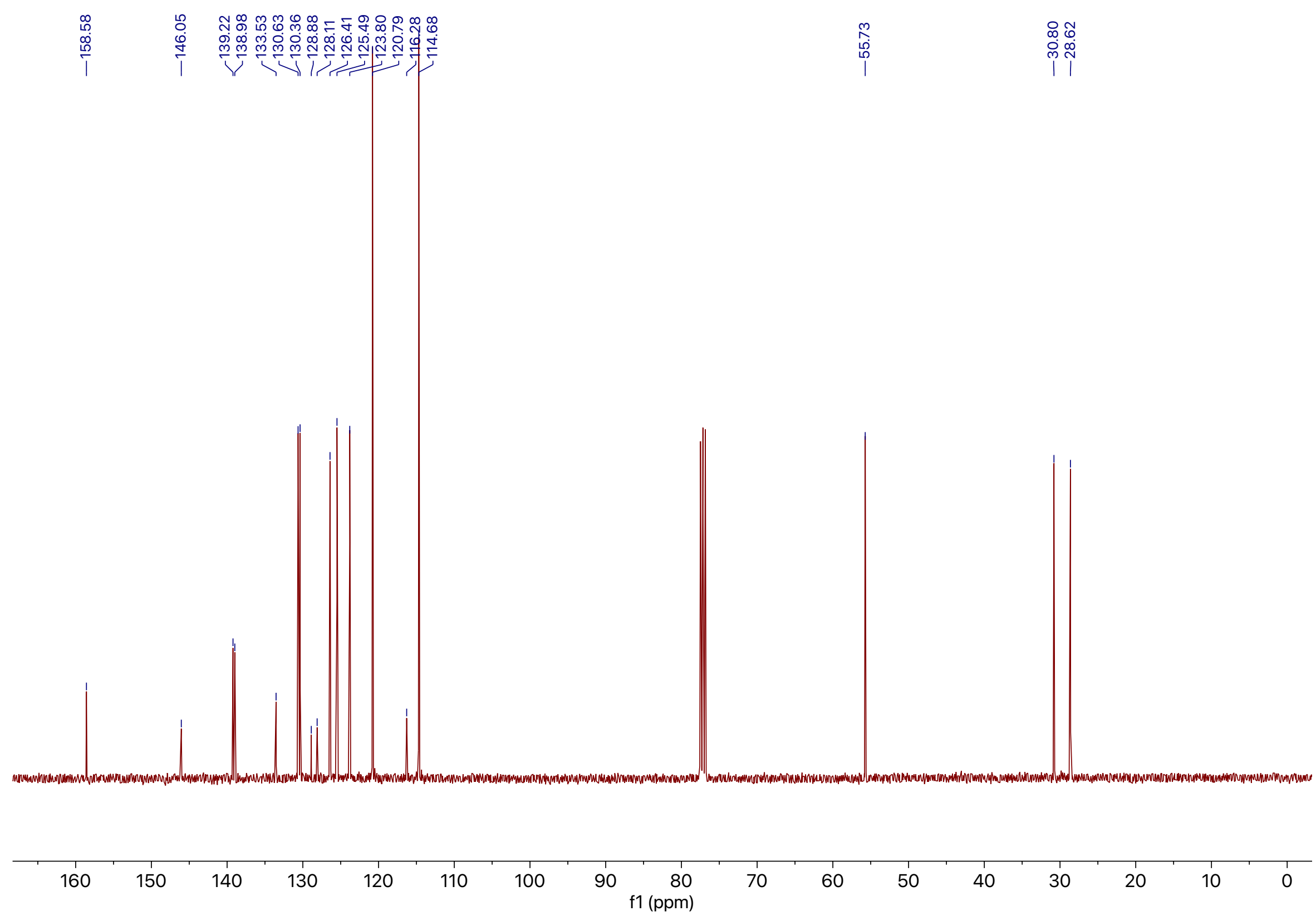
158.58
146.05
139.22
138.98
133.53
130.63
130.36
128.88
128.11
126.41
125.49
123.80
120.79
116.28
114.68
55.73
30.80
28.62
160 150 140 130 120 110 100 90 80 70 60 50 40 30 20 10 0
f1 (ppm)

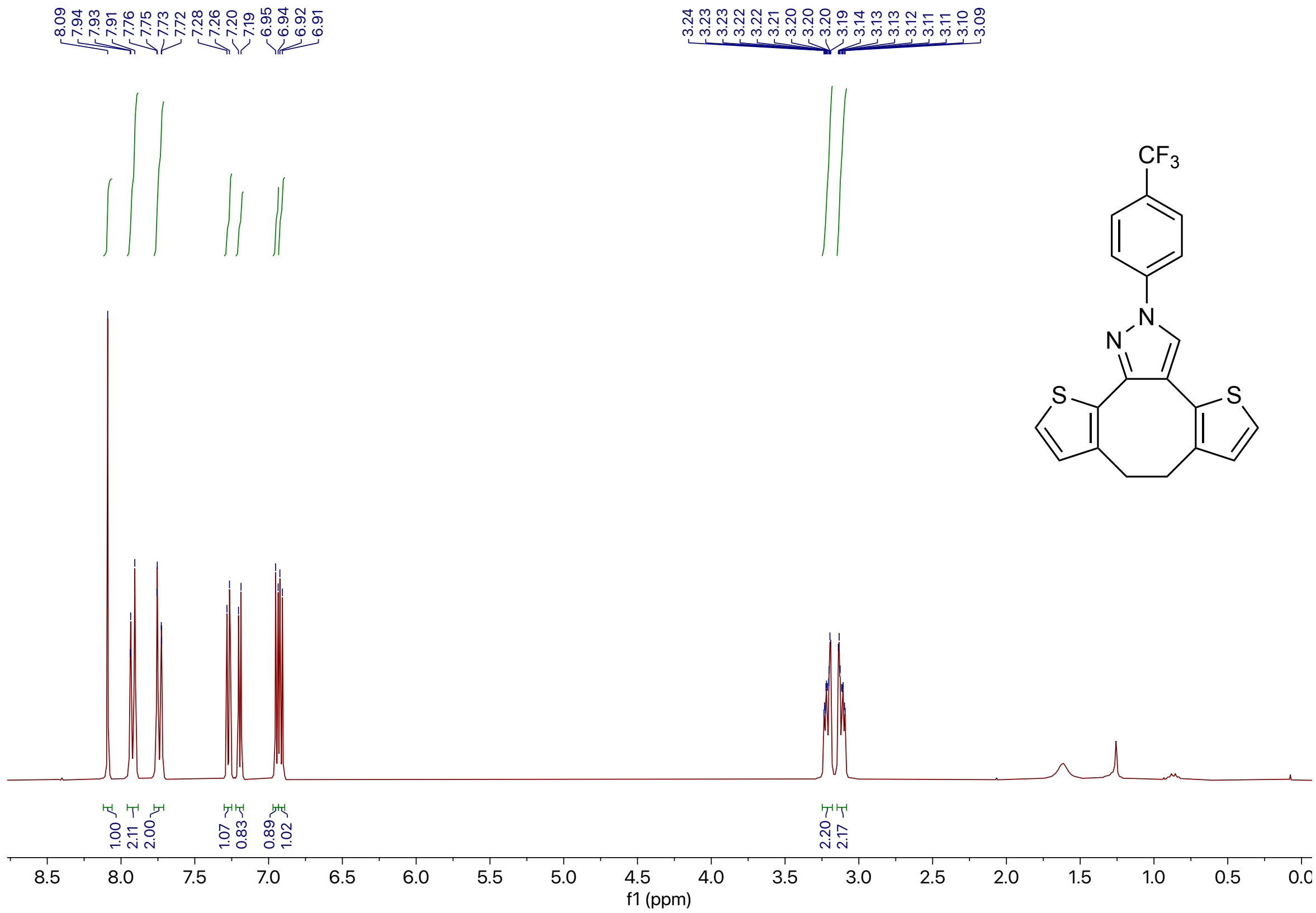
8.09 7.94 7.93 7.91 7.76 7.75 7.73 7.72 7.28 7.26 7.20 7.19 6.95 6.94 6.92 6.91
3.24 3.23 3.23 3.22 3.22 3.21 3.20 3.20 3.20 3.19 3.14 3.13 3.13 3.12 3.11 3.11 3.10 3.09
CF3
N
N
S
S
1.00 2.11 2.00 1.07 0.83 0.89 1.02
2.20 2.17
8.5 8.0 7.5 7.0 6.5 6.0 5.5 5.0 4.5 4.0 3.5 3.0 2.5 2.0 1.5 1.0 0.5 0.0
f1 (ppm)

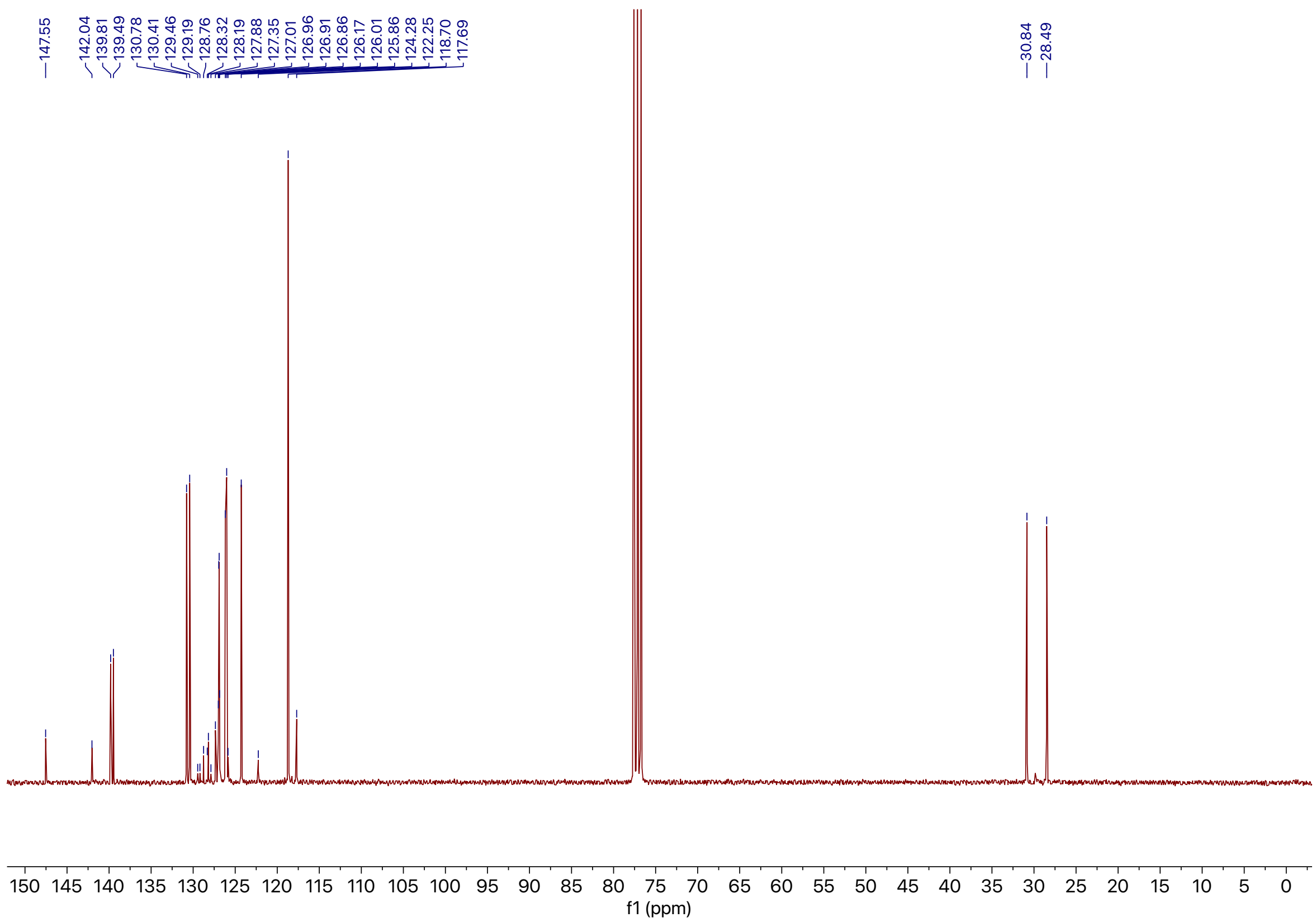
147.55
142.04 139.81 139.49 130.78 130.41 129.46 129.19 128.76 128.32 128.19 127.88 127.35 127.01 126.96 126.91 126.86 126.17 126.01 125.86 124.28 122.25 118.70 117.69
30.84 28.49
150 145 140 135 130 125 120 115 110 105 100 95 90 85 80 75 70 65 60 55 50 45 40 35 30 25 20 15 10 5 0
f1 (ppm)

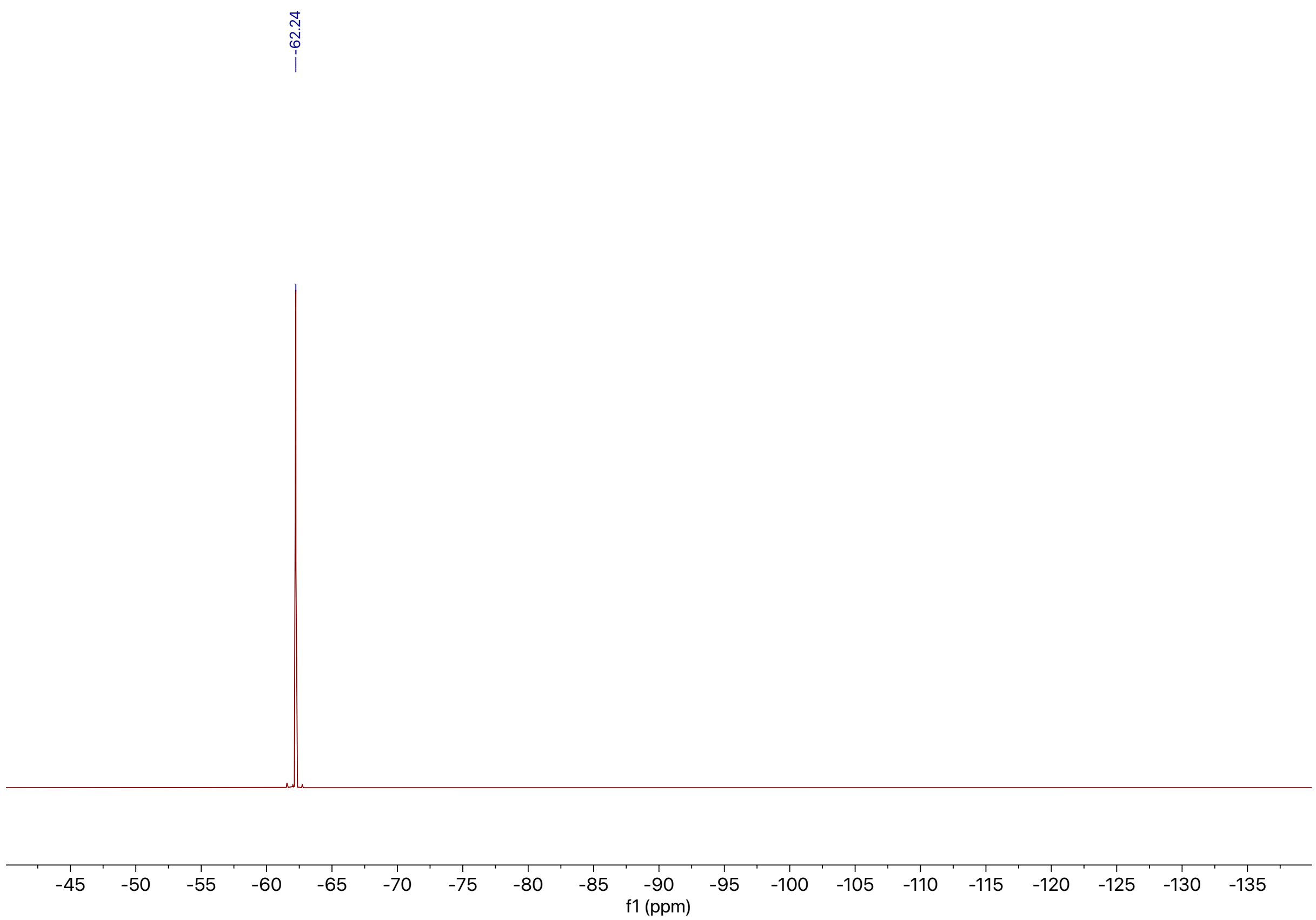
-62.24
-45
-50
-55
-60
-65
-70
-75
-80
-85
-90
-95
-100
-105
-110
-115
-120
-125
-130
-135
f1 (ppm)

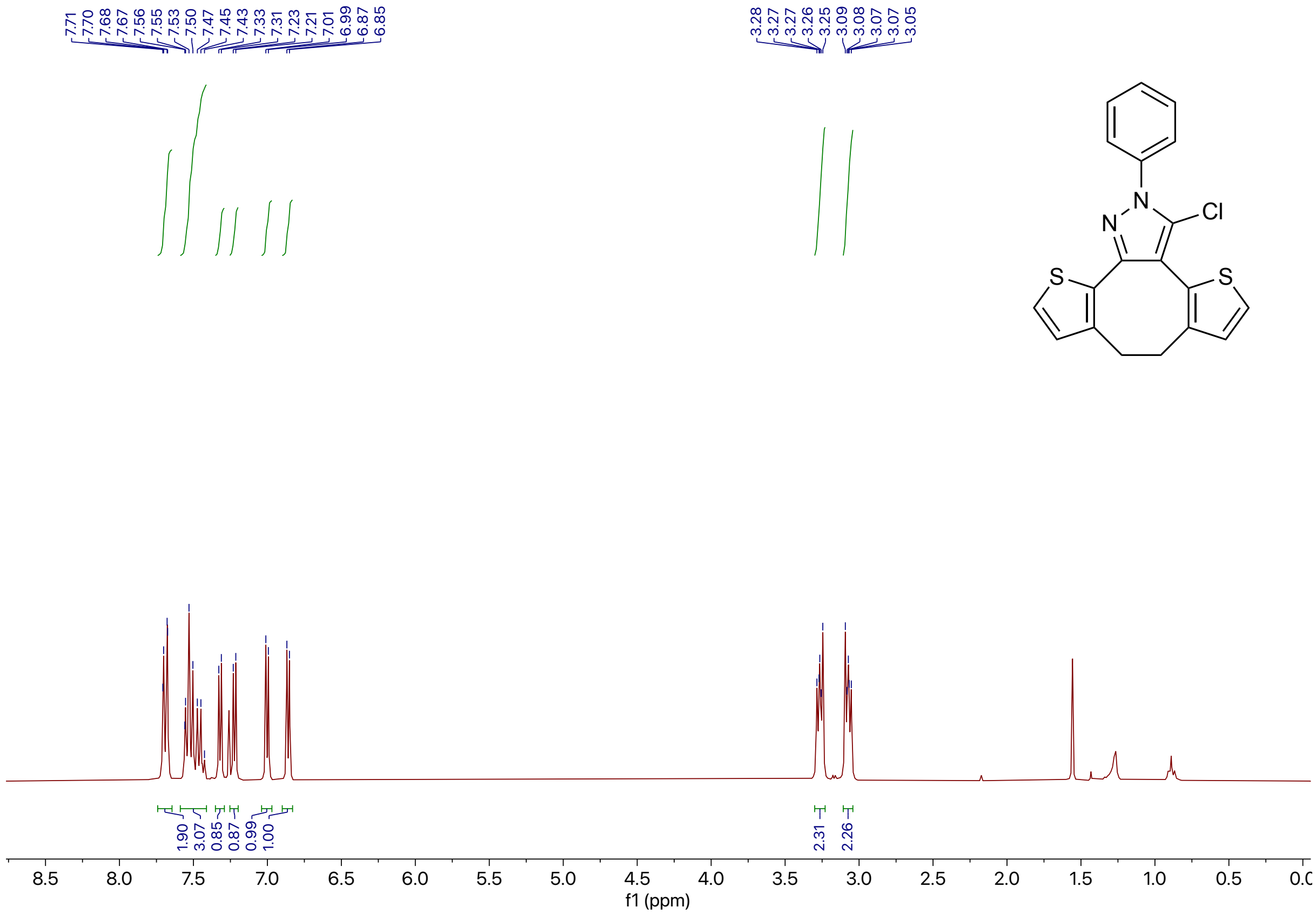

7.71
7.70
7.68
7.67
7.56
7.55
7.53
7.50
7.47
7.45
7.43
7.33
7.31
7.23
7.21
7.01
6.99
6.87
6.85
3.28
3.27
3.27
3.26
3.25
3.09
3.08
3.07
3.07
3.05
N
N
Cl
S
S
1.90
3.07
0.85
0.87
0.99
1.00
2.31
2.26
8.5
8.0
7.5
7.0
6.5
6.0
5.5
5.0
4.5
4.0
3.5
3.0
2.5
2.0
1.5
1.0
0.5
0.0
f1 (ppm)

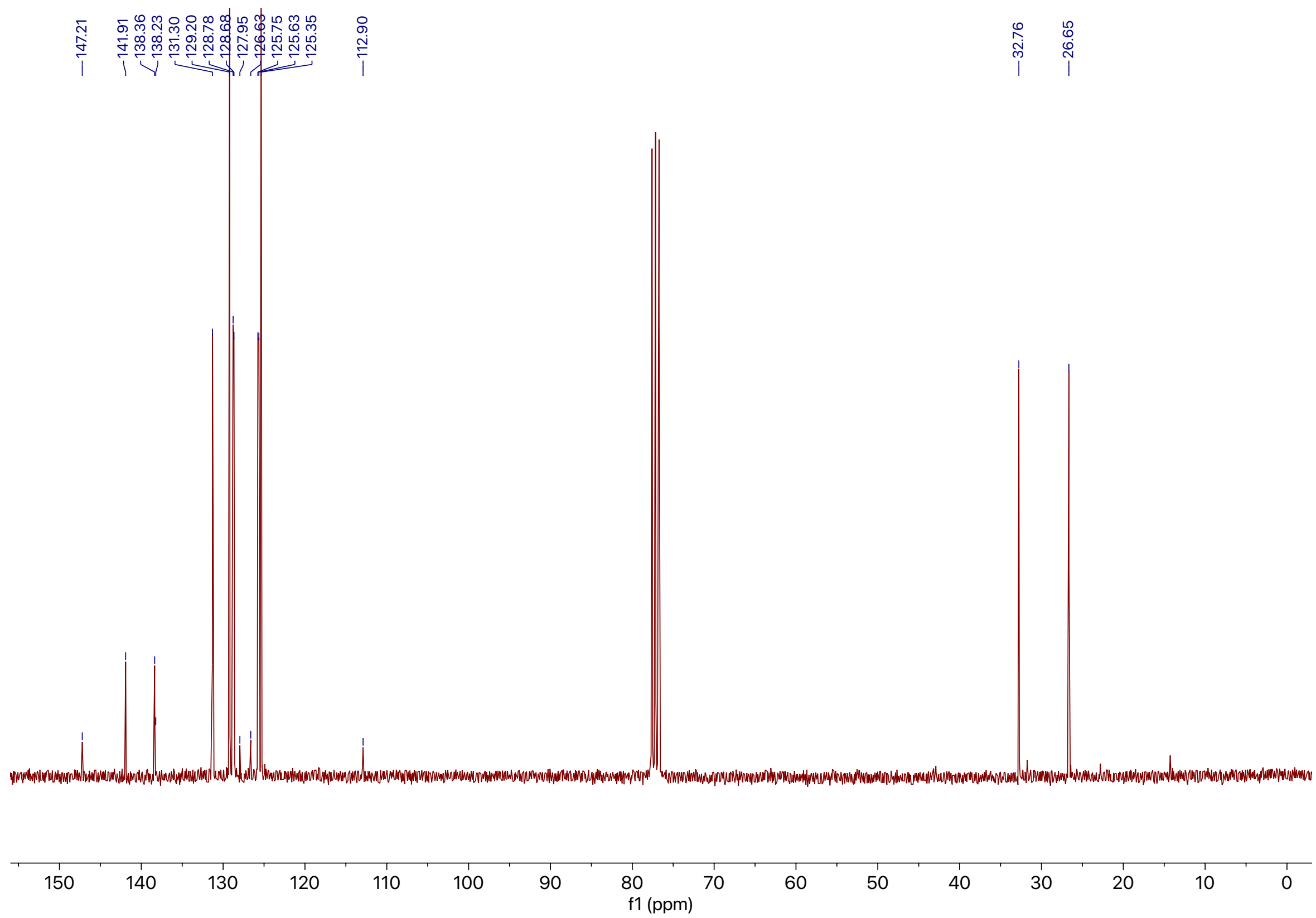

147.21
141.91
138.36
138.23
131.30
129.20
128.78
128.68
127.95
126.63
125.75
125.63
125.35
112.90
32.76
26.65
150
140
130
120
110
100
90
80
70
60
50
40
30
20
10
0
f1 (ppm)

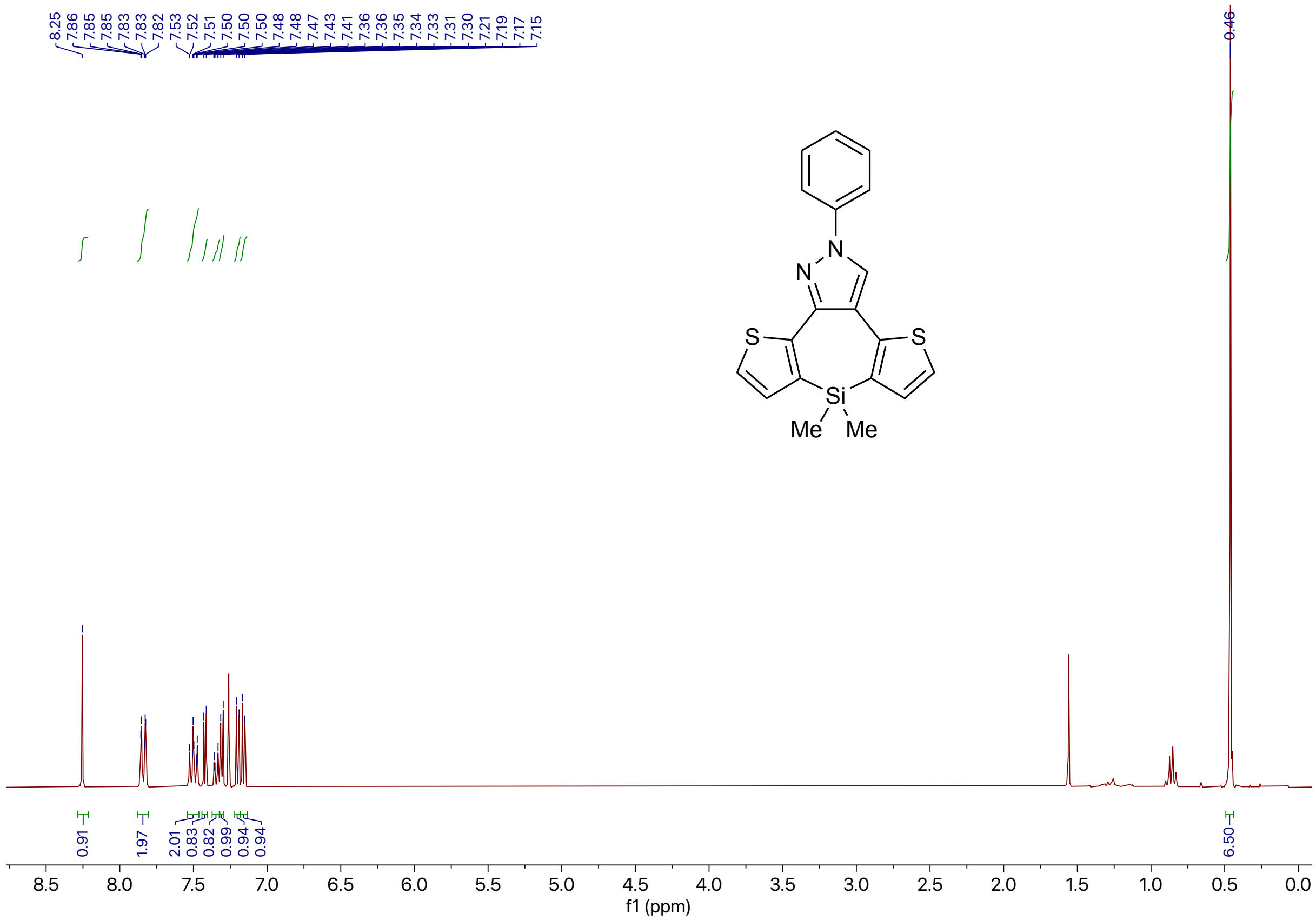
8.25
7.86
7.85
7.85
7.83
7.83
7.82
7.53
7.52
7.51
7.50
7.50
7.48
7.48
7.47
7.43
7.41
7.36
7.36
7.35
7.34
7.33
7.31
7.30
7.21
7.19
7.17
7.15
0.46
N
N
S
S
Si
Me
Me
0.91
1.97
2.01
0.83
0.82
0.99
0.94
0.94
6.50
f1 (ppm)

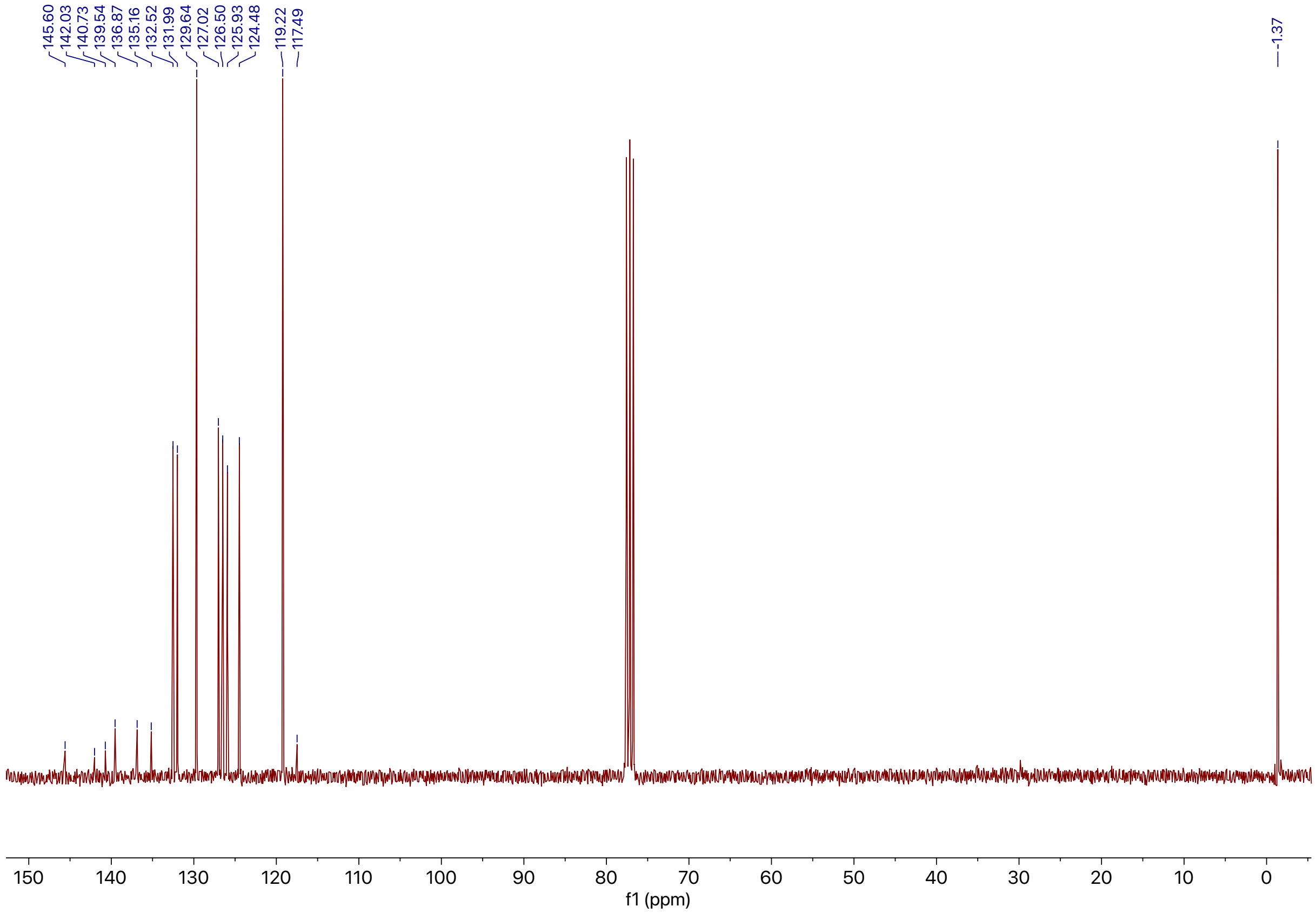
145.60
142.03
140.73
139.54
136.87
135.16
132.52
131.99
129.64
127.02
126.50
125.93
124.48
119.22
117.49
-1.37
f1 (ppm)

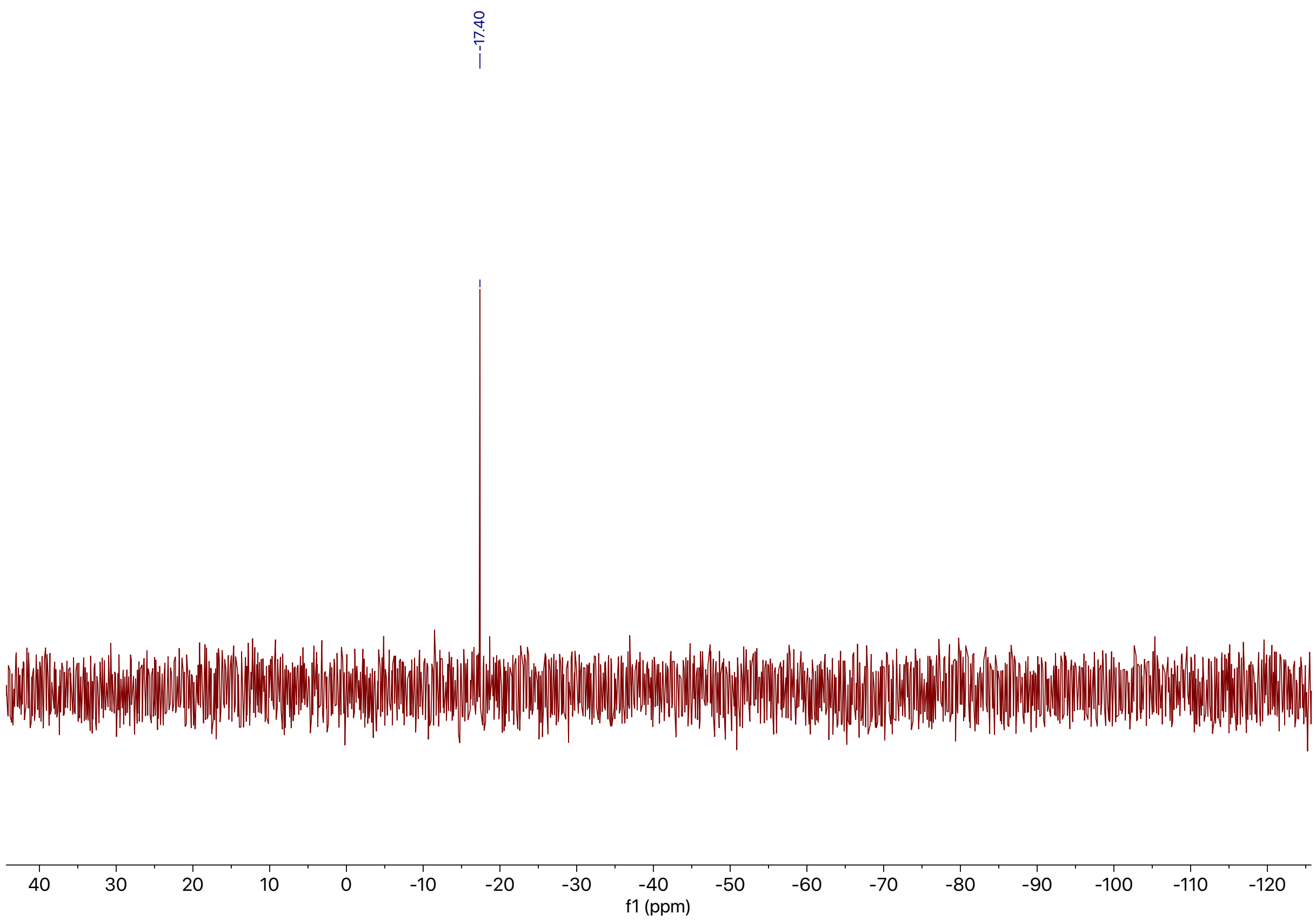
-17.40
40
30
20
10
0
-10
-20
-30
-40
-50
-60
-70
-80
-90
-100
-110
-120
f1 (ppm)

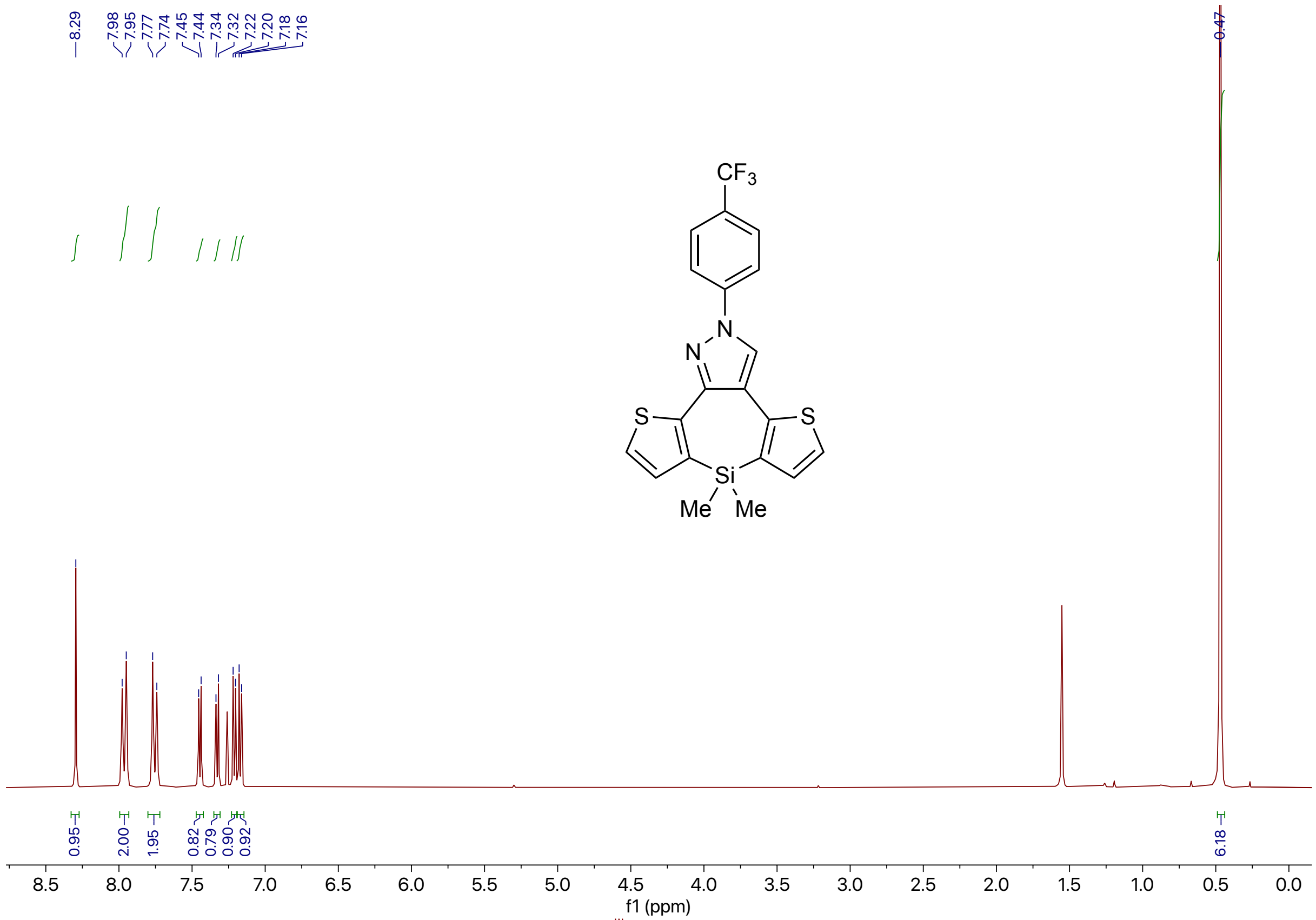
8.29
7.98
7.95
7.77
7.74
7.45
7.44
7.34
7.32
7.22
7.20
7.18
7.16
0.47
CF3
N
N
S
S
Si
Me
Me
0.95
2.00
1.95
0.82
0.79
0.90
0.92
6.18
f1 (ppm)

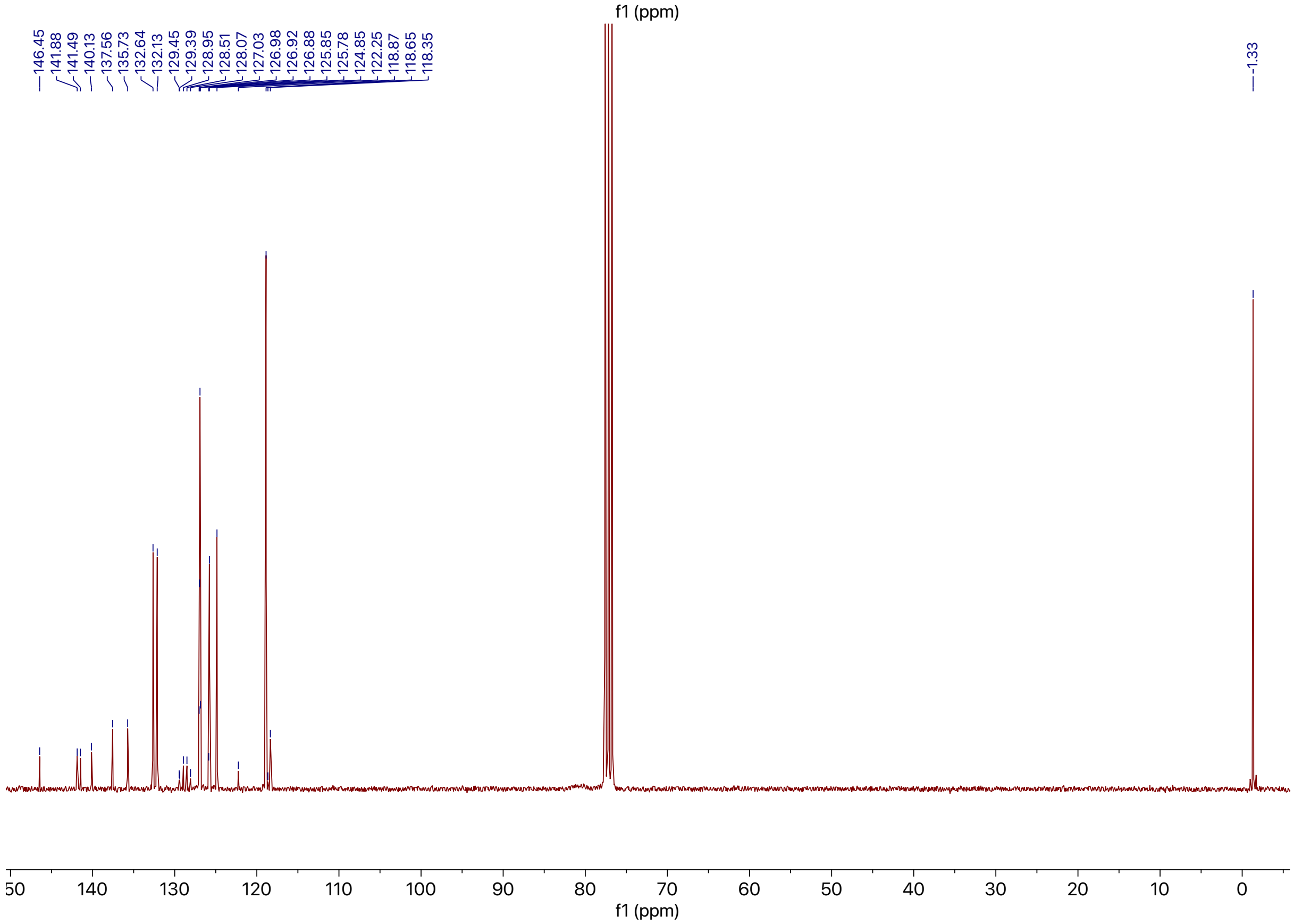
146.45
141.88
141.49
140.13
137.56
135.73
132.64
132.13
129.45
129.39
128.95
128.51
128.07
127.03
126.98
126.92
126.88
125.85
125.78
124.85
122.25
118.87
118.65
118.35
-1.33
f1 (ppm)

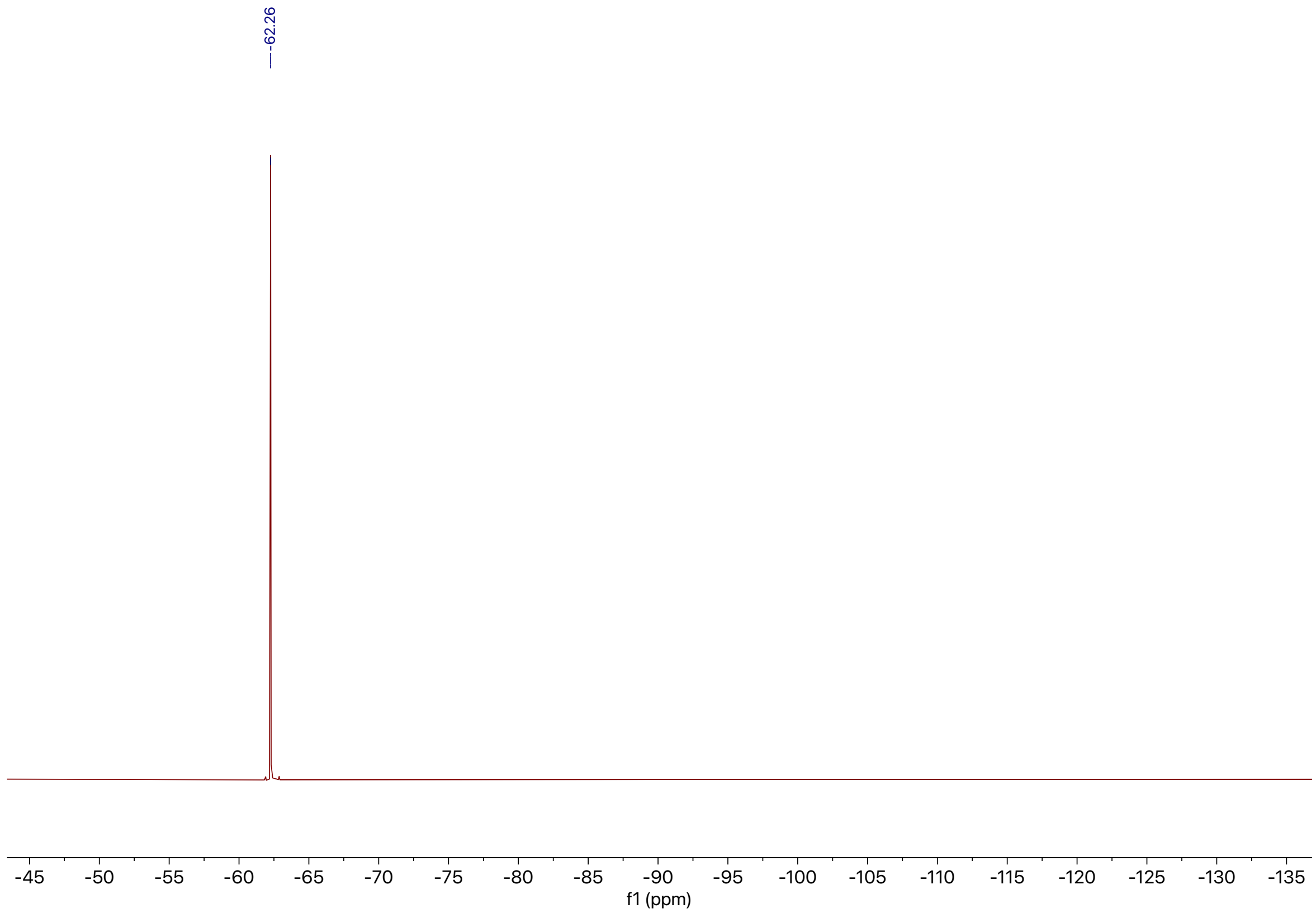
-62.26
-45
-50
-55
-60
-65
-70
-75
-80
-85
-90
-95
-100
-105
-110
-115
-120
-125
-130
-135
f1 (ppm)

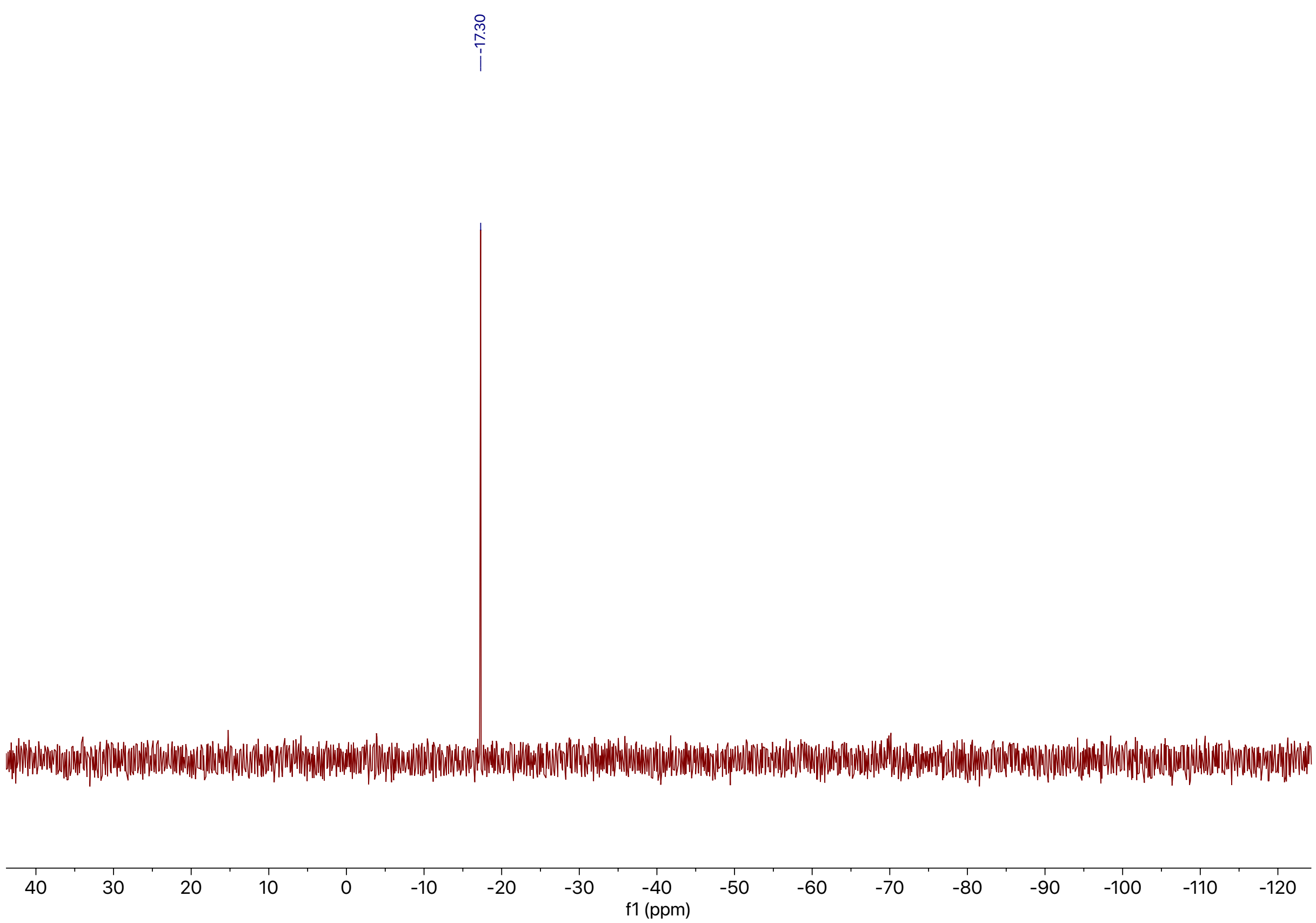
-17.30
40
30
20
10
0
-10
-20
-30
-40
-50
-60
-70
-80
-90
-100
-110
-120
f1 (ppm)

References.

(1) Frisch, M. J., et al. Gaussian 16 Revision C.01. 2019.

(2) (a) Grimme, S. Semiempirical GGA-type density functional constructed with a long-range dispersion correction. *J. Comput. Chem.* **2006**, *27*, 1787-1799; (b) Becke, A.D. Density-functional thermochemistry. V. Systematic optimization of exchange-correlation functionals. *J. Chem. Phys.* **1997**, *107*, 8554-8560; (c) Chai, J.D.; Gordon, M. Long-range corrected hybrid density functionals with damped atom-atom dispersion corrections. *Phys. Chem. Chem. Phys.* **2008**, *10*, 6615-6620; (d) Wu, Q.; Yang, W.T. Empirical correction to density functional theory for van der Waals interactions. *J. Chem. Phys.* **2002**, *116*, 515-524

(3) (a) Ditchfield R, Hehre W J, Pople J A. *J. Chem. Phys.* **1971**, *54*(2): 724-728; (b) Hehre W J, Ditchfield R, Pople J A. *J. Chem. Phys.* **1972**, *56*(5): 2257-2261; (c) Pople J A, Binkley J S, Seeger R. *International Journal of Quantum Chemistry*, **1976**, *10*(S10): 1-19.

(4) (a) Klamt, A.; Schüürmann, G. *J. Chem. Soc., Perkin Trans. 2* **1993**, 799; (b) Andzelm, J.; Kölmel, C.; Klamt, A. *J. Chem. Phys.***1995**, *103*, 9312-9320; (c) Barone, V.; Cossi, M. *J. Phys. Chem. A* **1998**, *102*, 1995-001; (d) Cossi, M.; Rega, N.; Scalmani, G.; Barone, V. *J. Comput. Chem.* **2003**, *24*, 669-681.

(5) (a) Krishnan R, Binkley J S, Seeger R, et al. *J. Chem. Phys.* **1980**, *72*(1): 650-654. (b) McLean A D, Chandler G S. *J. Chem. Phys.* **1980**, *72*(10): 5639-5648.

(6) Fukui, K. Formulation of the Reaction Coordinate. *J. Phys. Chem.* **1970**, *74*, 4161−4163.

(7) Fukui, K. The Path of Chemical Reactions-the IRC Approach. *Acc. Chem. Res.* **1981**, *14*, 363−368.

(8) Luchini, G.; Alegre-Requena, J. V.; Funes-Ardoiz, I.; Paton, R. S. GoodVibes: automated thermochemistry for heterogeneous computational chemistry data [version 1; peer review: 2 approved with reservations] 2020, 9.
